# Neofunctionalization of H1 linker histones drives divergent gene expression and histone methylation in *Cryptococcus neoformans*

**DOI:** 10.1038/s42003-026-10432-4

**Published:** 2026-06-10

**Authors:** Grace J. Paul, Nicolas Helmstetter, Qinxi Ma, Diana Tamayo, Tal Goodisman, James W. Harrison, Elizabeth R. Ballou, Rhys A. Farrer

**Affiliations:** Medical Research Council Centre for Medical Mycology at the University of Exeter, Department of Biosciences, Faculty of Health and Life Sciences, Exeter, UK

**Keywords:** Epigenomics, Evolutionary genetics

## Abstract

The opportunistic pathogen *Cryptococcus neoformans* adapts to diverse host microenvironments during infection, yet the contribution of epigenetic mechanisms remains unclear. Comparative genomics across 50 basidiomycete species revealed that one chromatin protein, the linker histone H1, is uniquely duplicated across the entire *Cryptococcus genus*, generating paralogs H1.51 and H1.52 with divergent evolutionary trajectories. Molecular evolution analyses revealed that H1.51 experienced intensified selection with episodic positive selection in clinical isolates, while H1.52 evolved under sustained purifying selection, indicating adaptive neofunctionalization. H1.52 deletion triggered transcriptional reprogramming of 1561 genes (23% of the transcriptome), with downregulation of nucleolar function, ribosome biogenesis, and translation machinery, whereas H1.51 loss produced minimal transcriptional effects. We found that H1.52 governs chromatin architecture. Its deletion expanded H3K4me2 euchromatin coverage 1.54-fold (from 18.4% to 28.4% of the genome) into broader constitutive domains with loss of H3K9-marked heterochromatin islands. Following prolonged culture under host mimicking conditions (3-, 5-, and 7-day periods), H1.52 deletion resulted in enhanced metabolic activity and accelerated growth recovery compared to wild-type. These findings establish that *Cryptococcus neoformans* H1.52 coordinates chromatin compaction, maintains H3K9-marked heterochromatin islands and is a regulator of metabolic responses to host stress.

## Abstract (web summary)

An ancient linker histone duplication in Cryptococcus produced functionally divergent paralogs. H1.52 maintains H3K9me2 heterochromatin islands, constrains H3K4me2 spreading, and restrains metabolic activity during host-mimicking stress.

## Introduction

The environmental yeast *Cryptococcus neoformans* represents a major opportunistic pathogen of immunocompromised hosts, causing life-threatening infections following inhalation. Upon entering the host lung, *C. neoformans* populations exhibit striking cellular heterogeneity encompassing diverse morphological and metabolic states. In the lung alveoli, a subset of haploid yeast cells can undergo endoreduplication into enlarged, polyploid titan cells that produce heterogeneous daughter cells with variable ploidy, conferring differential capacities for dissemination and immune evasion^1^. *C. neoformans* morphological plasticity in response to different environmental conditions encompasses diverse forms ranging from infectious basidiospores to phagocytosis-resistant titan cells, with sizes ranging from 1 to >15 μm^2,3^. In addition to morphological variation, members of the *Cryptococcus* genus, display metabolic heterogeneity spanning quiescent, stress-resistant states to actively proliferating cells^4^. Cell types include dormant spores, biofilm-associated cells, viable-but-non-culturable cells, persisters, and rapidly dividing yeast with divergent biosynthetic capacity and stress tolerance^5–8^. This morphological and metabolic heterogeneity within populations suggests that genetically similar cells are differentially regulated, leading to different cellular identities.

One possible mechanism for generating population heterogeneity is epigenetic regulation that couples environmental cues to metabolic and cellular state transitions. Chromatin structure provides a fundamental mechanism through which eukaryotic cells regulate fate transitions. In addition to core histones (H2A, H2B, H3 and H4) that form nucleosomes, linker histones (H1) bind DNA between nucleosomes to modulate chromatin compaction, transcription, and three dimensional genome organisation^9,10^. Importantly, linker histones dynamically adjust chromatin architecture in response to nutrient availability and metabolic state, positioning them as candidate mediators of phenotypic plasticity in response to host environmental signals^11^. Emerging evidence across diverse eukaryotes suggests that H1 levels modulate reversible switching between proliferative and quiescent or differentiated cell fates. For instance, quiescent human hematopoietic stem cells maintain high H1 levels, while H1 loss accompanies their transition to more proliferative, myeloid-biased states^12^. Similarly, proliferative cancer stem cells in solid tumours frequently silence H1, whereas its re-expression or pharmacological restoration drives differentiation, reduces long-term proliferative capacity, and increases chemosensitivity^13,14^. Collectively, studies across eukaryotes implicate H1 in cellular differentiation through chromatin remodelling when environmental conditions favour growth^11,15–18^.

Linker histone function varies considerably across fungal species. At one extreme, *Schizosaccharomyces pombe* lacks H1 entirely, while *Aspergillus nidulans* tolerates its deletion with only minor defects in conidiation^19,20^. In contrast, *Saccharomyces cerevisiae* shows broader dependence on H1 for chromatin regulation. There, genome-wide binding of H1 (Hho1p) becomes critical during quiescence, where it is required for chromatin compaction, telomere clustering, and chromosomal organisation^21,22^. Deletion of *HHO1* impairs quiescent cell development during the stationary phase, reducing viable quiescent cell numbers whilst enhancing non-quiescent cell survival, consistent with H1 enforcing cellular dormancy^22^. H1 similarly accumulates on chromatin during sporulation induced by nitrogen and carbon starvation, achieving approximately 10-fold nuclear compaction in mature spores^16,23^. At the other extreme, H1 deletion in *Ascobolus immersus* causes colonies to senesce after only a few divisions, underscoring an essential role in viability^24^. These divergent phenotypes suggest that H1 function has diverged substantially across fungal lineages to meet species-specific chromatin demands. However, its role across the Basidiomycota, a phylum encompassing numerous human fungal pathogens, remains entirely uncharacterised.

Here we show that the *Cryptococcus* genus uniquely encodes two paralogous linker histones, H1.51 and H1.52, arising from an ancient gene duplication absent from all other basidiomycete lineages examined. We investigated how these paralogs shape chromatin and the transcriptional landscape under host-mimicking conditions, using genomic, transcriptomic, and epigenomic profiling of knockout and complemented strains. Both paralogs have undergone functional divergence. H1.51 exerts minimal transcriptional influence under the conditions tested, whilst H1.52 is required to maintain a genome-wide chromatin programme that supports adaptation to host-relevant stress. Loss of H1.52 abolishes adaptive H3K9me2 heterochromatin island formation, is accompanied by lateral spreading of H3K4me2 into flanking genomic regions, and selectively de-represses co-marked H3K4me2/H3K9me2 loci. These chromatin changes are accompanied by rDNA copy number loss, collapse of ribosome biogenesis transcription, and a shift towards elevated metabolic activity under host-mimicking conditions in vitro. Together, these findings reveal that ancient linker histone duplication in *Cryptococcus* has produced functionally non-redundant paralogs and establish H1.52 as a master regulator of chromatin architecture and metabolic state transitions, coupling epigenomic organisation to the transcriptional reprogramming that underlies adaptation to the host environment.

## Results

### Ancient duplication generates *Cryptococcus*-specific H1 paralogs

**Fig. 1:**
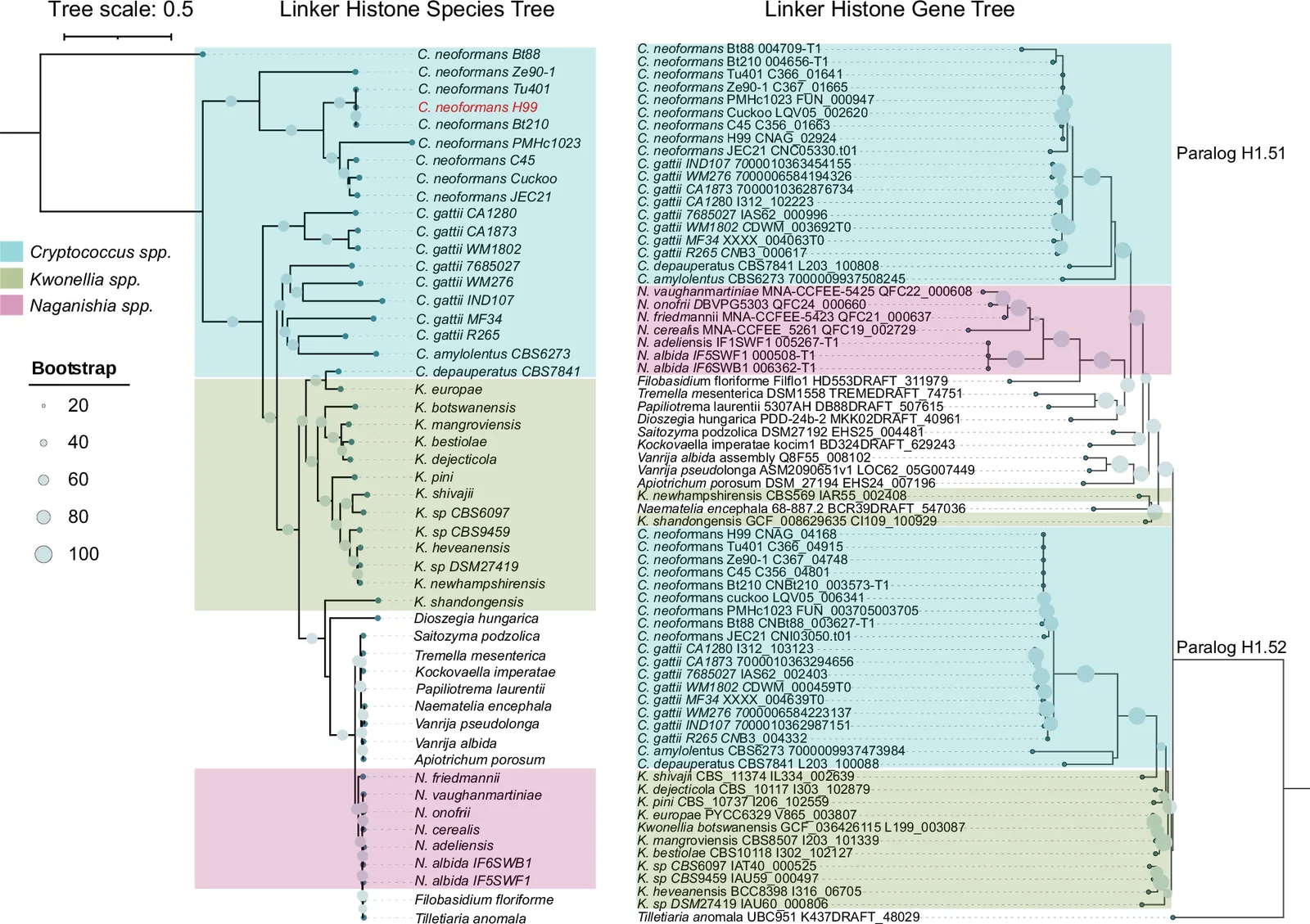
Divergence and selection in *Cryptococcus* H1 linker histone paralogs. Maximum-likelihood phylogenies of Basidiomycota species (left, 50 taxa, 210,983 amino acid sites from concatenated single-copy orthologs) and H1 linker histone genes (right, 69 sequences, 166 amino acid sites). Scale bars indicate amino acid substitutions per site for both trees. Taxa are colour coded by genus. *Cryptococcus* (teal), *Kwoniella* (green), *Naganishia* (pink). Ultrafast bootstrap support values (1000 replicates) shown as circles, sized and coloured by percentage (20–100%).

Comparative genomic analysis across 50 basidiomycete species revealed that *Cryptococcus* is the only genus with paralogous linker histones (H1.51, CNAG_02924; H1.52, CNAG_04168). Paralogs were defined as genes from the same species sharing an orthogroup, as determined by OrthoFinder using all-vs-all BLAST (E-value ≤ 1 × 10⁻⁵) and MCL clustering. This H1 duplication is conserved across all major *Cryptococcus* lineages, despite *Cryptococcus* having substantially lower genome-wide paralog content (12.7–14.7%) than the basidiomycete average (21.8%; Fig. S1). The phylogenetic topology of the H1 gene tree was incongruent with the species phylogeny. While all *Cryptococcus* species form a single clade in the species tree (Fig. 1, left), H1.51 and H1.52 segregate into two deeply divergent clades separated by other basidiomycete lineages (Fig. 1, right), consistent with an ancient duplication event that predates the diversification of *Cryptococcus*.

Following duplication, the two paralogs followed asymmetric evolutionary trajectories. H1.52 exhibited higher intra-paralog conservation (87–100%, mean = 95%) compared to H1.51 (35–100%, mean = 86%), with inter-paralog identity ranging from 27 to 83% (mean = 53%) across *Cryptococcus* isolates (Fig. S2a). Nucleotide and amino acid alignments confirmed greater sequence variability in H1.51, which exhibited more frequent deviations from the consensus compared to H1.52 (Fig. S2b, c).

To test whether this asymmetry reflects different selective pressures, we performed branch model analysis (CODEML; 227 codons, 145 parsimony-informative sites). H1.51 evolved under moderate purifying selection (ω = 0.499) whereas H1.52 remained under significantly stronger constraint (ω = 0.231; 2ΔlnL = 4.86, *p* < 0.05; Table S1). The elevated dN/dS of H1.51 could reflect either passive relaxation following duplication or active adaptive evolution. RELAX analysis distinguished between these possibilities, revealing intensified rather than relaxed selection on H1.51 (K = 7.175, *p* < 0.001; Table S1). Gene-wide testing confirmed that this intensification includes positive selection acting on H1.51 (BUSTED: LRT = 39.72, *p* = 1.18 × 10⁻⁹; Table S1), with approximately 1.5% of sites in a rate class with ω » 1.

This positive selection was concentrated on specific lineages and sites. Three H1.51 branches, all representing clinical isolates, exhibited episodic diversifying selection (aBSREL: corrected *p* < 0.001 for each; Table S2), whereas no H1.52 branch showed evidence of positive selection. At the site level, seven codons were under episodic diversifying selection (MEME: *p* < 0.05; Table S3**;** Fig. S3). In contrast, H1.52 retained ancestral residues at each of these positions. Together, these analyses indicate that H1.52 has retained the ancestral linker histone role under strong purifying selection, while H1.51 has undergone neofunctionalization through adaptive substitutions at specific residues, particularly in pathogenic lineages.

### H1 paralog C-terminal tails have different biochemical properties

**Fig. 2:**
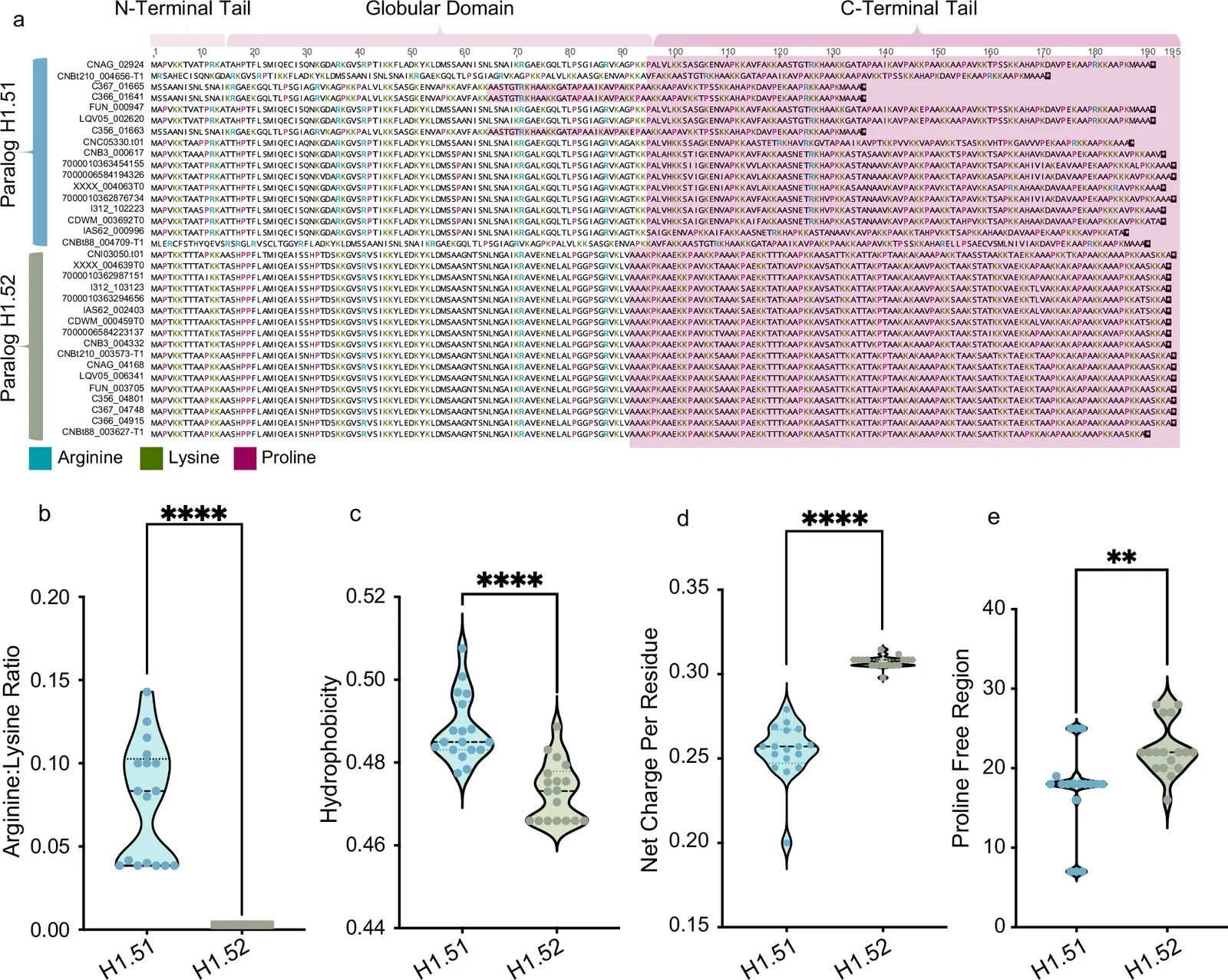
Opposing condensation strategies in H1 paralog C-terminal tails. **a** Full-length H1 peptide sequences (Paralog H1.51, *n* = 17; Paralog H1.52, *n* = 17) from *Cryptococcus* with the C-terminal tail (Pink). Arginine, Lysine and Proline residues are highlighted in teal, green and pink, respectively. **b** Arginine:lysine ratio [R/(R + K)]. Paralog H1.51 (*n* = 17) contains arginine (mean 7.7%, range 1–3 residues), while Paralog H1.52 (*n* = 17) lacks arginine entirely ***P < 0.001, Cohen’s d = 4.29. **c** Hydrophobicity (Kyte-Doolittle) is significantly enhanced in the H1.51 variant (**P < 0.001, H1.51 0.488 ± 0.008 vs H1.52 0.473 ± 0.007). **d** Net charge per residue (NCPR), calculated as (R + K + H-D-E)/length is significantly increased in H1.52 paralogs (0.307 ± 0.004) compared to H1.51 paralogs (0.254 ± 0.017) ***P < 0.001, d = 5.05. **e** Paralog H1.52 has extended proline-free stretches (22.4 ± 3.3 aa) compared to Paralog H1.51 (17.2 ± 5.6 aa). In panels b–e, H1.51 paralogs are shown in teal and H1.52 paralogs in green. Statistical comparisons by two-tailed Welch’s t-test or Mann-Whitney U test (see Methods). ***P < 0.001, d = 1.14. Significance threshold α = 0.05. Data are mean ± s.d.

To investigate potential functional specialisation between H1 paralog groups, we analysed the biochemical properties of the C-terminal intrinsically disordered tail (residues 100-terminus) across 34 paralog sequences from *C. neoformans* **(**Fig. 2a**)**. Measurable biophysical features that drive linker histone-mediated chromatin condensation include arginine content, net charge density, proline-free region length, and hydrophobicity^25^. Paralog H1.51 (*n* = 17) contains arginine at 7.7 ± 3.6% of basic residues (1–3 arginine’s per tail), whereas Paralog H1.52 (*n* = 17) lacks arginine entirely (Fig. 2b) (*p* < 0.001, Cohen’s d = 4.29). Hydrophobicity was also significantly higher in Paralog H1.51 (Fig. 2c) (0.473 ± SD; *p* < 0.0001, d = 1.92), however the difference in hydrophobicity between paralogs was modest (3%). Net charge per residue (NCPR), which measures the average net electrical charge per amino acid and is calculated as (R + K + H − D − E)/L, was 21% higher in Paralog H1.52 (NCPR = 0.307 ± 0.004) compared to Paralog H1.51 (NCPR = 0.254 ± 0.017; *p* < 0.001, d = 5.05) (Fig. 2d). Previous work has demonstrated that increased charge density independently drives robust condensation through enhanced electrostatic interactions with DNA, suggesting that Paralog H1.52 compensates for the absence of arginine by maintaining higher overall positive charge^25^. Additionally, Paralog H1.52 possesses 30% longer proline-free regions (22.4 ± 3.3 amino acids) than Paralog H1.51 (Fig. 2e) (17.2 ± 5.6 amino acids; *p* < 0.001, d = 1.14), another property that predicts enhanced condensation capacity.

### H1.52 is a master regulator of gene expression in *C. neoformans*

**Fig. 3:**
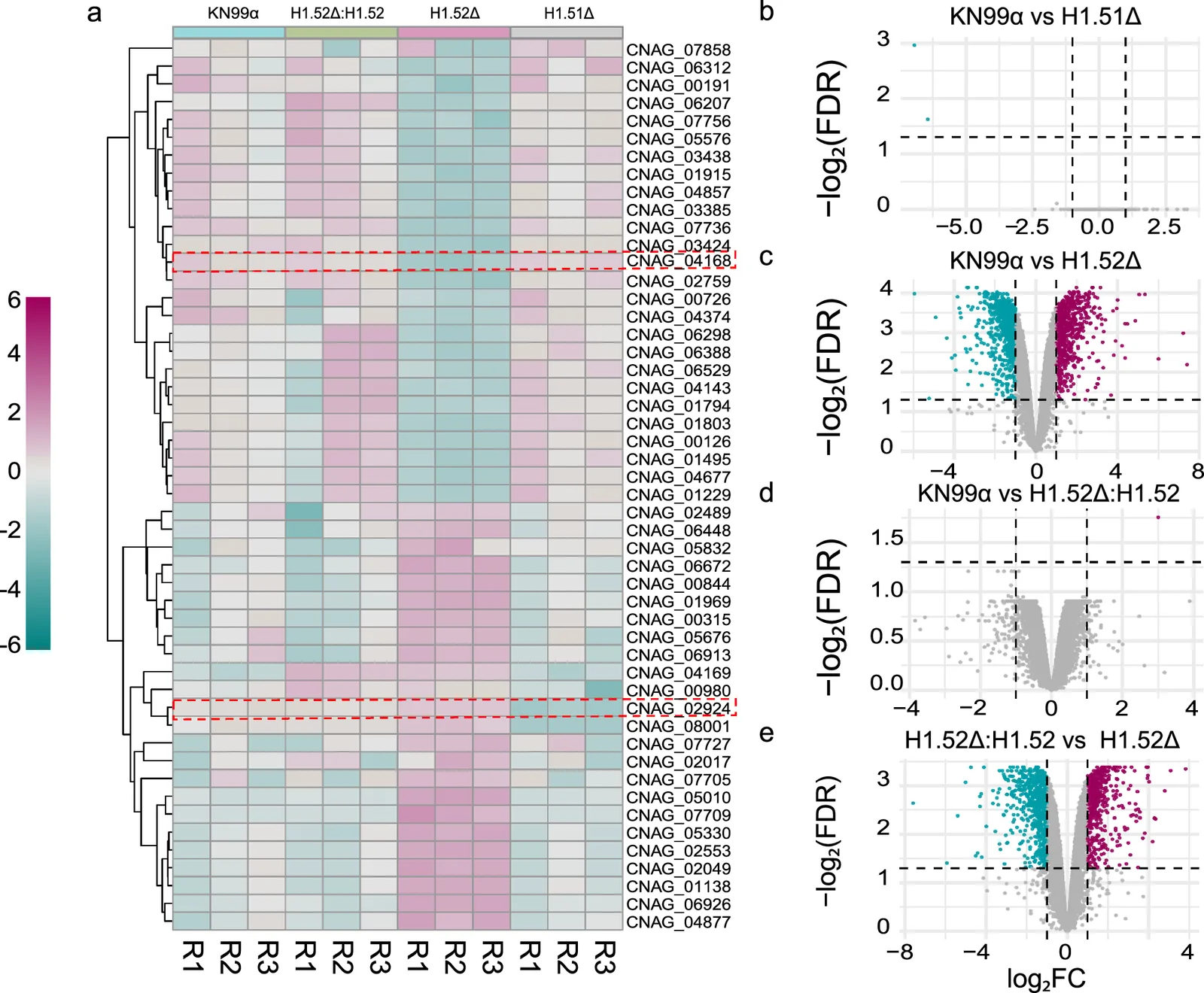
Loss of H1.52 drives transcriptome rewiring in host-relevant conditions. RNA-seq was performed on *n* = 3 independent biological replicates per strain; differential expression assessed by two-sided Wald test (DESeq2) with Benjamini-Hochberg FDR correction. **a** Heatmap showing log₂-transformed, row-centred expression values of the 50 most variable differentially expressed genes (FDR < 0.05, log₂FC > 1) across biological triplicates. Columns show KN99α Control (teal), H1.52Δ:H1.52 (green), H1.52Δ (pink), H1.51Δ (grey). Scale is teal (lowly expressed transcripts) to pink (highly expressed transcripts). **b–d** RNA-seq differential-expression volcano plots (log₂FC  >  1, FDR  <  0.05) comparing each mutant to KN99α control. **b** KN99α vs H1.51Δ with only two differentially expressed genes. **c** KN99α vs H1.52Δ with 1561 differentially expressed genes. **d** KN99α vs Complemented (H1.52Δ:H1.52) with 1 differentially expressed gene. **e** H1.52Δ:H1.52 vs H1.52Δ with 1215 differentially expressed genes. Up- and down-regulated genes are shown in pink and teal, respectively, with non-significant genes in grey.

The ∆H1.51 and ∆H1.52 knockout strains were obtained from the Madhani deletion collection (Fungal Genetics Stock Centre), and gene deletions were generated by targeted replacement with a nourseothricin resistance cassette. Complementation strains were constructed by CRISPR-mediated reintegration of the wild-type allele at the Safe Haven 4 locus. After 72 h under host mimicking conditions (PBS supplemented with 10% heat-inactivated foetal bovine serum, incubated at 37 °C in 5% CO₂), deletion of the derived linker-histone paralogue H1.51 had minimal transcriptional impact (Figs. 3a, 3b; Supplementary Data 1)^3^. Only two differentially expressed genes (DEGs) were identified: H1.51 itself (log₂FC = –6.9; FDR = 0.001) and the hypothetical ORF CNAG_08001 (log₂FC = -6.4). DNA-seq analysis revealed poor coverage across the CNAG_08001 locus (12% breadth, 9% depth), indicating this gene is likely absent or deleted from this strain. By contrast, loss of the conserved paralog H1.52 elicited a broad transcriptional rewiring with 1561 genes (23% of transcripts; 747 up-, 814 down-regulated) differentially expressed. H1.52 was the most strongly repressed transcript (log₂FC = –5.9; FDR = 1.04 × 10⁻⁴), confirming successful silencing, while the H1.51 paralog was significantly induced (log₂FC = +1.2; FDR = 9.48 × 10⁻⁴), consistent with partial functional compensation (Fig. 3c).

Genetic complementation (strain H1.52Δ:H1.52) restored the wild-type landscape. In the complemented strain, the only DEG shared between the complement and the wild-type strain (KN99α) was CNAG_04169 (holo-[acyl-carrier-protein] synthase), which lies adjacent to the original H1 locus and likely reflects local positional effects (Fig. 3d). Furthermore, H1.52 expression (CPM, TMM-normalised) is similar in the complement strain (274 ± 12) and the KN99α control strain (297 ± 15), confirming full functional rescue. Principal-component analysis demonstrated that H1.52Δ:H1.52 replicates clustered with KN99α, with PC1 and PC2 explaining 74.7% and 9.0% of total variance, respectively (Fig. S3a). Pearson correlation analysis revealed good reproducibility among biological replicates (KN99α: *r* = 0.45–0.82; H1.52Δ:H1.52: *r* = 0.44–0.75) and moderate correlation between H1.52Δ:H1.52 and KN99α samples (*r* = 0.23–0.67), confirming successful genetic complementation. H1.52Δ replicates showed exceptional reproducibility (Pearson *r* = 0.92–0.96), reflecting the strong transcriptional effect of this deletion (Fig. S3b).

One thousand, two hundred and fifteen genes (18% of transcripts; 562 up-, 653 down-regulated) were differentially expressed in H1.52Δ:H1.52 compared with H1.52Δ (Fig. 3e). H1.52 was again the most strongly induced transcript (log₂FC = +5.8, FDR = 2.5 × 10⁻⁶), confirming successful genomic reinsertion upon complementation. Together, these data demonstrate that the H1.52 paralog exerts a genome-wide influence in *C. neoformans* cells grown under host-mimicking stress, and that its loss affects nearly one quarter of the transcriptome, an effect that is fully reversible upon gene complementation.

### H1.52 deletion alters rDNA copy number and ribosome biogenesis-related transcription in *C. neoformans*

**Fig. 4:**
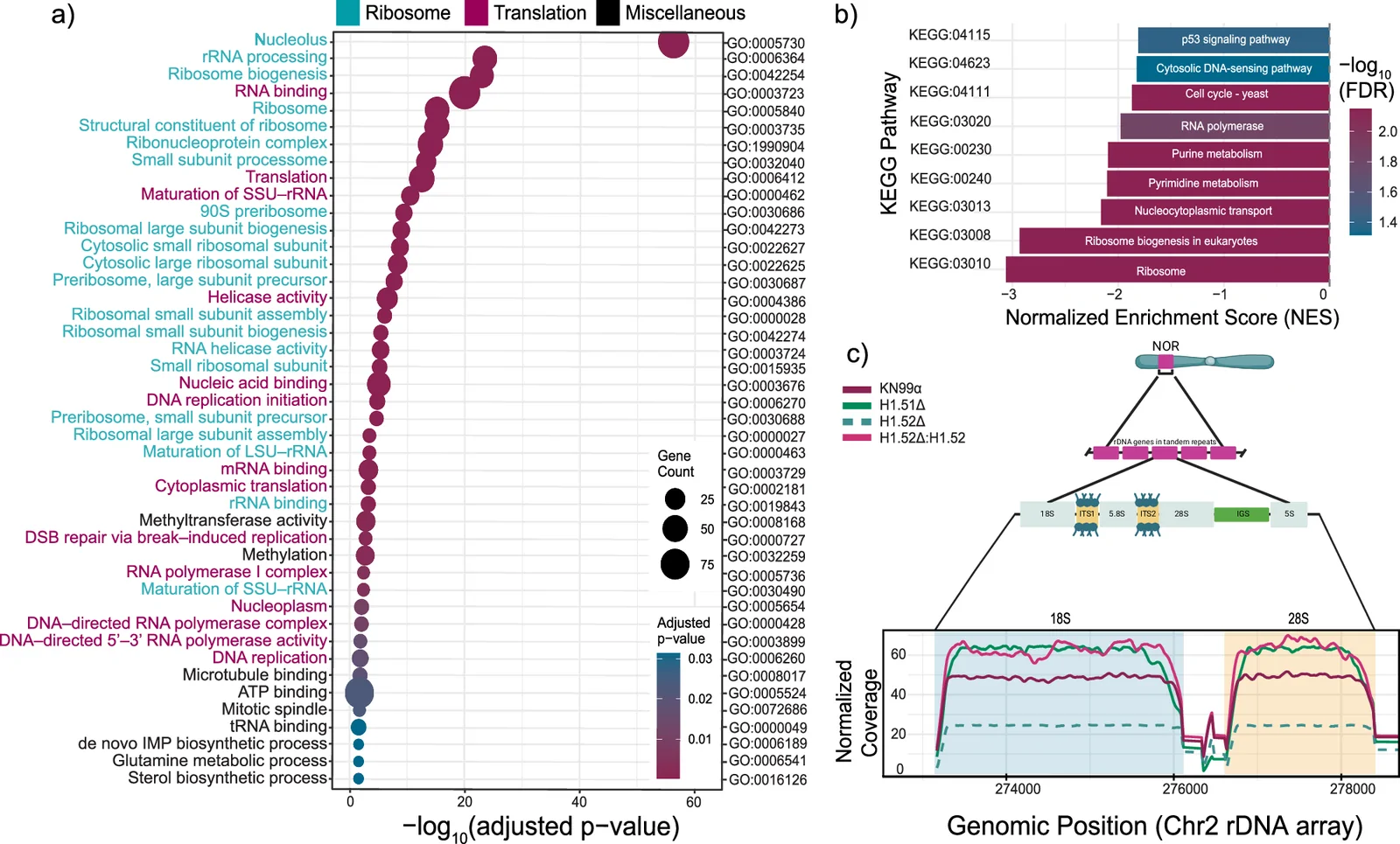
H1.52 Deletion Alters rDNA Copy Number and Ribosome Biogenesis-Related Transcription in *C. neoformans.* **a** Gene Ontology enrichment analysis of 665 genes significantly downregulated in H1.52Δ versus wild-type KN99α (FDR < 0.05, log₂FC < -1.0). Top 20 enriched GO terms ranked by adjusted *p*-value. Dot size represents gene count per category; colour intensity indicates -log₁₀(adjusted p-value). Two-sided Fisher’s exact test with Benjamini-Hochberg correction. **b** KEGG pathway enrichment analysis by Gene Set Enrichment Analysis (GSEA) of 311 pathways. Bars show normalised enrichment scores (NES) for nine significantly downregulated pathways (FDR < 0.05). Colour gradient represents -log₁₀(FDR). **c** Normalised read depth across the rDNA array after 72 h under host-mimicking conditions (pooled DNA from *n* = 3 biological replicates per strain). H1.52Δ shows a marked reduction in rDNA coverage compared to wild-type and complemented strains. Strains are coloured as follows: KN99α (purple), H1.51Δ (green), H1.52Δ (dashed teal), and H1.52Δ:H1.52 (pink).

Deletion of the H1.52 paralog led to significant downregulation of 814 genes (log_2_fold change < -1, FDR < 0.05), with 44 significant GO terms, the majority of which relate to ribosome biogenesis (blue) and translation (purple) in H1.52Δ (Fig. 4a; Supplementary Data 2). GO-terms significantly enriched in downregulated genes include ribosomal RNA processing (44 genes, FDR < 0.001), ribosome biogenesis (40 genes, FDR < 0.001) and cytosolic large- and small- ribosomal subunit processomes (16 and 24 genes) (FDR < 0.001).

Gene set enrichment analysis (GSEA) of 311 KEGG pathways revealed that nine were significantly downregulated (FDR < 0.05) in the H1.52Δ strain. Among those pathways, ribosome (KEGG:03010; Normalised Enrichment Score (NES) = -3.06) and ribosome biogenesis (KEGG:03008; NES = -2.93) showed the strongest suppression. RNA polymerase (KEGG:03020; NES = -1.98) was also downregulated, with pronounced suppression of RNA polymerase I-specific subunits (*RPA1*, *RPA2*, *RPA12*, *RPA43*, *RPA49*), directly implicating reduced rRNA synthesis as the primary driver of reduced ribosome production. Supporting this mechanism, several nucleotide biosynthesis pathways, including purine (NES = -2.10) and pyrimidine metabolism (NES = -2.11), which encode precursors for rRNA synthesis, were similarly downregulated. Nucleocytoplasmic transport (NES = -2.16) was also suppressed, indicating coordinated disruption of ribosome assembly and export pathways (Fig. 4b). Together, these analyses suggest that H1.52 may influence ribosomal biosynthesis via the nucleolus, the site of rRNA synthesis.

To determine whether transcriptional suppression of ribosome biogenesis reflected structural changes at the rDNA locus itself, we quantified rDNA copy number using DNA-seq coverage analysis. Under standard YPD culture conditions, normalised read depth across the Chromosome 2 rDNA tandem array (18S–5.8S–28S repeats) was equivalent across KN99α, H1.52Δ, and H1.52Δ:H1.52, indicating no constitutive rDNA copy number difference between genotypes (Fig. S4a, c). However, following 72 hours under host-mimicking conditions (PBS supplemented with 10% heat-inactivated foetal bovine serum, 37°C, 5% CO₂), H1.52Δ exhibited a marked ~50% reduction in rDNA coverage compared to wild-type, from 47.1 ± 6.1 to 23.7 ± 3.4 (49.6% loss) at 25S, and from 46.6 ± 7.8 to 23.3 ± 4.0 (50.0% loss) at 18S, consistent with halving of rDNA copy number (Fig. 4c, Fig. S4b, c). Neither H1.51Δ nor the H1.52Δ-complement exhibited this stress-induced depletion. Importantly, the complemented strain was generated by transforming a cryopreserved H1.52Δ stock that had never been subjected to host-mimicking conditions. Its rDNA array was therefore intact at the time of complementation, as confirmed by equivalent normalised read depth under YPD. The rDNA stability observed in the complemented strain under stress consequently reflects genuine rescue of H1.52-dependent protective function rather than an artefact of strain history. We therefore conclude that rDNA instability is a condition-dependent phenotype of H1.52 deletion, rather than a fixed genomic difference that confounds strain comparisons under standard culture. This stress-induced loss of rDNA repeats suggests increased instability at repetitive loci in the absence of H1.52, providing a mechanistic basis for the coordinated suppression of RNA polymerase I activity, nucleotide biosynthesis, and ribosome biogenesis pathways observed at the transcriptional level^26^.

### H1.52 governs H3K4me2 and H3K9me2 genomic distribution in *C. neoformans*

H1.52 governs H3K4me2 and H3K9me2 genomic distribution in *C. neoformans*

Linker histones are heterochromatin associated proteins that regulate chromatin compaction and genome organisation. Given that H1.52 deletion causes 50% rDNA loss and transcriptional collapse of nucleolar and ribosome biogenesis pathways, and that nucleolus-associated domains are enriched in H3K9me2^27^, we investigated whether H1.52 regulates genome-wide heterochromatin architecture by performing ChIP-seq mapping of H3K9me2 and H3K4me2 after 72 hours under host-mimicking conditions (Supplementary Data 3). Gene-level annotations were defined using TSS ± 1.5 kb reflecting the compact intergenic spacing of the *C. neoformans* genome. This revealed extensive H1.52 dependent chromatin remodelling. Specifically, H3K9me2 domains underwent architectural reorganisation following H1.52 deletion.

**Fig. 5:**
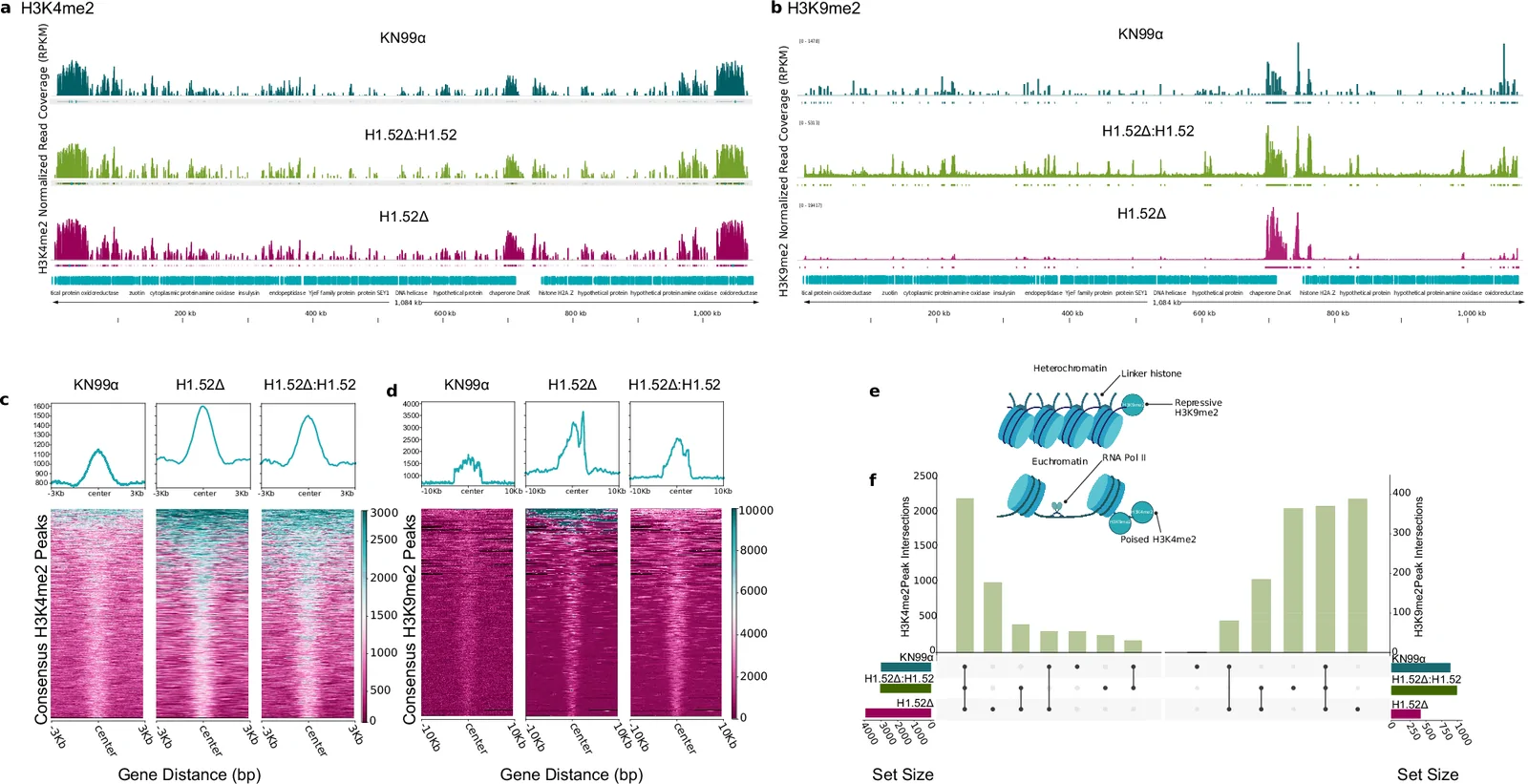
H1.52 loss remodels euchromatin and heterochromatin distribution genome wide. ChIP-seq performed with n = 2 biologically independent replicates per strain, merged using a union approach for consensus peak calling. Peaks called using MACS3 (H3K4me2: narrowPeak q < 0.05; H3K9me2: broadPeak q < 0.05, broad-cutoff 0.1); see Methods. **a**,**b** Chromosome 4 browser tracks showing log10-scaled ChIP-seq signal for H3K4me2 (**a**) and H3K9me2 (**b**) in KN99α (teal), H1.52Δ (pink) and complement H1.52Δ:H1.52 (green). **c**,**d** Genome-wide heatmaps and metagene profiles of FE-normalised ChIP-seq signal for H3K4me2 (±3 kb from TSS; **c** and H3K9me2 (±10 kb from TSS; **d**); colour intensity in the heatmaps reflects normalised read signal as indicated by the adjacent scale bars. **e** Schematic illustrat**e**s the proposed model: in the presence of the linker histone, nucleosomes (blue) are compacted into heterochromatin marked by repressive H3K9me2, whereas loss of the linker histone opens chromatin into euchromatin accessible to RNA Pol II and marked by poised H3K4me2. **f** UpSet plots showing peak intersections for H3K4me2 (left) and H3K9me2 (right) across strains. Horizontal bars (Set Size) show total peaks per strain: H3K4me2, KN99α 4,241, H1.52Δ 3,970, H1.52Δ:H1.52 3,655; H3K9me2, KN99α 1,241, H1.52Δ 506, H1.52Δ:H1.52 1,074. Filled dots indicate the strains contributing to each intersection and connected dots indicate peaks shared between strains; top bars show intersection size.

In wild-type KN99α, H3K9me2 marked 1.41 Mb (7.5% genome coverage) across 1241 peaks (median width 516 bp), encompassing promoters of 1254 genes. In the H1.52Δ mutant, total H3K9me2 coverage remained comparable at 1.79 Mb (9.5%), but exhibited substantial redistribution. Peak number decreased 2.5-fold to 506, while median peak width increased 3.7-fold to 1,937 bp (Fig. 5b, d, f). This consolidation concentrated H3K9me2 at constitutive heterochromatin regions, with concurrent loss of H3K9-marked heterochromatin islands. Gene-level analysis revealed 639 genes that lost H3K9me2 peaks in H1.52Δ, with 615 genes retaining H3K9me2 peaks in both wild-type KN99α and H1.52Δ strains (Jaccard similarity 0.44). Complementation rescued the dispersed H3K9me2 architecture. The H1.52Δ:H1.52 strain restored H3K9me2 coverage to 2.74 Mb (14.5%) across 1074 peaks marking 1903 genes, consistent with restoration of H3K9-marked heterochromatin islands (Fig. 5b, d, f).

Loss of heterochromatic regions was accompanied by an increase in genomic coverage of H3K4me2 in H1.52Δ. In wild-type KN99α cells, H3K4me2 occupied 3.47 Mb (18.4% genome coverage) distributed across 4241 peaks with a median width of 369 bp, overlaying promoter regions of 3341 genes (Fig. 5a, c, f). Deletion of H1.52 induced pronounced euchromatin expansion, with H3K4me2 coverage increasing 1.54-fold to 5.36 Mb (28.4% of the genome). This expansion occurred through peak broadening rather than de novo peak formation as peak number remained comparable at 3970 while median peak width increased 2.1-fold to 776 bp encompassing 4560 genes in H1.52Δ. Genetic complementation only partially restored wild-type chromatin organisation. The H1.52Δ:H1.52 complemented strain exhibited H3K4me2 coverage of 4.04 Mb (21.4%) across 3655 peaks marking 4141 genes, exhibiting intermediate levels between wild-type and knockout strains (Fig. 5a, c, f). Gene-level differential analysis identified 1437 loci that gained H3K4me2 in H1.52Δ, with 3123 genes retaining H3K4me2 marks across both wild-type and knockout strains (Jaccard similarity index 0.65).

To determine whether H1.52 constrains H3K4me2 domain boundaries, we quantified H3K4me2 signal within inter-peak intervals (gaps <500 bp between adjacent KN99α peaks). H1.52Δ showed 18% elevated inter-peak signal relative to wildtype (p = 6.5 × 10⁻³⁴, Wilcoxon rank-sum; Fig. S6a), with complementation partially rescuing this effect (33% reduction in signal gain; Fig. S6a). Gap filling was distance-dependent: >95% of gaps <50 bp were filled in H1.52Δ compared to <5% of gaps >3 kb (Fig. S6b), consistent with lateral encroachment from existing peaks rather than de novo deposition. Meta-profile analysis centred on KN99α peak midpoints confirmed that the H3K4me2 increase was concentrated in flanking regions while summit signal remained relatively stable across strains (Fig. S6c). Genome-wide, 40% of H1.52Δ peaks showed evidence of spreading with 22% broadened beyond KN99α boundaries and 18% represented mergers of adjacent wildtype peaks consuming 1839 KN99α peaks (43.4% of total; Fig. S6d). Merged peaks were significantly wider than all other classes (median 2137 bp versus 843 bp for retained peaks, p < 10⁻¹⁰⁰; Fig. S6e). These data demonstrate that H1.52 maintains discrete H3K4me2 domain architecture by preventing lateral spreading into flanking chromatin (Fig. S6f).

### H1.52 maintains transcriptional repression at H3K4me2/H3K9me2 co-marked loci in *C. neoformans*

**Fig. 6:**
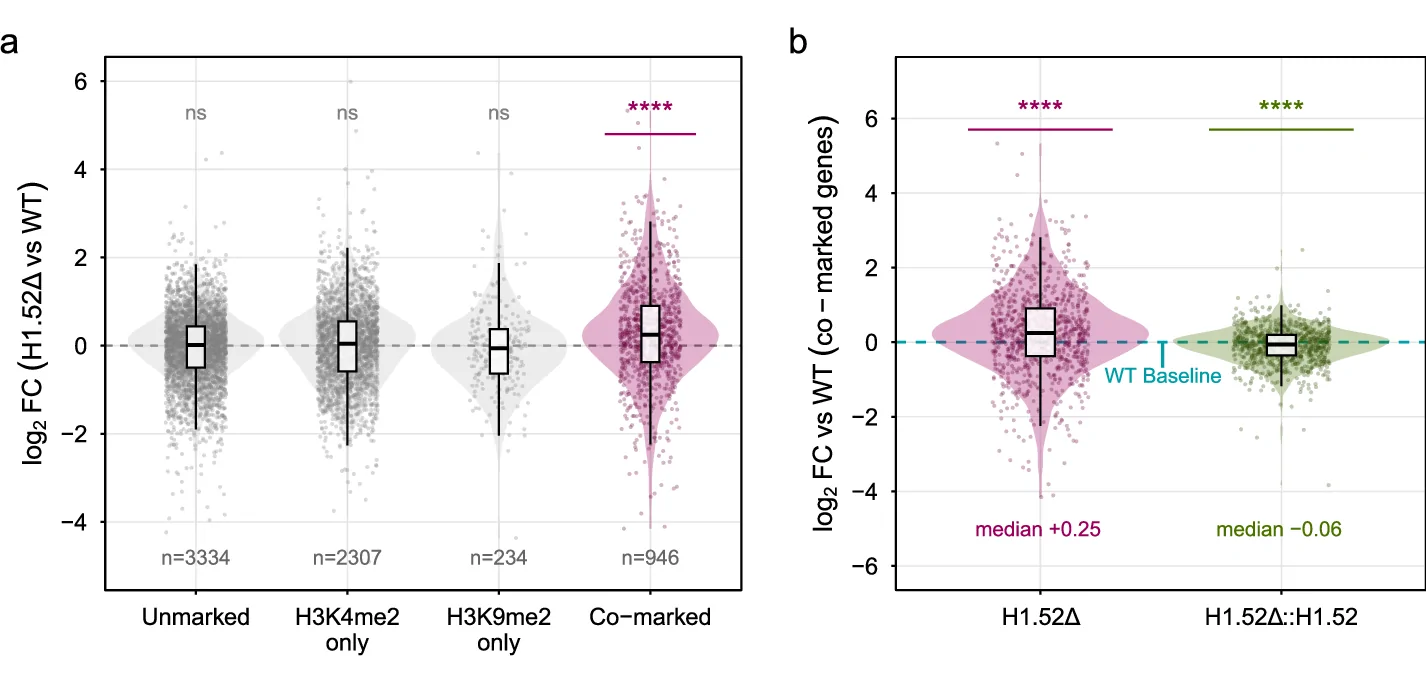
Loss of histone H1.52 causes selective upregulation of co-marked genes. **a** Expression changes (log^2^ fold change, H1.52Δ versus wild-type KN99α) for genes classified by chromatin state: unmarked (*n* = 3334), H3K4me2-only (n = 2,307), H3K9me2-only (*n* = 234), and co-marked (H3K4me2+H3K9me2; *n* = 946). Violin plots show distribution with individual data points; boxplots indicate median and interquartile range. Dashed line indicates no change relative to wild-type. Significance assessed by one-sample two-sided Wilcoxon signed-rank test against a median of zero. Only co-marked genes show significant upregulation (median log₂FC = +0.25; p = 3.2×10^-14^). ns, not significant; ****p < 0.0001. **b** Expression of co-marked genes (*n* = 946) in H1.52Δ and H1.51Δ:H1.51 strains relative to wild-type KN99α. Dashed line indicates wild-type baseline. Co-marked genes are significantly upregulated in H1.52Δ (median log₂FC = +0.25; *p* = 3.2×10^-14^) and fall significantly below wild-type baseline upon complementation (median log₂FC = −0.06; *p* = 6.9×10^-7^ versus wild-type), consistent with H1.52 dependant repression of co-marked loci. ****p < 0.0001.

To determine whether the observed chromatin changes drive transcriptional dysregulation, we intersected H3K4me2 and H3K9me2 peak data with RNA-seq expression profiles. In KN99α, 946 genes (13.9% of the genome) carry both H3K4me2 and H3K9me2 simultaneously at their promoters. This co-marked chromatin state may be indicative of transcriptional poising. Co-marked genes were 1.64-fold more likely to be significantly upregulated in H1.52Δ than genes genome-wide (OR = 1.64, 95% CI [1.42–1.89], *p* = 1.5×10^-11^). This upregulation was exclusive to the co-marked class, as genes marked solely with H3K4me2 showed no significant expression change (*p* = 0.65), nor did genes marked solely by H3K9me2 (*p* = 0.14), whilst co-marked genes were systematically upregulated (median logFC +0.25, *p* = 3.2×10⁻¹⁴;) (Fig. 6a). Complementation returned co-marked gene expression towards wild-type levels (median logFC −0.057, *p* = 6.9×10^-7^), demonstrating that H1.52 is both necessary and sufficient to maintain transcriptional repression specifically at co-marked loci (Fig. 6b).

Functionally, upregulated co-marked genes were enriched for transmembrane transport (OR = 2.67, *p* = 2.7×10^-5^), redox and iron acquisition (OR = 2.06, *p* = 0.001). Together, these data indicate that H1.52 maintains transcriptional repression at a specific class of poised loci, with its loss causing inappropriate de-repression of iron acquisition, oxidoreductase, and transmembrane permease programmes.

### H1.52 restricts metabolic activity under host-mimicking conditions

**Fig. 7:**
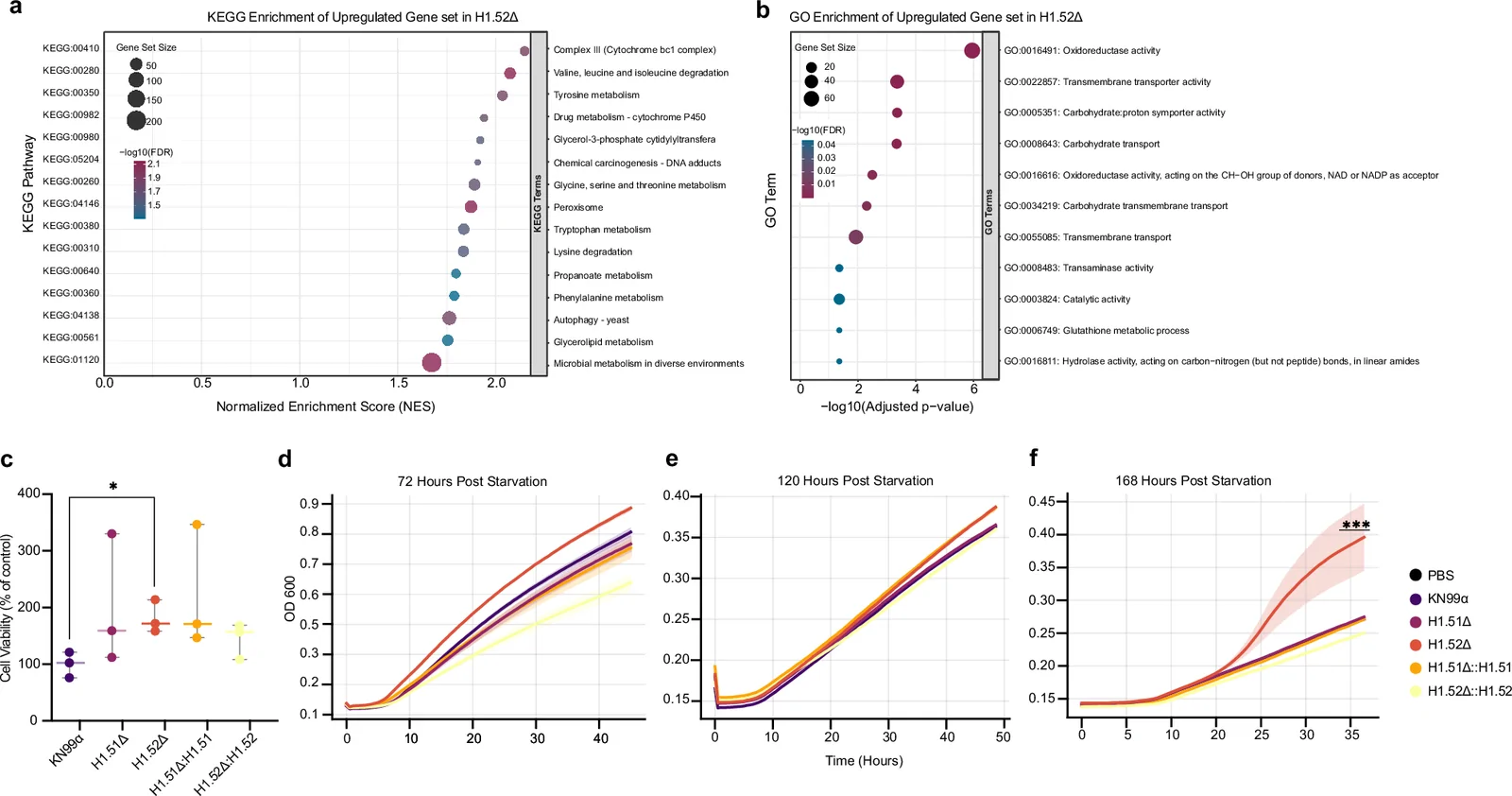
H1.52 restricts metabolic activity during prolonged host mimicking stress. **a** KEGG pathway enrichment analysis of genes significantly upregulated in H1.52Δ (*n* = 747). Bars show normalised enrichment scores (NES) for the top 15 enriched pathways (FDR < 0.05); colour gradient indicates −log₁₀(FDR). **b** Gene Ontology enrichment of the same upregulated gene set. Dot size indicates gene count; dot colour indicates −log₁₀(FDR). Only terms with FDR < 0.05 are shown. **c** XTT metabolic activity assay performed at 72 h in PBS + 10% FBS (*n* = 3 independent biological replicates, four technical replicates each). H1.52Δ exhibits significantly elevated metabolic activity (1.81-fold relative to KN99α; unpaired two-tailed t-test, *p* = 0.018), while other strains show activity comparable to wild-type. Data represent mean ± SD. **d–f** Growth curve analysis following nutrient re-addition after **d** 3 days, **e** 5 days, and **f** 7 days in PBS + 10% FBS. Lines represent mean OD_600_ ± SEM from technical duplicate measurements of three biological replicates. H1.52Δ (red) shows enhanced recovery with higher maximum OD_600_ and faster doubling times compared to KN99α (purple) across all timepoints. H1.52Δ:H1.52 (yellow) shows the slowest recovery with reduced maximum OD_600_ and reduced doubling times. H1.51Δ (pink) and H1.51Δ:H1.51 (orange) show minimal deviation from KN99α. Statistical comparisons performed using two-sided Mann-Whitney U tests with Bonferroni correction for multiple testing (***p < 0.0125, **FDR-adjusted *p* < 0.05).

Gene Ontology enrichment analysis of upregulated genes in H1.52Δ identified significant enrichment for oxidoreductase activity (GO:0016491, 66 genes, FDR = 1.1×10^-6^), transmembrane transporter activity (GO:0022857, 44 genes, *p* = 4.4×10^-4^), and carbohydrate transport (GO:0008643, 16 genes, FDR = 4.6×10^-4^) (Fig. 7a; Supplementary Data 2). Complementary gene set enrichment analysis of 311 KEGG pathways identified 15 significantly upregulated pathways in H1.52Δ (FDR < 0.05; Supplementary Data 2). The enriched pathways indicate activation of alternative metabolic routes, encompassing multiple amino acid metabolic pathways, including valine, leucine, isoleucine, lysine, tryptophan, phenylalanine, tyrosine, glycine/serine/threonine and glycerolipid metabolism. Additionally, mitochondrial energy metabolism via Complex III (cytochrome bc1 complex) was significantly enriched, suggesting enhanced respiratory activity (Fig. 7b; Supplementary Data 2). Notably, H1.52Δ cells also exhibited activation of multiple catabolic programmes. Analysis of peroxisomal β-oxidation machinery revealed widespread upregulation. Seven peroxisome genes were significantly upregulated (*PEX2*, 3, 6, 7, 13, 16 and 19; logFC range 0.91–1.59, all FDR < 0.015). Fatty acid oxidation enzymes were similarly elevated, including acyl-CoA oxidase (logFC = 1.85, FDR = 0.007), isovaleryl-CoA dehydrogenase (logFC = 0.98, FDR = 0.018), and multiple acyl-CoA dehydrogenases and hydratases (logFC range: 1.63–2.07, all FDR < 0.005). The peroxisomal long-chain fatty acid import protein ALDP showed increased expression (logFC = 1.04, FDR = 0.002), along with carnitine acetyltransferase (logFC = 0.97, FDR = 0.036), indicating enhanced lipid and amino acid catabolism.

Consistent with the transcriptional profile, XTT assays performed after 72 h revealed significantly elevated XTT reduction in H1.52Δ cells (1.81-fold relative to KN99α; *p* < 0.05), indicating increased overall metabolic activity in the H1.52 knockout strain (Fig. 7c). This metabolic enhancement translated into enhanced growth compared to KN99α following a shift to rich media after prolonged incubation in host-relevant conditions, across all durations (post 3-, 5- and 7- days culture). The H1.52Δ strain demonstrated faster doubling times (3 days: 11.8 ± 0.4 h; 5 days: 34.3 ± 1.0 h) and achieved higher maximum OD_600_ (3 days: 0.796 ± 0.028, FDR-adjusted *p* = 0.042; 5 days: 0.395 ± 0.005, Bonferroni-adjusted *p* < 0.001; 7 days: 0.426 ± 0.208, Bonferroni-adjusted *p* < 0.001). However, at 7 days, H1.52Δ displayed marked phenotypic heterogeneity, with one replicate achieving substantially higher growth (OD_600_ 0.707) while others remained closer to KN99α levels (0.279-0.292). Conversely, H1.52Δ:H1.52 showed slower recovery compared to KN99α with significantly slower doubling times (3 days: 25.5 ± 2.3 h; 7 days: 35.0 ± 1.9 h) and reduced maximum OD_600_ (3 days: 0.569 ± 0.099; 7 days: 0.257 ± 0.011; Bonferroni-adjusted p < 0.001 for all comparisons), suggesting that H1.52 normally restrains metabolic activity during prolonged culture in host-mimicking conditions. H1.51Δ exhibited minimal phenotypic effects compared to KN99α. H1.51Δ:H1.51 showed enhanced recovery at 5 days (OD_600_ 0.393 ± 0.011, Bonferroni-adjusted *p* < 0.001) and 7 days (OD_600_ 0.279 ± 0.003, FDR-adjusted *p* = 0.028), suggesting functionally distinct roles for the two H1 isoforms in the host mimicking culture conditions (Fig. 7d, e).

### *C. neoformans* H1 paralogs are dispensable for virulence in *Galleria mellonella*

**Fig. 8:**
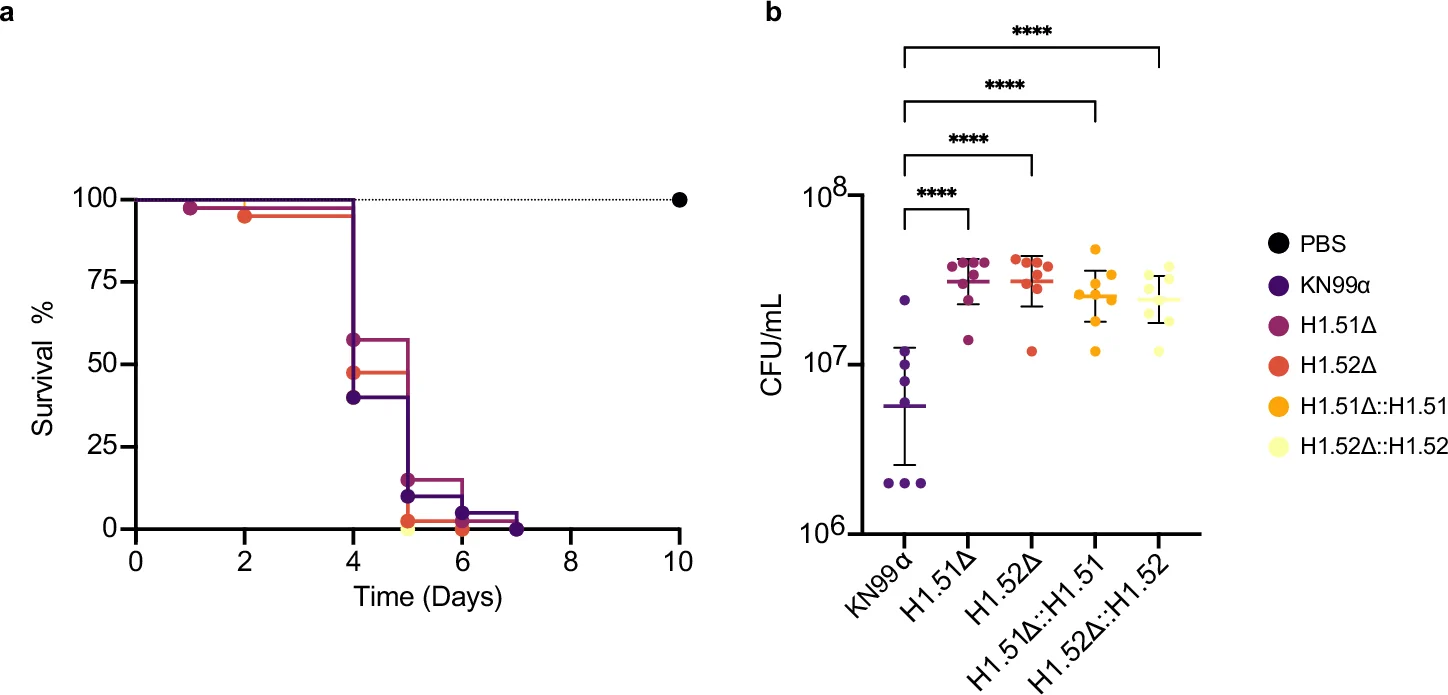
H1 paralogs are dispensable for virulence in *Galleria mellonella.* **a** Survival curves of *Galleria mellonella* larvae infected with KN99α (purple), H1.51Δ (pink), H1.52Δ (red), H1.51Δ:H1.51 (orange), and H1.52Δ:H1.52 (yellow) strains. Larvae were infected with 10^4^ cells and incubated at 37°C. Survival was monitored daily for 10 days. Lines represent Kaplan-Meier survival estimates from n = 40 larvae per strain across two independent experiments. **b** CFU enumeration from dead larvae at time of death (*n* = 8 per strain). H1.51Δ and H1.52Δ exhibited ~5-fold higher fungal burden than wild-type KN99α (3.1 × 10^7^ vs. 5.7 × 10^6^ CFU/ml; *p* < 0.0001). Complemented strains showed partial restoration but remained significantly elevated above wild-type (2.4-2.5 × 10^7^ CFU/ml; *p* < 0.0001). Bars indicate mean ± SEM. Two-sided one-way ANOVA on log₁₀-transformed data (F₄,₃₅ = 13.44, *p* < 0.0001) with Tukey’s HSD post-hoc test. ***p < *0.0001; ns, not significant*.

To assess whether H1 paralog loss affects pathogenic capacity, we evaluated virulence of H1.51Δ, H1.52Δ, and complemented strains using the *Galleria mellonella* infection model. Despite the pronounced transcriptional, chromatin, and metabolic changes observed in H1.52Δ cells under host-relevant conditions, all H99-derived strains (KN99α, H1.51Δ, H1.52Δ, H1.51Δ:H1.51, H1.52Δ:H1.52) exhibited indistinguishable virulence profiles, achieving 100% larval mortality by day 7 post-infection (Fig. 8a). This indicates that neither H1.51 nor H1.52 are individually required for pathogenesis in this invertebrate infection model.

CFU enumeration at 72 hours post-infection revealed significantly elevated fungal burden in H1-deficient strains (Fig. 8b). Both H1.51Δ and H1.52Δ showed ~5-fold higher CFU compared to wild-type KN99α (3.1 × 10⁷ vs. 5.7 × 10⁶ CFU/ml; *p* < 0.0001). Complemented strains exhibited partial restoration; however, CFU counts remained significantly elevated above wild-type levels (2.4-2.5 × 10⁷ CFU/ml; *p* < 0.0001). These data indicate that H1 paralogs restrict fungal proliferation in vivo without affecting mortality kinetics in this infection model.

## Discussion

Among 50 surveyed basidiomycete species, *Cryptococcus* represents the only lineage identified with duplicated linker histone paralogs arising from an ancestral gene duplication event. The resulting paralogs H1.51 and H1.52 diverged under opposing selective regimes. H1.51 evolved under relaxed constraint with episodic positive selection detected in clinical isolates from HIV-associated CNS infections, whereas H1.52 evolved under sustained purifying selection. This evolutionary bifurcation indicates neofunctionalization, with H1.52 preserving core chromatin organisation while H1.51 may have acquired specialised and potentially pathoadaptive capabilities, as evidenced by elevated H1.51 protein abundance in virulent (KN99α) versus latent (UgCI223) strains, and its transcriptional upregulation in *Cryptococcus*-infected non-human primates^28,29^.

Consistent with evolutionary divergence, H1 paralogs exhibit dramatically different transcriptional roles. Under host mimicking conditions, deletion of H1.51 caused minimal transcriptomic perturbation, with only two differentially expressed genes, including H1.51 itself. However, a previous study reported that H1.51 deletion results in a growth defect specifically in response to methylglyoxal (MGO)^30^. MGO is a reactive glycolytic byproduct that forms stable hydroimidazolone adducts on arginine and lysine residues, perturbing histone post-translational modifications and nucleosome stability^31^. Comparative sequence analysis revealed that H1.51 uniquely retains an arginine-bearing C-terminal tail across *Cryptococcus* lineages, whereas H1.52 lacks arginine entirely and contains solely lysine-based basic tracts. This biochemical divergence has been demonstrated to have direct functional implications for DNA binding, as while arginine’s guanidinium side chains can engage DNA through bidentate hydrogen bonds and π-stacking to react rapidly with MGO, lysine modifications (e.g., Nε-carboxyethyl-lysine) form more slowly and are less disruptive than arginine-derived products such as MG-H1^25,32,33^. When exposed to MGO H1.51’s guanidinium-rich tail may preferentially react with methylglyoxal, diverting carbonyl reactivity away from core histones. In H1.51Δ cells, loss of this protective tail may expose nucleosomal arginines to MGO attack, causing chromatin destabilisation and stress-specific growth defects. This function is particularly relevant to pathogenesis, as activated macrophages upregulate glycolysis during infection, creating methylglyoxal-rich intracellular environments, and MGO levels are elevated in people living with HIV^34,35^. Correspondingly, H1.51 shows 4.1-fold induction when *C. neoformans* is internalised within macrophages and is strongly upregulated in cerebrospinal fluid from HIV-infected patients with cryptococcal meningitis, while H1.52 remains constitutively expressed^36,37^. These observations indicate that H1.51 may confer protection against host-induced carbonyl-mediated histone damage through its chemically reactive arginine tail, an adaptive specialisation that may underlie evolutionary retention of both paralogs in *Cryptococcus*.

Unlike H1.51 deletion, H1.52Δ triggered extensive transcriptional dysregulation affecting >1500 genes, with downregulated genes enriched for nucleolar, ribosome biogenesis and RNA polymerase-associated terms. The tethering of heterochromatin to nuclear scaffold structures such as the nucleolus is central to stabilising genome architecture^38^, and linker histones have been implicated in nucleolar chromatin organisation^39^. Specific H1 variants accumulate in the nucleolus and interact with a broad spectrum of nucleolar and ribosome-associated proteins^40^. Consistent with this, H1 variants localise both to repressive nucleolus-associated domains (NADs) at the nucleolar periphery and to nucleolar organiser regions (NORs) that house the rDNA array^40,41^. In *S. cerevisiae*, H1 is enriched at the non-transcribed spacer (NTS) regions of rDNA, which regulate recombination and rRNA output, while H1 depletion in *Drosophila* leads to elevated γH2Av and R-loop accumulation at rDNA spacers, indicating a conserved role for H1 in safeguarding rDNA stability^42,43^. The rDNA locus itself is intrinsically fragile and highly susceptible to R-loop damage, undergoing repeat loss when persistent R-loops interfere with double-strand break repair^44,45^. During transcriptional stress, damaged rDNA chromatin condenses and is excluded from the active nucleolar interior (FCs/DFCs), forming peripheral caps enriched for γH2AX, linker histones, and other repressive chromatin features^46^. Mammalian H1 accumulates in these nucleolar caps^41^, underscoring its role as a stabiliser of nucleolar chromatin under replication and transcriptional stress. In line with this protective function, loss of H1.52 caused ~50% reduction in rDNA copy number, whereas H1.51Δ and complemented strains retained or expanded their rDNA repeat content when grown under host-mimicking conditions. Together, these observations position H1.52 as a key stabiliser of nucleolar chromatin in *C. neoformans* during host-relevant stress.

Functionally, H1.52Δ cells displayed enhanced mitochondrial dehydrogenase activity and faster recovery upon reintroduction to rich media after extended culture (3–7 days) in host-mimicking conditions (PBS + 10% FBS, 37 °C, 5% CO₂). In agreement, upregulated genes in H1.52Δ cells were enriched for catabolic programmes including amino acid degradation, peroxisomal β-oxidation, and autophagy. The metabolic consequences of rDNA reduction offer a plausible mechanism. Ribosome biogenesis is a major energetic sink in *S. cerevisiae*, with rRNA synthesis accounting for >60% of total transcription^47^. In *C. elegans*, reducing Pol I activity increases cellular ATP by ~1.5-fold and preserves mitochondrial function^48^. Thus, reduced rDNA copy number, curtailed Pol I transcription and suppression of core ribosome biogenesis transcription in H1.52Δ cells may preserve energy, supporting sustained catabolic metabolism and mitochondrial activity relative to the wild-type during host-induced stress. Notably, similar relationships between linker histone loss, energy expenditure, and recovery are emerging in other eukaryotes. In *C. elegans*, downregulation of the H1 ortholog HIL-1 is required for optimal recovery from prolonged fasting, as knockdown of *Hil-1* during refeeding enhances regrowth, muscle restoration and motility^11^. Whilst H1.2 loss in mammalian adipocytes promotes the trans differentiation of white to brown adipocytes, which are highly catabolic and dissipate energy through thermogenesis^49^.

The fitness consequences of H1.52 loss are infection model dependent, with pooled competition screens revealing that H1.52Δ had a greater than two-fold reduction in fitness during mouse infections; however, in this study, neither H1.51Δ nor H1.52Δ showed impaired virulence in *G. mellonella*^50^. The preserved *Galleria* virulence may reflect metabolic adaptation, as constitutive derepression of lipid β-oxidation and catabolic pathways in H1.52Δ cells could enhance survival in lipid-rich insect haemolymph^30^. Notably, in *Nakaseomyces glabratus*, which encodes a single H1 gene, H1 deletion reduces kidney colonisation and impairs biofilm formation, supporting a role for linker histones in modulating pathogenic fitness in fungi^51^. Therefore, it is plausible that the modest virulence attenuation observed in single deletion strains may also reflect partial functional compensation between H1.51 and H1.52, given their paralogous relationship. A double knockout may be required to fully reveal the contribution of linker histones to *C. neoformans* pathogenicity.

ChIP-seq analysis revealed striking chromatin remodelling defects in H1.52Δ cells. H3K9-marked heterochromatin island formation at growth-related genes occurs in *S. pombe* during nitrogen starvation and cold stress^52,53^. Similarly, we observed H3K9-marked heterochromatin island formation in wild-type *C. neoformans* during host-relevant physiological stress. However, H1.52Δ cells failed to establish many of these H3K9me2-marked islands. This failure reflects coordinated dysregulation across multiple heterochromatin pathways. Expression of the sole H3K9 methyltransferase Clr4 (CNAG_03159) was significantly reduced in H1.52Δ cells, as was the histone deacetylase Sir2 (CNAG_04866), which facilitates H3K9 methylation by removing acetylation at H3K9^54–56^. The reduced expression of Sir2 in H1.52Δ may reflect disruption of a conserved H1–sirtuin regulatory axis as in mammalian cells, H1 depletion similarly disrupts SIRT1 chromatin association and abolishes SIRT1-dependent H3K9me2 formation^17^. Conversely, H1.52Δ cells had upregulated expression of genes that code for proteins that antagonise heterochromatin formation, such as the H3K9 demethylase Epe1 (CNAG_03533) and anti-silencing acetyltransferase Mst2 (CNAG_03583), whose combined loss drives ectopic heterochromatin spreading and de novo island formation in *S. pombe*^57,58^. Additionally, H1.52Δ cells exhibited significantly reduced expression of RNAi components (Ago1, Dcr1), which are required for H3K9me2 island establishment at ribosomal, metabolic, and cell-cycle genes during quiescence entry in *S. pombe*^52^. Whether these phenotypes arise from the direct structural loss of H1.52, or cascade indirectly through transcriptional dysregulation of effectors such as Clr4 and the RNAi machinery remains an open question.

Whilst H3K9me2 loss is explicable through failure of methyltransferase recruitment, the accompanying lateral spreading of H3K4me2 cannot be attributed to altered enzymatic activity, as the sole H3K4 methyltransferase Set1 (CNAG_01243) and two core COMPASS structural subunits, Swd1 (CNAG_01828) and Swd3 (CNAG_01013), were modestly but significantly downregulated in H1.52Δ cells^59^. Genome-wide H3K4me2 domain broadening, despite coordinate downregulation of both the catalytic and structural components of COMPASS argues strongly against a writer-driven mechanism. Instead, H1.52 may itself act as a structural barrier to COMPASS access, consistent with the demonstrated ability of linker histones to directly suppress H3K4 methyltransferase activity on chromatin substrates in vitro and to restrict H3K4me2 deposition at specific loci in vivo^60,61^. In this model, H1.52 loss permits COMPASS access to inter-peak nucleosomes ordinarily compacted beyond reach, producing the lateral spreading pattern observed in the ChIP-seq data.

The selective de-repression of co-marked H3K4me2/H3K9me2 loci upon H1.52 loss is notable in the context of bivalent chromatin. In metazoan stem cells, canonical bivalency pairs H3K4me3 with PRC2-deposited H3K27me3 to hold developmental genes in a transcriptionally poised but repressed state^62,63^. H3K9 methylation can functionally substitute for H3K27me3 as the repressive partner in bivalent states in both lineage-committed and extraembryonic contexts^64,65^. H3K4me2 functions as an epigenetic poising mark in *S. cerevisiae*, persisting at previously active loci to facilitate faster reactivation^66^. Whilst *C. neoformans* encodes a functional PRC2 complex depositing H3K27me3 at subtelomeric domains, no bivalent chromatin has been reported in any basidiomycete fungus^67^. Although co-marked genes were enriched at subtelomeric regions, subtelomeric location alone did not independently predict transcriptional upregulation in H1.52Δ, instead, co-marked status was the independent predictor, indicating that the functional consequence of H1.52 loss is determined by chromatin state rather than chromosomal position. The coincidence of H3K4me2 and H3K9me2 at these loci is therefore consistent with a previously undescribed, PRC2-independent poising mechanism in which H3K9me2 substitutes for H3K27me3, with H1.52 acting as the structural determinant in maintaining repression at co-marked sites. We note that bulk ChIP-seq cannot formally distinguish true co-marking from population-level heterogeneity, and sequential ChIP approaches would be required to resolve this; nevertheless, the mark-exclusive transcriptional response and its rescue by complementation argue that co-marked loci constitute a genuine H1.52-dependent regulatory class, irrespective of whether the underlying chromatin architecture reflects bivalency within individual cells or heterogeneity across the population.

This study provides genome-wide chromatin profiling of *C. neoformans* under host-mimicking physiological stress, establishing H1.52 as a regulator of chromatin architecture directly relevant to pathogen survival^68,69^. Failure to establish H1.52-dependent chromatin remodelling has measurable physiological consequences. H1.52Δ cells exhibit elevated mitochondrial dehydrogenase activity and accelerated recovery from prolonged host stress, consistent with a chromatin landscape in which loss of H3K9me2-marked islands and lateral spreading of H3K4me2 maintains a transcriptionally poised state that facilitates rapid metabolic reactivation^66^. Transcriptional de-repression is chromatin state dependent rather than genome-wide, restricted to loci co-marked by both H3K4me2 and H3K9me2, consistent with H1.52 maintaining repression at poised loci analogous to non-canonical bivalent chromatin in metazoan lineage-committed cells^64,65^. The parallel with facultative H3K9me2 island formation during stress and quiescence entry across fungal lineages suggests this coupling of chromatin state to metabolic output may reflect a broadly conserved adaptive mechanism^52,53^. Identifying the signals that license this remodelling, resolving the combined contribution of both paralogs to host adaptation, and determining whether the fungal-specific features of this system represent a tractable vulnerability in a WHO-critical priority pathogen responsible for over 112,000 deaths annually are priorities that follow directly from this work^70^.

## Methods

### Strains and culture conditions

*C. neoformans* strains KN99α, H1.51∆, H1.52∆, H1.51∆:H1.51, and H1.52∆:H1.52 were used in this study. KN99α is a congenic MATα strain derived through repeated backcrossing with the clinical isolate H99, originally isolated in 1978 from a patient with Hodgkin’s lymphoma^71,72^. Deletion mutants H1.51∆ (CNAG_02924::NAT) and H1.52∆ (CNAG_04168::NAT) belong to the 2016 and 2015 Madhani libraries and were obtained from the Fungal Genetics Stock Centre^73^. Experimental strains were streaked from frozen glycerol stocks (-80 °C) onto YPD agar (1% yeast extract, 2% peptone, 2% glucose, 2% agar) and incubated at 30 °C for 48 hours. Single colonies were inoculated into 5 mL Yeast Nitrogen Base medium without amino acids supplemented with 2% glucose and cultured overnight at 30 °C with shaking (180 rpm). Overnight cultures were diluted to OD_600_ = 0.001 in PBS containing 10% heat-inactivated FBS (Gibco 10500-064) and incubated for 72 h at 37 °C in 5% CO_2_ to replicate host mimicking conditions^3^.

### Gene knockout and complementation

To complement H1.51Δ and H1.52Δ deletion strains, a wild-type copy of H1.51 (CNAG_02924) or H1.52 (CNAG_04168) was reintroduced to the Safe Heaven 4 (SH4) locus of the deletion strains using CRISPR^74^. The CAS9 cassette was amplified from the plasmid pMH21using ExTaq DNA polymerase (Takara Bio) with Primers M13_Fw and M13_Rv. The SH4_sgRNA cassette was constructed by PCR with primers GCn_002 and GCn_003, to fuse the DNA fragments comprising the promoter (amplified with primers GCn_002 and R812) and the SH4_sgRNA scaffold (amplified with primers F812 and GCn_003) using Phusion® High-Fidelity DNA (New England Biolabs). H1.51 or H1.52 was synthesised and inserted into pSH4_NGHyg plasmid by Twist Biosciences, yielding pSH4_NGHyg_04168 and pSH4_NGHyg_02924. The repair template, which contained H1.51 or H1.52 harboured with homology to up- and down-stream of Safe haven 4 (SH4) was amplified with primers SH4_RT_Fw and SH4_RT_Rv from pSH4_NGHyg_04168 or pSH4_NGHyg_02924 using ExTaq polymerase (Takara Bio). All PCR products were purified by QIAquick PCR purification kit (QIAGEN) in accordance with the manufacturer’s protocol.

The CAS9 cassette, the SH4_sgRNA and the repair template were co-transformed into the H1.51 or H1.52 deletion strains by electroporation method adapted from Fan et al (2020). Briefly, *C. neoformans* strains were grown overnight in 3 ml YPD at 30°C with shaking at 200 rpm. The overnight cultures were inoculated in 100 ml YPD to OD_600_ = 0.2 and incubated at 30°C with shaking until the cultures reached a final OD_600_ of 0.6 -1.0. Cells were collected by centrifugation and washed twice with ice-cold water. The pellet was resuspended in 10 ml ice-cold electroporation buffer (10 mM Tris-HCl (pH 7.5), 1 mM MgCl_2_, and 270 mM sucrose) supplemented with 1 mM DTT. After 1 h of incubation on ice, cells were harvested by centrifugation and were resuspended in 250 µl electroporation buffer. 45 µl of cell suspension was mixed with 700 ng *CAS9*, 700 ng SH4_sgRNA and 2 µg of repair template DNA. The cell-DNA mix was transferred to a 2 mm electroporation cuvette (Fisherbrand, FB102). Cells were electroporated using five positive polarity 450 V/1.5mSec poring pulses at 50 mSec intervals, followed by 5 50 V/50mSec polarity-exchanged transfer pulses at 50 mSec intervals on a Nepa Gene ELEPO21 electroporator. The electroporated cells were resuspended in 1 ml YPD and cultured at 30 °C for 2 h, plated on YPD agar plates supplemented with 200 µg/ml hygromycin (Gibco, 10687010), and incubated at 30 °C for 2-3 days. Transformants were verified by PCR to confirm the reintegration of the H1.51 or H1.52 to the desired locus. The primers used for the construction and verification of the reintegrated strain were listed in (Supplementary Data 4**)**.

### Cell harvesting

For each culture, 40 mL was harvested for RNA/DNA extraction by centrifugation (3 min, 4000 rpm, 4 °C), washed twice with ice-cold PBS, and stored at -80 °C. The remaining 115 mL was cross-linked with 0.67% formaldehyde for 12 min at room temperature, quenched with 0.5 M glycine for 7 min, harvested by centrifugation, washed twice with ice-cold PBS, and freeze-dried overnight.

### Chromatin Immunoprecipitation

Freeze-dried cell pellets were broken into fine powder with 0.5 mm zirconia/silica beads in a FastPrep 24 homogeniser (MP Biomedicals) without buffer for three cycles (50 s at 8 m/s followed by 90 s chilling on ice) and then with 250 μl of ChIP lysis buffer (50 mM HEPES pH 7.5, 140 mM NaCl, 1 mM EDTA, 1% Triton X-100, 0.1% Deoxycholate, 1 mM PMSF, 3x Roche complete protease inhibitor cocktail) for another four cycles. The lysate was recovered by burning a hole in the top and bottom of the screw-cap tubes using a hot needle, placing them on top of a pipette tip in a 15 ml conical tube on ice and centrifugation at 1200 rpm for 2 min at 4 °C. Lysate was then diluted to 1 ml with ChIP lysis buffer without detergents (50 mM HEPES pH 7.5, 140 mM NaCl, 1 mM EDTA, 1 mM PMSF, 3x Roche complete protease inhibitor cocktail) and transferred to a milliTUBE 1 ml with AFA Fibre (Covaris #520135).

Samples were then sheared with the Covaris E220 Focused ultrasonicator for 13 minutes at 5 °C (settings: 150-W peak power / 30% duty cycle /200 cycles per burst; samples sheared in 30 s cycles). The sheared chromatin was transferred to a 1.5 ml DNA LoBind tube (Eppendorf #0030108051, used for all subsequent steps) and centrifuged at 21,000 g for 15 min at 4 °C to remove insoluble cellular debris. Twenty microliters of sheared chromatin suspension were saved as Input DNA control and stored at −20 °C.

The detergent concentration of the chromatin suspensions was adjusted to 1% Triton X-100 and 0.1% Deoxycholate to perform immunoprecipitation in ChIP lysis buffer. Immunoprecipitation was carried out with protein A/G agarose beads (Santa Cruz Biotechnology, sc-2003) washed twice in ChIP lysis buffer without protease inhibitors. The sheared chromatin was subjected to pre-clearing to reduce proteins that non-specifically bind beads by incubation with 30 μl of washed agarose beads for 1 hour at 4°C on a rotator. After centrifugation at 5000 rpm for 1 min at 4 °C to pellet the beads, the supernatant was transferred to a new tube and incubated overnight with 20 μl of washed beads and either 3 μl of H3K4me2 antibody (Active Motif, 39141) or 2 μl of H3K9me2 antibody (Abcam, ab1220) at 4 °C on a rotator. The next day, beads were washed twice in 1 ml ChIP lysis buffer without protease inhibitors, once in 1 ml ChIP lysis buffer containing 0.5 M NaCl, once in 1 ml LiCl wash buffer (10 mM Tris-HCl pH 8.0, 0.25 M LiCl, 0.5% NP-40, 0.5% deoxycholate, 1 mM EDTA), and twice in 1 ml TE buffer (10 mM Tris–HCl pH 8, 1 mM EDTA), all at 4°C. The bound chromatin was eluted in 62.5 μl of ChIP TES buffer (50 mM Tris- HCl, pH 8.0; 10 mM EDTA; 1% SDS) at 65˚C for 10 minutes. After centrifugation, supernatant was transferred to a new tube. A second elution with 62.5 μl of ChIP TES buffer was performed so that the final elution volume was 125 μl. 105 μl ChIP TES buffer was added to each Input sample. All Input and IP samples were incubated for 16 hs at 65˚ C for reverse crosslinking. After reverse crosslinking, samples were treated with 1,25 μl of 20 mg/mL RNase A for 2 h at 37 ˚C, followed by treatment with 6.25 μl of 20 mg/mL Proteinase K for 2 hours at 42 ˚C. DNA was purified using a Qiagen PCR purification kit (Qiagen #28106).

### DNA and RNA Extraction

Genomic DNA was extracted from pooled cells (three biological replicates per strain) using the DNeasy Plant Pro Kit (Qiagen #69204). Total RNA was extracted from three individual biological replicates per strain using the Monarch Total RNA Miniprep Kit (NEB T2010). All extractions followed manufacturer’s protocols. DNA and RNA samples were quantified using a Qubit 4.0 fluorometer and quality assessed using RNA integrity numbers (RIN) and DNA integrity numbers (DIN) from an Agilent TapeStation 4200 **(**Supplementary Data 5**)**.

### Sequencing

Library preparation and sequencing were performed by the Exeter Sequencing Service. ChIP and genomic DNA libraries were prepared using the QIAseq Ultralow Input Library Kit (Qiagen #180497), while RNA libraries were prepared using the TruSeq Stranded mRNA Library Prep Kit (Illumina #20020594). All libraries were sequenced on a NovaSeq 6000 platform generating 150 bp paired end reads. Raw sequences were quality-trimmed using fastp v0.23.1, removing bases below Q22 from 3’ ends and discarding reads shorter than 75 bp.

### Evolutionary analysis

Reference genome assemblies, predicted peptide sequences, coding sequences (CDS), and GFF3 annotation files were downloaded from NCBI for 50 basidiomycete species. The Synima pipeline was used to identify orthologous gene relationships across all species using BLAST-based reciprocal best hits followed by OrthoFinder clustering. H1 linker histone orthologs were extracted and their corresponding peptide and nucleotide sequences retrieved for downstream analysis. Proteins were aligned with MAFFT (v7.149b)^75^. Codon alignments were generated with PAL2NAL and exported in PAML format^57^. Alignments were trimmed with TrimAl to remove columns with >50% gaps and <90% conservation^76,77^.

A maximum-likelihood gene tree for H1 linker histones was inferred using IQ-TREE 2 (v2.3.6) under the codon model with 1000 bootstrap replicates^78^. The best-fitting substitution model was GTR + F3×4 + G4. Branch models in CODEML (PAML v4.10) were used to test for rate differences between paralogs^79^. We compared a null model (model = 0, NSsites = 0) estimating a single ω for all branches against alternative models (model = 2, NSsites = 0) allowing different ω values for foreground (labelled) vs. background branches. Statistical significance was assessed using likelihood ratio tests with a χ² distribution (df = 1, α = 0.05).

Complementary site-specific and branch-site selection analyses were performed using HyPhy^80^. RELAX tested for relaxed or intensified selection on foreground branches, aBSREL identified episodic diversifying selection on individual branches, MEME detected episodic diversifying selection at individual codon sites, FEL identified sites under pervasive purifying or diversifying selection and BUSTED tested for gene-wide evidence of positive selection on foreground branches. Pairwise sequence conservation matrices were generated using Geneious Prime (v2025.2) from the MAFFT protein alignment, calculating percent identity for all H1 paralog pairs. Sequences were classified into H1.51 and H1.52 paralog clades based on phylogenetic relationships. Mean conservation was calculated for intra-clade comparisons (within H1.51, within H1.52) and inter-clade comparisons (H1.51 vs H1.52) to quantify paralog divergence. Conservation matrices were visualised as annotated heatmaps using pheatmap in R with paralog clades displayed as row and column annotations.

### C-terminal domain identification and Linker histone tail property analysis

Full-length H1 paralog sequences (*n* = 34: Paralog 1, *n* = 17; Paralog 2, *n* = 17) from *Cryptococcus* species were analysed using InterProScan in Geneious Prime to predict functional domains and identify the boundary between the globular domain and C-terminal intrinsically disordered region. C-terminal sequences were extracted from residue 100 to terminus, yielding tails of 35-95 amino acids (Paralog 1, 80 ± 22 residues; Paralog 2, 94 ± 1 residues). Extracted sequences were analysed using custom Python scripts to calculate arginine:lysine ratio as R/(R + K), where R and K represent arginine and lysine counts, net charge per residue (NCPR) as (R + K + H-D-E)/length, where R, K, H, D, E represent counts of arginine, lysine, histidine, aspartate, and glutamate. Additionally, the longest proline-free region was determined from the maximum contiguous stretch lacking proline, while hydrophobicity values were extracted for each amino acid, using the Kyte-Doolittle scale on Geneious Prime and then averaged across C-terminal positions. Data normality was assessed using Shapiro-Wilk tests. Group comparisons used two-tailed Welch’s t-tests (unequal variances) for normally distributed data or two-tailed Mann-Whitney U tests (non-parametric) for non-normally distributed data. Effect sizes calculated as Cohen’s d.

### rDNA copy number estimation

To assess rDNA array stability, per-base read depth across the 25S, 5.8S, 18S, and 5S rRNA genes was calculated using SAMtools mpileup (v1.15.1). For Illumina short-read data, paired-end reads were aligned to the individual rDNA gene sequences using BWA-MEM (v0.7.17) and per-base coverage was normalised to the genome-wide median depth for each strain. For ONT long-read data, reads were aligned to the full *C. neoformans* H99 CNA2 reference genome using minimap2, and rDNA coverage was similarly normalised to genome-wide median depth. rDNA retention under host-mimicking conditions was quantified as the ratio of mean normalised rDNA coverage under stress (72 h, 37°C, 5% CO₂, PBS with 10% heat-inactivated FBS) relative to standard YPD culture (30°C).

### RNA-seq analysis

The *C. neoformans* H99 reference genome (CNA2; GCA_000149245.3) was indexed using STAR v2.7.5 with splice junction overhang set to 100 bp and genomeSAindexNbases to 11. RSEM v1.3.3 was used to prepare the reference transcriptome with STAR as the alignment engine^81,82^. Read quality was assessed using FastQC and trimmed using fastp v0.23.1. Paired-end RNA-seq reads were aligned and quantified using RSEM with the STAR aligner in strand-specific mode (forward-prob 0.0). RNA sequencing of *C. neoformans* isolates generated 5.7 to 19.5 million paired end reads per library, 85–96% of which aligned concordantly to the reference genome (Supplementary Data 6). Differential expression analysis was performed using the Trinity RNA-seq pipeline (v2.15.2) with DESeq2, using the two-sided Wald test to assess gene-level log₂ fold changes. Differentially expressed genes were defined by FDR ≤ 0.05 and log₂FC ≥ 1.0^83,84^.

GO term and KEGG pathway annotations for the *C. neoformans* H99 (CNA2) reference genome were obtained using InterProScan, EggNOG-mapper, and Pfam^85–87^. GO enrichment analysis was performed using Fisher’s exact test, with Biological Process, Molecular Function, and Cellular Component ontologies analysed independently to reduce multiple testing burden. Gene sets of 5-500 members were tested, semantic redundancy reduced using rrvgo (similarity threshold 0.7), and significance defined as FDR < 0.05 after Benjamini-Hochberg correction. KEGG pathway enrichment was computed using Fast Gene Set Enrichment Analysis (FGSEA) with 10,000 permutations and FDR ≤ 0.05^88^. Differentially expressed genes were defined by FDR ≤ 0.05 and log₂FC ≥ 1.0.

### ChIP-seq analysis

Paired-end FASTQ files were aligned to the *C. neoformans* H99 CNA2 genome using BWA-MEM v0.7.17^89^. Alignments were processed with Samtools v1.15.1 to generate coordinate-sorted BAM files^85^. Duplicates were marked with Picard MarkDuplicates and subsequently filtered using samtools view with flags -f 2 (proper pairs), -F 1804 (excluding unmapped reads, secondary alignments, QC failures, and duplicates), and -q 30 (minimum mapping quality). Library complexity was assessed using NRF (non-redundant fraction), PBC1, and PBC2 metrics. Fragment size distributions were validated using Picard CollectInsertSizeMetrics and Fraction of Reads in Peak (FRIP) scores were calculated using custom scripts in our github repository **(**Supplementary Data 7**)**.

Peaks were called using MACS3 with mark-specific parameters. H3K4me2 used narrow peak mode (-q 0.05, --nomodel, sample-specific extension sizes 121-217 bp, --keep-dup all), while H3K9me2 used broad peak mode (-q 0.05, --broad-cutoff 0.1, --max-gap 1000, --min-length 500, --nomodel --extsize 217, --keep-dup 2). Both analyses used -f BAMPE, effective genome size 18.6 Mb, and --SPMR for signal track generation^90,91^.

Biological replicates were merged using a union approach. Overlapping peaks were collapsed and the peak with the strongest q-value retained using custom R scripts to generate a non-redundant consensus peak set. Peak annotation was performed on consensus peaks using ChIPseeker (v1.42.1) with TSS regions defined as ±1.5 kb^92^. Multi-gene annotation identified all genes whose promoters overlapped each peak, capturing comprehensive gene-peak associations particularly important for broad domains. Genomic coverage was calculated by computing total coordinates covered by peaks (bp) divided by total genome size. FE-Normalised BigWig tracks were generated using deepTools bamCoverage (10 bp bins). Signal distributions at TSS ( ± 1.5 kb) were quantified using computeMatrix in reference-point mode and visualised with plotHeatmap and plotProfile to genetae metagene plots^93^.

### H3K4me2 domain boundary analysis

To determine whether H1.52 constrains H3K4me2 domain architecture, inter-peak gaps ( < 500 bp) between adjacent KN99α H3K4me2 consensus peaks were identified. H3K4me2 signal within these intervals was quantified from FE-normalised bigWig tracks for each strain (n = 1000 randomly sampled gaps). Signal differences between strains were assessed using two-sided Wilcoxon rank-sum tests. Gap filling was defined as intervals where ≥50% of the gap was covered by H1.52Δ H3K4me2 peaks, stratified by gap distance. Meta-profiles of H3K4me2 signal centred on KN99α peak midpoints ( ± 2 kb) were generated using deepTools computeMatrix in reference-point mode across 2000 randomly sampled peaks. H1.52Δ peak dynamics were classified by BEDTools intersect overlap with KN99α peaks: retained (overlaps one KN99α peak, width ratio ≤1.5×), broadened (overlaps one KN99α peak, width ratio >1.5×), merged (overlaps ≥2 KN99α peaks), or de novo (no KN99α overlap). Peak width distributions across classes were compared using two-sided Wilcoxon rank-sum tests.

### Chromatin state classification and co-marked gene analysis

Genes were classified by promoter chromatin state using ChIPseeker annotations ( ± 1.5 kb from TSS). H3K4me2-only, H3K9me2-only, co-marked (both marks), or unmarked. Expression changes for each chromatin class in H1.52Δ relative to KN99α were assessed using two-sided one-sample Wilcoxon signed-rank tests against a median of zero. Enrichment of significantly upregulated genes (FDR < 0.05, log₂FC > 0) within each class was tested using two-sided Fisher’s exact tests, with odds ratios and 95% confidence intervals reported. Gene Ontology enrichment of upregulated co-marked genes was performed using clusterProfiler with Benjamini–Hochberg correction (FDR < 0.05).

### XTT assay

For metabolic activity assessment cells were seeded at an OD₆₀₀ of 0.001 in PBS containing 10% heat-inactivated FBS into 96-well plates. Cells were incubated for 72 h at 37 °C in 5% CO₂. Subsequently, 50 µl of XTT labelling mixture (Cell Proliferation Kit II, Roche, #11465015001) was added to each well according to the manufacturer’s protocol and incubated for 4 h at 37 °C in 5% CO₂. The reduction of XTT to formazan by metabolically active cells was measured by reading absorbance at 450 nm with a reference wavelength of 650 nm on a Tecan Spark plate reader. Three independent biological replicates were performed, each containing four technical replicates. Wells containing only media without cells were included as negative controls to account for background absorbance.

### *C. neoformans* growth curve

After extended culture in PBS containing 10% heat-inactivated FBS for 72, 120, and 168 hours (3, 5, and 7 days), cells were washed three times with PBS, reintroduced into YPD medium at a final OD_600_ of 0.01. Cultures were then transferred to a Tecan plate reader, where optical density at 600 nm (OD_600_) was measured every 30 minutes to monitor growth recovery kinetics. Growth parameters were calculated from averaged technical replicates: maximum OD_600_ was determined as the peak absorbance value, lag time was defined as the time required to reach OD_600_ 0.2, and doubling time was calculated from exponential phase growth (OD_600_ 0.2–0.5) using linear regression of log-transformed OD values. Data normality was assessed using Shapiro-Wilk tests and homogeneity of variance was evaluated using Levene’s test. Since data violated normality assumptions (*p* < 0.05), non-parametric Mann-Whitney U tests were used for pairwise comparisons between mutant strains and KN99α. Multiple testing correction was applied using both Bonferroni and Benjamini-Hochberg false discovery rate (FDR) methods to control for family-wise error rate across four strain comparisons per timepoint. Statistical significance was determined at Bonferroni-adjusted *p* < 0.0125 or FDR-adjusted *p* < 0.05.

### Galleria mellonella infection model

Strains were cultured overnight in YPD medium at 30 °C with shaking, washed, and resuspended in PBS prior to infection. *Galleria mellonella* larvae (UK waxworms, Sheffield) were injected with 1 ×10^4^ cells of *C. neoformans* strains KN99α, ∆H1.51, ∆H1.52, ∆H1.51:H1.51 and ∆H1.52:H1.52 into the left rear proleg using a Hamilton 750 syringe (Hamilton Company, UK). The inoculum was supplemented with ampicillin (10 µg per larva) and kanamycin (3 µg per larva) to prevent bacterial contamination. The average volume delivered to each larva was 10 µl. For each condition, 20 larvae were injected per replicate, with a total of two replicates performed. Larvae were incubated at 37 °C for 10 days, with survival recorded daily. A log-rank (Mantel-Cox) test was performed to assess if there was a significant difference in the survival of larvae, where *P* < 0.05. Analysis and visualisation of Kaplan-Meier survival curves was performed using Graphpad Prism (v10.4.0). Colony-forming units (CFUs) were enumerated from dead larvae to determine fungal burden at the time of death. Dead larvae were homogenised in 1 ml PBS by bead beating, serially diluted, and plated on YPD medium supplemented with 100U penicillin-streptomycin for CFU counts.

### Statistics and reproducibility

No statistical methods were used to predetermine sample sizes. Sample sizes followed established conventions for each experimental approach. No data were excluded from analyses. All replication attempts were successful. *Galleria mellonella* larvae were randomly assigned to treatment groups. Randomisation was not applicable to other experiments as all strains were processed under identical conditions. Blinding was not performed as all readouts are objective quantitative measurements analysed by standardised computational pipelines. Differential gene expression was determined using limma-voom via Trinity v2.15.1 (FDR < 0.05, log₂FC > 1). GO enrichment used Fisher’s exact test with Benjamini-Hochberg correction. KEGG pathway enrichment used GSEA (FDR < 0.05). Paralog biochemical properties were compared using two-sided tests with Cohen’s d effect sizes. Growth curve parameters were compared using two-sided Mann–Whitney U tests with Bonferroni correction. Galleria survival was analysed by Kaplan–Meier estimation with log-rank test. CFU data were log₁₀-transformed and analysed by one-way ANOVA with Tukey’s HSD post-hoc test. Molecular evolution analyses used PAML CodeML branch models and HyPhy (RELAX, aBSREL, BUSTED, MEME). Sample sizes, statistical tests, exact *p*-values, and effect sizes for each experiment are reported in the corresponding figure legends.

### Reporting summary

Further information on research design is available in the Nature Portfolio Reporting Summary linked to this article.

## Supplementary information

Transparent Peer Review file

Supplementary Information

Description of Additional Supplementary Files

Supplementary Data 1

Supplementary Data 2

Supplementary Data 3

Supplementary Data 4

Supplementary Data 5

Supplementary Data 6

Supplementary Data 7

Supplementary Data 8

Reporting Summary

## Data Availability

All data supporting the findings of this study are available within the article and its Supplementary Information files. The multi-omics datasets generated in this study have been deposited in the NCBI Sequence Read Archive (SRA) under BioProject accession P, comprising transcriptomics (RNA-seq), chromatin immunoprecipitation sequencing (ChIP-seq; H3K4me2 and H3K9me2), and whole-genome DNA-seq raw sequencing data (FASTQ files). Processed RNA-seq gene expression matrices and ChIP-seq bigWig files are available at https://github.com/gp517/h1-chromatin-remodeling-cryptococcus. The *Cryptococcus neoformans* var. *grubii* H99 reference genome used for read alignment is available from NCBI under assembly accession G. Numerical source data underlying all graphs and charts in the main and Supplementary Figs. are provided in Supplementary Data 8. All other data supporting the findings of this study are available from the corresponding author upon reasonable request.

## Code availability

All ChIPseq pipelines and source code used in this study have been included in Chipseq Analysis Tools (CAT) available at https://github.com/rhysf/Chipseq_analysis_tools and https://github.com/gp517/h1-chromatin-remodeling-cryptococcus, and archived at Zenodo (https://doi.org/10.5281/zenodo.20315263)^94^.
