# Supplementary material for: Neofunctionalization of H1 linker histones drives divergent gene expression and histone methylation in Cryptococcus neoformans
Source: Commun Biol. 2026 Jun 10;9:1232. doi: 10.1038/s42003-026-10432-4 (PMC13594160; doi:10.1038/s42003-026-10432-4)
Supplement: Supplementary file 2 [file 42003_2026_10432_MOESM1_ESM.pdf]

nature portfolio

Peer Review File

Neofunctionalization of H1 linker histones drives divergent gene expression and histone methylation in Cryptococcus neoformans

Corresponding Author: Dr Rhys Farrer

This file contains all reviewer reports in order by version, followed by all author rebuttals in order by version.

Version 0:

Reviewer comments:

Reviewer #1

(Remarks to the Author)
Cryptococcus species cause deadly infections that are often overlooked and certainly understudied. In this manuscript, the authors aim to improve our understanding of one aspect of Cryptococcus biology, by providing insight in the epigenome of C. neoformans, a particularly dangerous human fungal pathogen. The authors perform important benchmarking work to establish an appropriate workflow, and then use their optimized approach to uncover new information about the organization of the C. neoformans epigenome. They find evidence supporting an important role for the linker histone H1 in control of H3K4me2 and H3K9me2, primarily in sub telomeric regions, and hypothesize from their data that this organization may be important for regulation of fungal genes involved in virulence and drug resistance. The manuscript is clearly written overall, but the results section can be somewhat dense at times and sometimes the thread is lost. A few suggested improvements are listed in the specific comments below.

Major Comments
1. Line 109-116, the authors introduce multiple strains/lineages without much background. A few more words about their importance and selection could set the stage for the experiments performed. At a minimum, the H99O lineage should be briefly defined.
2. The paper focuses primarily on the comparison of KN99_lab with the H1 knockout strain. As such, would it be worth including both of these strains in all the comparisons with KN99 in Figure 2 or alternatively moving the KN99 comparisons to supplemental?
3. Line 178-180, the authors state that the upregulation of three genes in the H1 deletion strain is explained by their location in a deleted region in the KN99_lab strain. I believe the assumption is that deletion is a way of repressing these three genes in the absence of the normal H1 regulation. The line could use a few more words to clearly state the hypothesis or better would be to push this all to the discussion, where the authors already more fully describe their theory. They also correctly point out in the discussion that it remains a hypothesis.
4. It may be helpful to add zoomed-in subsets for Figure 4. Currently it is small, and difficult to discern much detail.
5. Figure S4 is too small, even on a large monitor. Please increase the size, possibly by moving panels one on top of the other.
6. Lines 299-325 are dense with information. A table could be a more useful way to compare some of these values and help the reader more easily digest this information.
7. Line 355-361, should this section refer the reader to a Figure or Table?
8. Figure S7, the rightmost peaks called significant (blue boxes) for H3K9 seem a bit oddly placed. Is there something relevant worth discussing here?
9. The authors describe the substantial genetic diversity of the strains and then draw conclusions about the function of H1 using a knockout. The arguments would be significantly benefited by some form of complementation of H1. Is such a strain available that might be used to test one or two phenotypes that were pulled out of the ChIP-seq data?

Minor Comments
1. At times the bioinformatic tools are introduced too briefly, for example, RAxML should be defined (with a couple words) in the Figure 1 legend.
2. Line 342-343, consider adding abbreviations for (CT) and (AG), which are used multiple times later in the paragraph.

3. Line 669, “FungalDb release 58” should be “FungiDB release 58”
4. Does the result of the GO analysis change significantly if you provide a background gene list based on expression data rather than using the whole genome annotation as a reference?

Reviewer #2

(Remarks to the Author)
The manuscript entitled “Genomic and epigenomic variation in pathogenic Cryptococcus species“ by Goodisman et al. describes comparative genomics of C. neoformans isolates, and additionally, epigenetic analysis of two C. neoformans strains (own dataset) vs. C. gattii (public dataset). The authors analyze a deletion mutant of the linker histone gene H1.5 (one of two copies), as well as quantify peaks for the formally euchromatic mark H3K4me2 and the heterochromatic mark H3K9me3. They find co-localization of these two marks in C. neoformans at subtelomeres and centromeres, which stands in contrast to the C. gattii dataset and also datasets published for other fungi. Furthermore, they report discrepancies in called peaks between different methods, which is an interesting result.

In principle, the study is timely and very interesting to a broad audience. However, in my opinion, some controls are missing to backup the shown data (as detailed below). And it ultimately remains descriptive, without conclusion on how this study is deepening our understanding of Cryptococcus epigenetics. I suggest to dive deeper into understanding the function of histone H1.5. This could be in the form of generating a double deletion mutant as I am suggesting below, or by ChIPseq experiments with a tagged version of their deleted H1.5 gene (as suggested in lines 499-502), in order to understand the localization of the encoded protein and thereby, make a connection to the detected epigenetic profiles and differentially expressed genes (DEGs).

Detailed comments:
1. I couldn’t find any description on how the deletion mutant of H1.5 was generated. If it originates from a previous study, please include the reference in the methods, otherwise, include a description of how the strain was generated. There is a second paralogous gene of H1.5 in the genome (line 175). Please reason why the second copy was not deleted individually, and why no double deletion was attempted, in order to analyze the full loss-of-function phenotype? In N. crassa, the single H1 is not an essential gene (Seymour et al., 2016). Do the authors expect differential targeting of the two paralogs to chromatin, and no functional redundancy? I would suggest to perform a double deletion to be able to evaluate the full effect of H1.5 gene loss. A plate assay should be included to be able to evaluate the impact on growth.
2. The authors describe how their different ChIPseq peak calling methods resulted in differences between their own C. neoformans dataset and the public C. gattii dataset (line 241). And that there was not one method applicable for both strains. If I am not mistaken, there is no figure which just shows the ChIPseq reads along the genome (e.g., simply visualized with IGV). This would help to evaluate the quality of the performed experiments, i.e., show whether there is enrichment in specific areas over others. Additionally, the authors should comment on differences in cultivation conditions used for the ChIPseq experiments of C. neoformas vs. C. gattii, which could result in different epigenetic profiles.
3. Quantification of the C. neoformans called peaks revealed 5x more H3K4me2 peaks and 5x less H3K9me3 peaks in the H1.5 mutant (line 292). Here, I suggest a simple Western blot to visualize this, and help verify the peak calling method. Additionally, this result does not seem to match the gene expression data of WT vs. mutant, where there were only very few DEGs?
4. Lines 299 and 355: It is quite surprising that H3K4me2 is enriched at subtelomeres and centromeres in C. neoformans. Does this in any way match the gene expression data, i.e., are these regions expressed under the cultivation conditions chosen?

Version 1:

Reviewer comments:

Reviewer #1

(Remarks to the Author)
The revised manuscript is greatly improved. It describes the adaptive neofunctionalization of linker histone H1 in the dangerous human pathogen Cryptococcus neoformans. The authors reveal divergent roles for H1.51 and H1.52 in the yeast, which contribute to chromatin compaction and regulation of metabolism. Where deletion of H1.51 plays only a minimal role on the transcriptome in vitro, deletion of H1.52 was shown to drastically alter the transcriptome and chromatin landscape. The manuscript is technically detailed, but at times lacks information in the results relevant to understanding the biology of the findings. More context for the results might help a broad readership grasp the relevance of the results. Below are a few suggestions that might help improve the impact:

Major Comments
1. Line 130-146, some information about the tools should be added for a more general audience. Alternatively, could this be simplified somehow (e.g., some parameters moved to the methods?). Similarly, Line 148-152 should be reworded. This is a key conclusion, but is currently difficult to understand. In general, the manuscript would benefit from clearer summary sentences at the end of the results subsections.
2. Line 179-180, please define host-mimicking stress here. It would also be valuable to state the source of knockouts here (e.g., FGSC). In general, (very) brief descriptions of the method used to construct knockouts and produce complementation strains would also be helpful to readers outside the immediate field in assessing the experiments.

3. Line 214, this header is not supported by the data, as no assessment of ribosome biogenesis itself or translational output was performed. It should be reworded to more accurately reflect the data presented.
4. Line 243-252, I believe the authors are suggesting that deletion of H1.52 results in halving of the array and complementation then causes it to expand back to K99 levels? If so, this is not totally clear from the text yet (but is clearer in the discussion). Just a curiosity, how long does this process take (in other studies), and does that timeline fit your experimental situation? Related, there is no figure legend or description of the colored lines for Figure 4d.

Minor Comments
5. Line 29, “host-relevant conditions” could be a bit more specific. Later (Line 91), host-mimicking is used. Line 28-31, recovery to what?
6. The manuscript has a number of minor typos, e.g., Line 48 should be encompass; Line 83, adjust “including including”; Line 206, “were”; Line 280, “heterochromatic”; Line 327, “where siginifigantly” should be “were significantly”; Line 528 and 537, “Safe Heaven 4” should be “haven”. Some more proofreading throughout could be beneficial.
7. The concluding paragraph of the intro on Lines 86-100 could be more impactful. The study is interesting, but this paragraph does not really link the findings to a broader context.
8. Line 109-112, please provide some information on the qualifications necessary to be considered a paralog.
9. Figure 3B-E, the names of genes are very hard to read. Could they be made bigger or changed to gray/black (or removed if unnecessary)?
10. Line 616-621, the authors state that nucleic acid quality was assessed, but no information is provided on the result of this assessment. Is the quality comparable between samples?
11. The complemented strain is denoted a few different ways in the text. It would be easier for the reader if this was standardized. Similar, on Line 344, are the authors referring to a complemented strain with KN99α:H1.52? This notation seems to be different from previous parts of the text.
12. Line 336, “181.4% cell viability”, I understand the graph, but it seems odd that cells can be more than 100% viable. Could there be a different way to phrase this (e.g., relative metabolism)?
13. Line 304, is “metabolic shutdown” the correct term here? I am again not sure the data supports such a strong conclusion.
14. Line 468-470, is a reference missing?
15. Lines 466-491, the authors hint at changes in gene expression of relevant modulators resulting from the deletion of H1.52. Might some of the phenotypes attributed to H1.52 then be indirect via changes to proteins like Clr4 or the RNAi components? This could be mentioned as a potential confounding factor of the interpretation. The authors could also consider to soften “directly govern” on Lines 501-506.

Reviewer #2

(Remarks to the Author)
I thank Paul and colleagues for providing this much improved manuscript which addressed most of my concerns. The authors characterize two paralogs of the linker histone H1 in the important opportunistic human pathogen Cryptococcus neoformans. Even though the single deletion mutants didn’t exhibit a pathogenicity defect (in the performed infection model), the authors describe interesting divergent evolution of the two paralogous genes, and additionally, find an impact on two histone marks and (subsequently) gene expression for the H1.52 deletion mutant. In the improved version, I’d like to kindly ask the authors to comment on the following:

- Line 28: In the fungal field, facultative heterochromatin specifically refers to H3K27-marked heterochromatin, so I’d recommend using another term, such as H3K9-marked heterochromatin islands (see for example doi.org/10.1038/s41586-020-2706-x).
- Line 249: Was the complementation construct used to transform the deletion mutant of H1.52? Are the authors suggesting that rDNA loss occurred in a progressive manner through cultivation of the deletion mutant? This means that part of the phenotype of H1.52 deletion is through modification of another genomic location (rDNA), and additionally it means that wild type, deletion and complementation mutants vary in more than one locus. The description of the deletion phenotype should mention this issue, and the title of this section should be changed to reflect this: “H1.52 Deletion Impairs Ribosome Biogenesis and Translation in C. neoformans”.
- Line 286-287: “H3K4me2 expansion” implicates spreading which is misleading. One can assume that derepression of gene expression goes in line with increased H3K4 methylation. Could the authors correlate their RNA-seq data to the ChIP-seq data, and determine how many genes are upregulated in the H1.52 deletion mutant and at the same time gain H3K4 methylation?
- Line 292: Thank you to responding to my previous comment on subtelomeric H3K4 methylation, however, I still think this is not fully resolved although the comparison to C. gatii was removed. The chromosome-wide view of the H3K4me2 mark on chromosome 2 reveals that substantial H3K4 methylation is present at the subtelomeres in the wild type. Could the authors show the other chromosomes in the supplementary to see if this is the case consistently? Looking at chromosome-wide H3K4me2 in C. deuterogatii (doi.org/10.1371/journal.pgen.1009743), this is not really the case. Could the authors add a track which visualizes gene expression under these conditions, to see if this goes in line with gene activation at subtelomeres?
- Line 489: My apologies, I don’t follow this conclusion, since I don’t see where it was demonstrated that H3K4 methylation was acquired at downregulated genes in the deletion mutant (see my questions above).

Open Access This Peer Review File is licensed under a Creative Commons Attribution 4.0 International License, which permits use, sharing, adaptation, distribution and reproduction in any medium or format, as long as you give appropriate credit to the original author(s) and the source, provide a link to the Creative Commons license, and indicate if changes were made.
In cases where reviewers are anonymous, credit should be given to 'Anonymous Referee' and the source.
The images or other third party material in this Peer Review File are included in the article's Creative Commons license, unless indicated otherwise in a credit line to the material. If material is not included in the article's Creative Commons license and your intended use is not permitted by statutory regulation or exceeds the permitted use, you will need to obtain permission directly from the copyright holder.
To view a copy of this license, visit https://creativecommons.org/licenses/by/4.0/

Manuscript: “Neofunctionalization of H1 linker histones drives divergent gene expression and histone methylation in Cryptococcus neoformans”

Response to Reviewers - Communications Biology

We thank the reviewers for their thoughtful and constructive feedback on our original manuscript. The manuscript has undergone substantial revision since the initial submission, fundamentally refocusing the scope and expanding the experimental findings. Below we address each reviewer comment.

Major Comments

1. Line 109-116, the authors introduce multiple strains/lineages without much background. A few more words about their importance and selection could set the stage for the experiments performed. At a minimum, the H99O lineage should be briefly defined.

The revised manuscript has been substantially reorganized, removing the extended H99 lineage comparison that was a focus of the original submission. In the original manuscript, the H1 deletion strains (which were constructed in the KN99α parental background) were compared to KN99_lab as the experimental control for differential gene expression and ChIP-seq peak calling analyses. However, we found that the sequenced KN99_lab strain was not the actual parental strain from which the deletions were derived. This introduced confounding genetic variation between the control and experimental strains, making it impossible to determine whether observed differences resulted from H1 deletion or from pre-existing genomic differences between KN99_lab and the true parental strain KN99α. The revised manuscript eliminates this issue by resequencing the correct parental isogenic control strain KN99α, which is now used for differential expression and peak calling comparisons (DNA, RNA and CHIP-seq).

KN99α is the actual isogenic parental strain from which the H1.51Δ and H1.52Δ deletions were constructed, ensuring that control and experimental strains are genetically identical except for the H1 deletion. This means all observed phenotypes result specifically from H1 paralog loss rather than genetic background differences. Importantly, both the original and revised manuscripts align RNA-seq and ChIP-seq reads to the publicly available C. neoformans H99 reference genome for consistency within the field. The experimental design now centres on a simplified, isogenic strain panel outlined in the methods section (lines 512 - 514). Strains include KN99α wildtype, H1.51Δ and H1.52Δ deletion strains constructed in

this background, and genetic complementation strains (Δ H1.52:H1.52 and ΔH1.51:H1.51) to validate observed phenotypic and molecular findings. All strains share an isogenic background, eliminating the confounding genetic variation present in the original study design where the control strain did not match the parental strain.

The manuscript focus has shifted from H99 strain lineage comparisons to functional H1 paralog biology. The introduction now establishes biological context for *Cryptococcus* phenotypic plasticity and the potential role of chromatin regulation (lines 43-124), while we have added extensive comparative genomic analysis across 50 basidiomycete species revealing that H1 duplication is unique to the *Cryptococcus* genus (lines 130-149).

2. The paper focuses primarily on the comparison of KN99_lab with the H1 knockout strain. As such, would it be worth including both strains in all the comparisons with KN99 in Figure 2 or alternatively moving the KN99 comparisons to supplemental?

This concern has been addressed through reorganization of the experimental design. Figure 2 from the original manuscript showed genomic deletions across KN99_Lab which was the incorrect control strain. This figure and analysis have been removed entirely, and comparisons are no longer relevant because the control and knockout/complemented strains do not contain the absent or partially absent genes previously reported.

3. Line 178-180, the authors state that the upregulation of three genes in the H1 deletion strain is explained by their location in a deleted region in the KN99_lab strain. I believe the assumption is that deletion is a way of repressing these three genes in the absence of the normal H1 regulation. The line could use a few more words to clearly state the hypothesis or better would be to push this all to the discussion, where the authors already more fully describe their theory. They also correctly point out in the discussion that it remains a hypothesis.

This issue has been resolved through a fundamental change in experimental design. In the original manuscript, the strain used as the experimental control was labelled “KN99_Lab.” When this strain was sequenced, it was discovered to be a mislabelled H99 strain containing a >12 kb genomic deletion that encompassed three genes (CNAG_04096, CNAG_04097, and CNAG_04098). The H1.52 deletion strain appeared to show upregulation of these three genes relative to the “KN99_Lab” control. However, this apparent upregulation was an artifact, as the H1.52 deletion strain did not actually have this genomic deletion and therefore possessed and expressed these three genes, whereas the “KN99_Lab” control strain lacked

these genes entirely due to the deletion. This prevented clearly determining whether gene expression differences resulted from H1.52 loss or from the genomic content differences between the strains.

The revised manuscript eliminates this confounding factor by using the correct background strain, KN99α, as the experimental control strain. When we sequenced the actual parental strain (KN99α) used to construct the knockout and complemented strains, it was found not to contain the >12 kb deletion. Therefore, both the KN99α control strain and all derivative strains (H1.51Δ, H1.52Δ, and complemented strains) possess these three genes and can express them normally. The artefactual upregulation of CNAG_04096-04098 observed in the original manuscript no longer appears in the revised analysis, and this entire confounding issue has been removed from both the Results and Discussion sections.

4. It may be helpful to add zoomed-in subsets for Figure 4. Currently it is small, and difficult to discern much detail.

This figure has been removed from the manuscript due to substantial revision.

5. Figure S4 is too small, even on a large monitor. Please increase the size, possibly by moving panels one on top of the other.

This figure has been removed from the manuscript due to substantial revision.

6. Lines 299-325 are dense with information. A table could be a more useful way to compare some of these values and help the reader more easily digest this information.

The revision no longer includes section on H3K4/H3K9 enrichment over sub-telomeric regions. The revision focuses analysis to changes in peaks over protein coding genes.

7. Line 355-361, should this section refer the reader to a Figure or Table?

The revision no longer includes section on H3K4/H3K9 enrichment over centromeric regions. The revision focuses analysis on changes in peaks over genes.

8. Figure S7, the rightmost peaks called significant (blue boxes) for H3K9 seem a bit oddly placed. Is there something relevant worth discussing here?

This supplementary table has been removed from the revised manuscript.

9. The authors describe the substantial genetic diversity of the strains and then draw conclusions about the function of H1 using a knockout. The arguments would be significantly benefited by some form of complementation of H1. Is such a strain available that might be used to test one or two phenotypes that were pulled out of the ChIP-seq data?

This concern has been comprehensively addressed, as genetic complementation is now a central component of the revised manuscript. We constructed H1.52:H1.52Δ (H1.52 complementation strain) and KN99α:H1.51 (H1.51 complementation strain), with detailed methods for strain construction provided in lines 525-560. Complementation validates all major phenotypes reported in the manuscript. For transcriptional phenotypes, RNA-seq demonstrates that H1.52:H1.52Δ restores near-wildtype expression with only 1 DEG versus wildtype, compared to 1,561 DEGs in H1.52Δ (lines 191-193). Principal component analysis shows complement replicates cluster with KN99α wildtype (lines 196-197). For chromatin phenotypes, complementation demonstrates rescue of both H3K9me2 and H3K4me2 alterations. Complementation restores the dispersed facultative heterochromatin architecture that was lost in H1.52Δ (lines 274-276). H1.52:H1.52Δ shows intermediate H3K4me2 coverage of 21.4% between wildtype (15.0%) and knockout (27.7%), demonstrating partial rescue (line 289-292).

Detailed ChIP-seq analysis is presented in Figure 5 and Tables S2. Growth and metabolic phenotypes are also validated by complementation. Growth recovery assays show that H1.52:H1.52Δ exhibits delayed recovery compared to wildtype, which is opposite to the accelerated recovery of H1.52Δ. This suggests that H1.52 normally restrains metabolic activation (lines 407-410), validating that the enhanced metabolic activity in H1.52Δ is specifically due to loss of H1.52 function rather than genetic background effects.

Minor Comments

1. At times the bioinformatic tools are introduced too briefly, for example, RAxML should be defined (with a couple words) in the Figure 1 legend.

In the updated manuscript RAxML is no longer used. However, more detailed introductions to bioinformatic tools have been included in the revised methods section alongside references to papers describing the tool development.

2. Line 342-343, consider adding abbreviations for (CT) and (AG), which are used multiple times later in the paragraph.

In the revised manuscript we removed motif analysis, so cytosine and thymine vs adenosine and guanine peak enrichment analysis is no longer included in this version and therefore abbreviations no longer needed.

3. Line 669, “FungalDb release 58” should be “FungiDB release 58”

The revised manuscript no longer makes use of any FungalDb releases and concurrently this has been removed.

4. Does the result of the GO analysis change significantly if you provide a background gene list based on expression data rather than using the whole genome annotation as a reference?

We performed GO enrichment using an expression-defined background rather than the whole genome annotation. Specifically, the background (“gene universe”) was the set of genes retained after Trinity’s default low-expression filtering (--min_reps_min_cpm 2,1, i.e., genes with CPM > 1 in ≥2 replicates) and subsequently tested in the limma-voom differential expression model (n = 6801). Therefore, our reported GO enrichment results already reflect an expression-based background. We have revised the Methods to state this explicitly.
Detailed comments:

1. I couldn’t find any description on how the deletion mutant of H1.5 was generated. If it originates from a previous study, please include the reference in the methods, otherwise, include a description of how the strain was generated. There is a second paralogous gene of H1.5 in the genome (line 175). Please reason why the second copy was not deleted individually, and why no double deletion was attempted, to analyse the full loss-of-function phenotype? In N. crassa, the single H1 is not an essential gene (Seymour et al., 2016). Do the authors expect differential targeting of the two paralogs to chromatin, and no functional redundancy? I would suggest performing a double deletion to be able to evaluate the full effect of H1.5 gene loss. A plate assay should be included to be able to evaluate the impact on growth.

In the revised manuscript we detail that deletion mutants ΔH1.51 (CNAG_02924::NAT) and ΔH1.52 (CNAG_04168::NAT) were obtained from the Madhani laboratory collections via the

Fungal Genetics Stock Centre, with generation methodology detailed in the referenced publication (lines 515-517, 1116-1117). Complementation strains were constructed using biolistic transformation as described in Methods (lines 525-560).

The revised manuscript includes comprehensive analysis of both ∆H1.51 and ∆H1.52 using DNA sequencing, RNA-seq, and phenotypic assays. ChIP-seq was performed only on ∆H1.52 because ∆H1.51 showed no discernible transcriptional phenotype (2 DEGs vs. 1,561 DEGs in ∆H1.52), suggesting chromatin profiling of H3K4me2 and H3K9me2 in ∆H1.51 may be uninformative. The rationale for not pursuing a double deletion stem from our data demonstrating that H1.51Δ and H1.52Δ have dramatically different phenotypes, indicating they are not functionally redundant. The differential paralog functions revealed by our analysis justify the single deletion approach.

Molecular evolution analysis using PAML, RELAX, aBSREL, and MEME demonstrates that H1.51 and H1.52 evolved under different selective pressures (lines 131-132). H1.52 exhibits strong purifying selection ($\omega = 0.231$) consistent with conserved ancestral function, while H1.51 shows moderate purifying selection ($\omega = 0.499$) with episodic positive selection in clinical isolates. Biochemical analysis reveals different C-terminal tail properties of H1 paralogs which may affect chromatin binding (lines 157-175). These lines of evidence suggest neofunctionalization rather than redundancy, making individual deletion analysis more appropriate and informative for understanding H1 paralog-specific functions in Cryptococcus, rather than pursuing a double deletion that may confound interpretation.
In the revised manuscript microtiter Plate Optical Density (OD600) assays have been included to demonstrate growth recovery following extended culture under host-mimicking conditions at 3-, 5-, and 7-day timepoints in knockout and complemented strain relative to the wildtype (Figure 6) (lines 337 – 352).

2. The authors describe how their different ChIPseq peak calling methods resulted in differences between their own C. neoformans dataset and the public C. gattii dataset (line 241). And that there was not one method applicable for both strains. If I am not mistaken, there is no figure which just shows the ChIPseq reads along the genome (e.g., simply visualized with IGV). This would help to evaluate the quality of the performed experiments, i.e., show whether there is enrichment in specific areas over others. Additionally, the authors should comment on differences in cultivation conditions used for the ChIPseq experiments of C. neoformas vs. C. gattii, which could result in different epigenetic profiles.

Cultivation conditions varied substantially between C. neoformas and C. gattii strains. C. neoformans was grown under host mimicking conditions (PBS + 10% FBS at 37C) for 72 hours prior to chip sequencing whilst C. gattii was grown in YPD liquid culture at 30°C overnight prior to chip sequencing. The revised manuscript focuses exclusively on C. neoformans H1 paralog function, eliminating the confounding cross-species comparison with C. gattii that concerned the reviewer. IGV genome browser tracks now show ChIP-seq reads along the genome and are included in Figure 5(a) and 5(b). Analysis now focuses comparison of parental KN99a control strain to the linker histone knockout (ΔH1.52) and complemented (ΔH1.52:H1.52) strains, rather than cross species comparisons of H3K4me2 and H3K9me2.

3. Quantification of the C. neoformans called peaks revealed 5x more H3K4me2 peaks and 5x less H3K9me3 peaks in the H1.5 mutant (line 292). Here, I suggest a simple Western blot to visualize this and help verify the peak calling method. Additionally, this result does not seem to match the gene expression data of WT vs. mutant, where there were only very few DEGs?

In the original manuscript, the H1 deletion strains (which were constructed in the KN99α parental background) were compared to KN99_lab as the experimental control for differential gene expression and ChIP-seq peak calling analyses. However, due to laboratory error the sequenced KN99_lab strain was not the actual parental strain from which the deletions were derived. This introduced confounding genetic variation between the control and experimental strains, making it impossible to determine whether observed differences resulted from H1 deletion or from pre-existing genomic differences between KN99_lab and the true parental strain KN99α. In the revised analysis, we re-sequenced (DNA, RNA Chip-seq) the correct KN99a background control. In comparison to the true background control strain, we found that H1.52Δ exhibits dysregulation of 1,561 genes representing 23% of the transcriptome, which is a finding much more in line with the observed enhancement of H3K4me2 peaks and reduction in H3K9me3 peaks observed in this knockout strain.

While we have not added Western blots for total H3K4me2/H3K9me2 levels, we instead generated a complemented strain whose data provides superior genetic validation of the ChIP-seq findings. H1.52:H1.52Δ complementation restored the dispersed H3K9me2 pattern that was lost in H1.52Δ (lines 274-278), demonstrating that the ChIP-seq peak changes are specifically caused by H1.52 loss rather than technical artifacts. Additionally, the mechanistic interpretation is important here. We demonstrate that H1.52 mediates deposition of facultative H3K9me2 domains throughout the genome. Loss of H1.52 doesn’t necessarily

decrease total H3K9me2 protein levels, which is what Western blotting would measure. Instead, it reorganizes H3K9me2 distribution from numerous small, dispersed facultative heterochromatin domains over gene bodies, to fewer, larger constitutive domains (lines 263 – 272). Whilst ChIP-seq can reveal this reorganization, Western blotting would not be able to detect this redistribution, making the complementation-validated ChIP-seq data the more appropriate and informative assay.

4. Lines 299 and 355: It is quite surprising that H3K4me2 is enriched at sub telomeres and centromeres in C. neoformans. Does this in any way match the gene expression data, i.e., are these regions expressed under the cultivation conditions chosen?

The revised manuscript no longer compares C. neoformans and C. gattii H3K4me2 enrichment at sub telomeres vs centromeres and is instead refocused purely on the effect of H1.52 paralog knockout. The section on H3K4me2 enrichment at sub telomeres and centromeres has been fully redacted.

Reviewers' comments:

Reviewer #1 (Remarks to the Author):

Major Comments

1. Line 130-146, some information about the tools should be added for a more general audience. Alternatively, could this be simplified somehow (e.g., some parameters moved to the methods?). Similarly, Line 148-152 should be reworded. This is a key conclusion but is currently difficult to understand. In general, the manuscript would benefit from clearer summary sentences at the end of the results subsections.

We thank the reviewer for their comments. In response, we have made the following changes to this section. The molecular evolution results paragraph has been condensed. Granular details of individual codon level statistics (specific ω values per branch, individual MEME codon positions, and branch-specific aBSREL results) have been summarised to improve readability. Information on tools for this evolutionary analysis are provided in the methods section lines 636 – 667. The concluding sentences for result subsection have been rewritten to more clearly summarise the key biological findings. Specifically, lines 144–148 have been reworded to read: "Together, these analyses support functional divergence following duplication: H1.52 has retained the ancestral linker histone role under strong purifying selection, whilst H1.51 has undergone adaptive refinement at specific residues, particularly in clinical isolates. This is consistent with subfunctionalization of an ancestral H1, with evidence of ongoing neofunctionalization of H1.51 in pathogenic lineages."

2. Line 179-180, please define host-mimicking stress here. It would also be valuable to state the source of knockouts here (e.g., FGSC). In general, (very) brief descriptions of the method used to construct knockouts and produce complementation strains would also be helpful to readers outside the immediate field in assessing the experiments.

We have revised lines 180–187 to explicitly define host-mimicking conditions (PBS supplemented with 10% heat-inactivated FBS, incubated at 37 °C in 5% CO_2). We also now state that the ΔH1.51 and ΔH1.52 strains were obtained from the Madhani deletion collection (Fungal Genetics Stock Centre) and we briefly describe the genetic strategies used, including targeted gene replacement with a nourseothricin resistance cassette and CRISPR mediated reintegration of wild type alleles at the Safe Haven 4 locus for complementation.

3. Line 214, this header is not supported by the data, as no assessment of ribosome

biogenesis itself or translational output was performed. It should be reworded to more accurately reflect the data presented.

We agree that our original header overstated the scope of the data. We have revised the section title to more accurately reflect that our conclusions are based on transcriptional and genomic analyses rather than direct measurements of ribosome biogenesis or translational output. Specifically, we now state that H1.52 deletion transcriptionally represses ribosome biogenesis pathways.

4. Line 243-252, I believe the authors are suggesting that deletion of H1.52 results in halving of the array and complementation then causes it to expand back to K99 levels? If so, this is not totally clear from the text yet (but is clearer in the discussion). Just a curiosity, how long does this process take (in other studies), and does that timeline fit your experimental situation? Related, there is no figure legend or description of the coloured lines for Figure 4d.

To clarify, we are not suggesting that complementation actively drives rDNA array expansion back to KN99 levels. Rather, our interpretation is that rDNA copy number reduction is a condition dependent consequence of H1.52 deletion, occurring specifically after extended culture under host mimicking conditions (72 hours in PBS supplemented with 10% heat-inactivated foetal bovine serum, 37°C, 5% CO_2). The complemented strain, derived from a cryopreserved H1.52Δ stock that had never been subjected to this stress, retains a wildtype equivalent rDNA array, as confirmed by equivalent normalised read depth of the rDNA array under standard YPD growth. Its stability under host-mimicking conditions therefore reflects genuine rescue of H1.52 dependent protective function, rather than active array re-expansion.

Regarding the timeline, rDNA copy number dynamics have been studied in budding yeast, where array contraction and expansion can occur over tens to hundreds of generations depending on replication stress and silencing factor availability. We are not aware of equivalent kinetic data in *Cryptococcus*, however, our 72-hour host-mimicking condition represents an acute stress rather than long term serial passaging. This is consistent with a stress triggered instability event rather than gradual drift. We have clarified this in the manuscript by adding supplementary figure 4 (Fig. S4) demonstrating equivalent normalised read depth across the rDNA array for all three strains grown under YPD versus the contraction specific event seen in H1.52Δ when grown under host mimicking stress.

We also thank the reviewer for noting the missing figure legend for Figure 4d. We have corrected this oversight and added a clear description of the coloured lines in the figure legend.

Minor Comments

5. Line 29, “host-relevant conditions” could be a bit more specific. Later (Line 91), host-mimicking is used. Line 28-31, recovery to what?

We have revised the abstract to specify the host mimicking conditions used (PBS with 10% FBS, 37 °C, 5% CO_2) (Line 29) and standardised the terminology to use ‘host mimicking’ throughout the manuscript. We have also clarified that growth recovery was measured upon return to YPD media (Line 31), making explicit what was being recovered and under what conditions.

6. The manuscript has a number of minor typos, e.g., Line 48 should be encompass; Line 83, adjust “including including”; Line 206, “were”; Line 280, “heterochromatic”; Line 327, “where siginifigantly” should be “were significantly”; Line 528 and 537, “Safe Heaven 4” should be “haven”. Some more proofreading throughout could be beneficial.

We have carried out additional proofreading to identify additional typos throughout the manuscript, and we have corrected the typos that the reviewer has flagged.

7. The concluding paragraph of the intro on Lines 86-100 could be more impactful. The study is interesting, but this paragraph does not really link the findings to a broader context.

We have rewritten the concluding paragraph of the introduction to more clearly contextualise our findings within the broader framework of chromatin mediated cell fate transitions and fungal pathogenesis. The revised paragraph now opens by highlighting the uniqueness of the *Cryptococcus* H1 duplication across 50 basidiomycete species, summarises the functional divergence between paralogs, and explicitly connects the chromatin and metabolic phenotypes of H1.52 deletion to the broader question of how epigenomic organisation underlies stress adaptation in a major human fungal pathogen lines 88-105.

8. Line 109-112, please provide some information on the qualifications necessary to be considered a paralog.

We have added a definition of paralog qualifications to the results section (Lines 110–112): "Paralogs were defined as genes from the same species sharing an orthogroup, as determined by OrthoFinder using all-vs-all BLAST (E-value ≤ 1×10^{-5}) and MCL clustering.". This clarifies the criteria used to classify paralogous genes across all 50 basidiomycete species in our analysis.

9. Figure 3B-E, the names of genes are very hard to read. Could they be made bigger or changed to gray/black (or removed if unnecessary)?

Gene names have been removed from Figure 3B-E as they were unnecessary. Supplementary data file Table S1 contains gene identifier, function and expression information for all strains.

10. Line 616-621, the authors state that nucleic acid quality was assessed, but no information is provided on the result of this assessment. Is the quality comparable between samples?

Nucleic acid quality was assessed using a Qubit 4.0 fluorometer for quantification and an Agilent TapeStation 4200 for quality profiling. RNA integrity numbers (RIN) and DNA integrity numbers (DIN) were comparable across samples. TapeStation metrics for both RNA and DNA samples have been added to Table S8.

11. The complemented strain is denoted a few different ways in the text. It would be easier for the reader if this was standardized. Similar, on Line 344, are the authors referring to a complemented strain with KN99α:H1.52? This notation seems to be different from previous parts of the text.

We have carried out a review of all the strain nomenclature throughout the manuscript, figures, and figure legends, and have standardised the complemented strain designation to H1.52Δ:H1.52 in all instances. The inconsistent notation on Line 344 and elsewhere has been corrected accordingly.

12. Line 336, "181.4% cell viability", I understand the graph, but it seems odd that cells can be more than 100% viable. Could there be a different way to phrase this (e.g., relative metabolism)?

We agree that the term “181.4% cell viability” could be better phrased. In this context, 100% referred to the XTT reduction signal of the KN99α control strain, not absolute viability. As XTT reduction reflects mitochondrial metabolic activity rather than percent survival, values exceeding 100% indicate increased relative metabolic activity compared to the control, not greater than 100% viability. We have revised the text accordingly. Lines 328–331 now read: “Consistent with the transcriptional profile, XTT assays performed after 72 hours revealed significantly elevated XTT reduction in H1.52Δ cells (1.81-fold relative to KN99α; $p < 0.05$), indicating increased overall metabolic activity in the H1.52 knockout strain”.

13. Line 304, is “metabolic shutdown” the correct term here? I am again not sure the data supports such a strong conclusion.

Our data support transcriptional and chromatin mediated downregulation of growth associated programs and reduced relative metabolic activity in wildtype cells compared to H1.52Δ, rather than a complete shutdown. We have therefore revised the section title and corresponding text to more accurately reflect the data. The section is now titled: “H1.52 Promotes Metabolic Downregulation Under Host-Mimicking Conditions”. The Figure 6 title (Lines 860) has been revised to: “Figure 6. H1.52 Restricts Metabolic Activity During Prolonged Host-Mimicking Stress.” We have also adjusted the text throughout this section to replace “metabolic shutdown” with “metabolic downregulation”.

14. Line 468-470, is a reference missing?

The appropriate reference (Pareek & Kaur, 2024) was included but positioned mid-sentence, which may have made it unclear that it supported the full statement. We have revised the text to move the in-text citation to the end of the sentence so that it clearly supports the entire claim.

15. Lines 466-491, the authors hint at changes in gene expression of relevant modulators resulting from the deletion of H1.52. Might some of the phenotypes attributed to H1.52 then be indirect via changes to proteins like Clr4 or the RNAi components? This could be mentioned as a potential confounding factor of the interpretation. The authors could also consider softening “directly govern” on Lines 501-506.

We agree that changes in expression of epigenetic proteins and RNAi components may contribute to some of the observed phenotypes. However, H1 is a well-established structural component of chromatin whose loss has direct consequences for higher-order

chromatin organisation, and the transcriptional dysregulation of downstream effectors such as Clr4 and RNAi components is most parsimoniously interpreted as a consequence of H1.52 deletion rather than an independent cause. We acknowledge that secondary transcriptional cascades of this kind are inherently difficult to fully disentangle, and we have revised the manuscript to note that some phenotypes may be compounded by indirect effects through downstream regulators. We have also softened the language at Lines 501–506, replacing "directly govern" with "are associated with" to more accurately reflect the correlative nature of our evidence.

Reviewer #2 (Remarks to the Author):

- Line 28: In the fungal field, facultative heterochromatin specifically refers to H3K27-marked heterochromatin, so I'd recommend using another term, such as H3K9-marked heterochromatin islands (see for example doi.org/10.1038/s41586-020-2706-x).

To avoid confusion and better align with established usage, we have replaced the term "facultative heterochromatin" with "H3K9-marked heterochromatin islands" throughout the manuscript. Specifically, this change has been made in lines 28, 33, 97, 272, 279, 306, 473, 475, 487, and 497.

- Line 249: Was the complementation construct used to transform the deletion mutant of H1.52? Are the authors suggesting that rDNA loss occurred in a progressive manner through cultivation of the deletion mutant? This means that part of the phenotype of H1.52 deletion is through modification of another genomic location (rDNA), and additionally it means that wild type, deletion and complementation mutants vary in more than one locus. The description of the deletion phenotype should mention this issue, and the title of this section should be changed to reflect this: "H1.52 Deletion Impairs Ribosome Biogenesis and Translation in C. neoformans".

The complementation construct was transformed directly into the H1.52Δ strain, taken from a cryopreserved stock that had never been subjected to host mimicking conditions. Integration was targeted to a defined haven locus SH4 (CNAG_06526) using a pSH4 backbone and confirmed by PCR generating a 2,084 bp product spanning the upstream genomic region into the construct promoter. The H1.52Δ background of complement candidates was independently confirmed using primers spanning the H1.52 ORF.

We appreciate the reviewer's concern regarding potential rDNA variation between strains, but we do not suggest that rDNA loss occurred as a progressive consequence of deletion strain cultivation. To address this directly, we sequenced the wildtype, H1.52Δ, and complemented strains grown under standard YPD conditions and found no difference in normalised read depth across the rDNA array between strains. The rDNA loss in the H1.52Δ background was observed exclusively after extended growth under host mimicking conditions (72 hours in PBS supplemented with 10% heat-inactivated foetal bovine serum, 37°C, 5% CO_2). Because the complement was generated from a cryopreserved H1.52Δ stock that had never experienced this stress, it was derived from a knockout background with an intact rDNA array, as confirmed by equivalent normalised read depth under YPD. This distinction is important as the rDNA instability we report is a condition dependent phenotype of H1.52 deletion, not a fixed genomic difference that confounds strain comparisons under standard culture, and the complemented strain's rDNA stability under stress therefore reflects genuine rescue of H1.52 dependent protective function rather than an artefact of strain history. We have clarified this in the manuscript (Line 234-239 and 244-250) by adding supplementary figure 4 (Fig.S4.pdf) demonstrating normalised read depth across the rDNA array for all three strains grown under YPD versus host mimicking conditions.

We agree that our original section header "H1.52 Deletion Impairs Ribosome Biogenesis and Translation in *C. neoformans*" overstated the scope of the data. We have revised it to more accurately reflect that our conclusions derive from transcriptional and genomic analyses rather than direct measurements of ribosome biogenesis or translational output. The section is now titled "H1.52 Deletion Alters rDNA Copy Number and Ribosome Biogenesis-Related Transcription in *C. neoformans*".

- Line 286-287: “H3K4me2 expansion” implicates spreading which is misleading. One can assume that de-repression of gene expression goes in line with increased H3K4 methylation. Could the authors correlate their RNA-seq data to the ChIP-seq data and determine how many genes are upregulated in the H1.52 deletion mutant and at the same time gain H3K4 methylation?

We have performed additional analysis to demonstrate that H1.52 loss leads to expansion of existing H3K4me2 peaks in addition to de novo deposition of new peaks. This is now presented as an additional multi-panel figure (Supplementary Figure 6). To determine whether H1.52 restricts lateral spreading of H3K4me2, we quantified normalized H3K4me2 signal within inter-peak intervals (<500 bp gaps between adjacent KN99α control H3K4me2

peaks). In H1.52Δ, these intervals exhibited an 18% increase in H3K4me2 signal relative to WT ($p = 6.5 \times 10^{-34}$; Fig. S6a), while signal at peak summits remained unchanged (<1% difference; Fig. S6b). Gap filling probability was distance-dependent, with >95% of gaps <50 bp filled compared to <5% of gaps >3 kb (Fig. S6c), consistent with lateral encroachment from existing peaks as well as de novo deposition. Genome wide, 40% of H1.52Δ peaks showed evidence of spreading, with 22% broadening beyond KN99α boundaries and 18% representing mergers of ≥2 adjacent WT peaks (Fig. S6d). Merged peaks were 2.5-fold wider than retained peaks (Fig. S6e). Complementation reduced inter-peak H3K4me2 signal gain by 33%, demonstrating partial rescue (Fig. S6a,b). Together, these data indicate that H1.52 constrains H3K4me2 to discrete peak domains by preventing lateral spreading into flanking regions. We have added this result to lines 287 – 299 in the revised manuscript.

We correlated RNA-seq expression data with ChIP-seq profiles across all chromatin categories. Only genes co-marked by both H3K4me2 and H3K9me2 in wildtype KN99α were significantly upregulated upon H1.52 deletion (median logFC +0.25, $p=3.2\times10^{-14}$). Genes marked exclusively by either modification showed no significant expression change (H3K4me2 only $p=0.65$; H3K9me2 only $p=0.14$). Complementation returned co-marked gene expression towards wildtype levels (median logFC −0.057, $p=6.9\times10^{-7}$), demonstrating that H3K4me2 gain alone is not the primary driver of transcriptional change, and that H1.52 selectively maintains repression at promoters co-enriched for both marks. This is consistent with research that implicates H3K4me2 as a marker of transcriptional poising rather than active transcription. We acknowledge that bulk ChIP-seq cannot formally distinguish true bivalency from population level heterogeneity, however, the mark-exclusive transcriptional response and its rescue by complementation suggest that co-marked loci constitute a genuine H1.52-dependent regulatory class, irrespective of whether the underlying chromatin architecture reflects bivalency within individual cells or heterogeneity across the population.

- Line 292: Thank you to responding to my previous comment on sub-telomeric H3K4 methylation, however, I still think this is not fully resolved although the comparison to *C. gatii* was removed. The chromosome-wide view of the H3K4me2 mark on chromosome 2 reveals that substantial H3K4 methylation is present at the sub-telomeres in the wild type. Could the authors show the other chromosomes in the supplementary to see if this is the case consistently? Looking at chromosome-wide H3K4me2 in *C. deuterogatii* (doi.org/10.1371/journal.pgen.1009743), this is not really the case. Could the authors add a track which visualizes gene expression under these conditions, to see if this goes in line with gene activation at subtelomeres?

We recognize the absence of sub telomeric H3K4me2 enrichment in *C. deuterogattii*, which may reflect species specific differences or variations in culture conditions (30°C YPD with shaking for 1 day vs. 37°C PBS + 10% FBS + 5% CO_2 without shaking for 3 days). IP minus Input control tracks have been generated for all chromosomes and are presented in supplementary Fig. S5. Sub-telomeric H3K4me2 enrichment is a consistent feature across all chromosomes in *C. neoformans*. We have added RNA-seq expression tracks alongside the ChIP-seq signal to supplementary Fig. S5.

- Line 489: My apologies, I don't follow this conclusion, since I don't see where it was demonstrated that H3K4 methylation was acquired at downregulated genes in the deletion mutant (see my questions above).

We have completely rewritten the concluding paragraph (lines 550–567) to remove the unsupported claim regarding H3K4me2 acquisition at downregulated genes. The revised conclusion restricts all mechanistic interpretation to findings directly demonstrated in this study, describing lateral spreading of H3K4me2 and loss of H3K9me2-marked islands as features of the chromatin landscape, and confining transcriptional interpretation to the co-marked locus analysis, which demonstrates that chromatin state rather than H3K4me2 gain per se is the determinant of transcriptional outcome.

Reviewers' comments:

Reviewer #1 (Remarks to the Author):

Major Comments

1. Line 130-146, some information about the tools should be added for a more general audience. Alternatively, could this be simplified somehow (e.g., some parameters moved to the methods?). Similarly, Line 148-152 should be reworded. This is a key conclusion but is currently difficult to understand. In general, the manuscript would benefit from clearer summary sentences at the end of the results subsections.

We thank the reviewer for their comments. In response, we have made the following changes to this section. The molecular evolution results paragraph has been condensed. Granular details of individual codon level statistics (specific ω values per branch, individual MEME codon positions, and branch-specific aBSREL results) have been summarised to improve readability. Information on tools for this evolutionary analysis are provided in the methods section lines 636 – 667. The concluding sentences for result subsection have been rewritten to more clearly summarise the key biological findings. Specifically, lines 144–148 have been reworded to read: "Together, these analyses support functional divergence following duplication: H1.52 has retained the ancestral linker histone role under strong purifying selection, whilst H1.51 has undergone adaptive refinement at specific residues, particularly in clinical isolates. This is consistent with subfunctionalization of an ancestral H1, with evidence of ongoing neofunctionalization of H1.51 in pathogenic lineages."

2. Line 179-180, please define host-mimicking stress here. It would also be valuable to state the source of knockouts here (e.g., FGSC). In general, (very) brief descriptions of the method used to construct knockouts and produce complementation strains would also be helpful to readers outside the immediate field in assessing the experiments.

We have revised lines 180–187 to explicitly define host-mimicking conditions (PBS supplemented with 10% heat-inactivated FBS, incubated at 37 °C in 5% CO_2). We also now state that the ∆H1.51 and ∆H1.52 strains were obtained from the Madhani deletion collection (Fungal Genetics Stock Centre) and we briefly describe the genetic strategies used, including targeted gene replacement with a nourseothricin resistance cassette and CRISPR mediated reintegration of wild type alleles at the Safe Haven 4 locus for complementation.

3. Line 214, this header is not supported by the data, as no assessment of ribosome

biogenesis itself or translational output was performed. It should be reworded to more accurately reflect the data presented.

We agree that our original header overstated the scope of the data. We have revised the section title to more accurately reflect that our conclusions are based on transcriptional and genomic analyses rather than direct measurements of ribosome biogenesis or translational output. Specifically, we now state that H1.52 deletion transcriptionally represses ribosome biogenesis pathways.

4. Line 243-252, I believe the authors are suggesting that deletion of H1.52 results in halving of the array and complementation then causes it to expand back to K99 levels? If so, this is not totally clear from the text yet (but is clearer in the discussion). Just a curiosity, how long does this process take (in other studies), and does that timeline fit your experimental situation? Related, there is no figure legend or description of the coloured lines for Figure 4d.

To clarify, we are not suggesting that complementation actively drives rDNA array expansion back to KN99 levels. Rather, our interpretation is that rDNA copy number reduction is a condition dependent consequence of H1.52 deletion, occurring specifically after extended culture under host mimicking conditions (72 hours in PBS supplemented with 10% heat-inactivated foetal bovine serum, 37°C, 5% CO_2). The complemented strain, derived from a cryopreserved H1.52Δ stock that had never been subjected to this stress, retains a wildtype equivalent rDNA array, as confirmed by equivalent normalised read depth of the rDNA array under standard YPD growth. Its stability under host-mimicking conditions therefore reflects genuine rescue of H1.52 dependent protective function, rather than active array re-expansion.

Regarding the timeline, rDNA copy number dynamics have been studied in budding yeast, where array contraction and expansion can occur over tens to hundreds of generations depending on replication stress and silencing factor availability. We are not aware of equivalent kinetic data in *Cryptococcus*, however, our 72-hour host-mimicking condition represents an acute stress rather than long term serial passaging. This is consistent with a stress triggered instability event rather than gradual drift. We have clarified this in the manuscript by adding supplementary figure 4 (Fig. S4) demonstrating equivalent normalised read depth across the rDNA array for all three strains grown under YPD versus the contraction specific event seen in H1.52Δ when grown under host mimicking stress.

We also thank the reviewer for noting the missing figure legend for Figure 4d. We have corrected this oversight and added a clear description of the coloured lines in the figure legend.

Minor Comments

5. Line 29, “host-relevant conditions” could be a bit more specific. Later (Line 91), host-mimicking is used. Line 28-31, recovery to what?

We have revised the abstract to specify the host mimicking conditions used (PBS with 10% FBS, 37 °C, 5% CO_2) (Line 29) and standardised the terminology to use ‘host mimicking’ throughout the manuscript. We have also clarified that growth recovery was measured upon return to YPD media (Line 31), making explicit what was being recovered and under what conditions.

6. The manuscript has a number of minor typos, e.g., Line 48 should be encompass; Line 83, adjust “including including”; Line 206, “were”; Line 280, “heterochromatic”; Line 327, “where siginifigantly” should be “were significantly”; Line 528 and 537, “Safe Heaven 4” should be “haven”. Some more proofreading throughout could be beneficial.

We have carried out additional proofreading to identify additional typos throughout the manuscript, and we have corrected the typos that the reviewer has flagged.

7. The concluding paragraph of the intro on Lines 86-100 could be more impactful. The study is interesting, but this paragraph does not really link the findings to a broader context.

We have rewritten the concluding paragraph of the introduction to more clearly contextualise our findings within the broader framework of chromatin mediated cell fate transitions and fungal pathogenesis. The revised paragraph now opens by highlighting the uniqueness of the *Cryptococcus* H1 duplication across 50 basidiomycete species, summarises the functional divergence between paralogs, and explicitly connects the chromatin and metabolic phenotypes of H1.52 deletion to the broader question of how epigenomic organisation underlies stress adaptation in a major human fungal pathogen lines 88-105.

8. Line 109-112, please provide some information on the qualifications necessary to be considered a paralog.

We have added a definition of paralog qualifications to the results section (Lines 110–112): "Paralogs were defined as genes from the same species sharing an orthogroup, as determined by OrthoFinder using all-vs-all BLAST (E-value ≤ 1×10^{-5}) and MCL clustering.". This clarifies the criteria used to classify paralogous genes across all 50 basidiomycete species in our analysis.

9. Figure 3B-E, the names of genes are very hard to read. Could they be made bigger or changed to gray/black (or removed if unnecessary)?

Gene names have been removed from Figure 3B-E as they were unnecessary. Supplementary data file Table S1 contains gene identifier, function and expression information for all strains.

10. Line 616-621, the authors state that nucleic acid quality was assessed, but no information is provided on the result of this assessment. Is the quality comparable between samples?

Nucleic acid quality was assessed using a Qubit 4.0 fluorometer for quantification and an Agilent TapeStation 4200 for quality profiling. RNA integrity numbers (RIN) and DNA integrity numbers (DIN) were comparable across samples. TapeStation metrics for both RNA and DNA samples have been added to Table S8.

11. The complemented strain is denoted a few different ways in the text. It would be easier for the reader if this was standardized. Similar, on Line 344, are the authors referring to a complemented strain with KN99α:H1.52? This notation seems to be different from previous parts of the text.

We have carried out a review of all the strain nomenclature throughout the manuscript, figures, and figure legends, and have standardised the complemented strain designation to H1.52Δ:H1.52 in all instances. The inconsistent notation on Line 344 and elsewhere has been corrected accordingly.

12. Line 336, "181.4% cell viability", I understand the graph, but it seems odd that cells can be more than 100% viable. Could there be a different way to phrase this (e.g., relative metabolism)?

We agree that the term "181.4% cell viability" could be better phrased. In this context, 100% referred to the XTT reduction signal of the KN99α control strain, not absolute viability. As XTT reduction reflects mitochondrial metabolic activity rather than percent survival, values exceeding 100% indicate increased relative metabolic activity compared to the control, not greater than 100% viability. We have revised the text accordingly. Lines 328–331 now read: "Consistent with the transcriptional profile, XTT assays performed after 72 hours revealed significantly elevated XTT reduction in H1.52Δ cells (1.81-fold relative to KN99α; $p < 0.05$), indicating increased overall metabolic activity in the H1.52 knockout strain".

13. Line 304, is "metabolic shutdown" the correct term here? I am again not sure the data supports such a strong conclusion.

Our data support transcriptional and chromatin mediated downregulation of growth associated programs and reduced relative metabolic activity in wildtype cells compared to H1.52Δ, rather than a complete shutdown. We have therefore revised the section title and corresponding text to more accurately reflect the data. The section is now titled: "H1.52 Promotes Metabolic Downregulation Under Host-Mimicking Conditions". The Figure 6 title (Lines 860) has been revised to: "Figure 6. H1.52 Restricts Metabolic Activity During Prolonged Host-Mimicking Stress." We have also adjusted the text throughout this section to replace "metabolic shutdown" with "metabolic downregulation".

14. Line 468-470, is a reference missing?

The appropriate reference (Pareek & Kaur, 2024) was included but positioned mid-sentence, which may have made it unclear that it supported the full statement. We have revised the text to move the in-text citation to the end of the sentence so that it clearly supports the entire claim.

15. Lines 466-491, the authors hint at changes in gene expression of relevant modulators resulting from the deletion of H1.52. Might some of the phenotypes attributed to H1.52 then be indirect via changes to proteins like Clr4 or the RNAi components? This could be mentioned as a potential confounding factor of the interpretation. The authors could also consider softening "directly govern" on Lines 501-506.

We agree that changes in expression of epigenetic proteins and RNAi components may contribute to some of the observed phenotypes. However, H1 is a well-established structural component of chromatin whose loss has direct consequences for higher-order

chromatin organisation, and the transcriptional dysregulation of downstream effectors such as Clr4 and RNAi components is most parsimoniously interpreted as a consequence of H1.52 deletion rather than an independent cause. We acknowledge that secondary transcriptional cascades of this kind are inherently difficult to fully disentangle, and we have revised the manuscript to note that some phenotypes may be compounded by indirect effects through downstream regulators. We have also softened the language at Lines 501–506, replacing "directly govern" with "are associated with" to more accurately reflect the correlative nature of our evidence.

Reviewer #2 (Remarks to the Author):

- Line 28: In the fungal field, facultative heterochromatin specifically refers to H3K27-marked heterochromatin, so I'd recommend using another term, such as H3K9-marked heterochromatin islands (see for example doi.org/10.1038/s41586-020-2706-x).

To avoid confusion and better align with established usage, we have replaced the term "facultative heterochromatin" with "H3K9-marked heterochromatin islands" throughout the manuscript. Specifically, this change has been made in lines 28, 33, 97, 272, 279, 306, 473, 475, 487, and 497.

- Line 249: Was the complementation construct used to transform the deletion mutant of H1.52? Are the authors suggesting that rDNA loss occurred in a progressive manner through cultivation of the deletion mutant? This means that part of the phenotype of H1.52 deletion is through modification of another genomic location (rDNA), and additionally it means that wild type, deletion and complementation mutants vary in more than one locus. The description of the deletion phenotype should mention this issue, and the title of this section should be changed to reflect this: "H1.52 Deletion Impairs Ribosome Biogenesis and Translation in C. neoformans".

The complementation construct was transformed directly into the H1.52Δ strain, taken from a cryopreserved stock that had never been subjected to host mimicking conditions. Integration was targeted to a defined haven locus SH4 (CNAG_06526) using a pSH4 backbone and confirmed by PCR generating a 2,084 bp product spanning the upstream genomic region into the construct promoter. The H1.52Δ background of complement candidates was independently confirmed using primers spanning the H1.52 ORF.

We appreciate the reviewer's concern regarding potential rDNA variation between strains, but we do not suggest that rDNA loss occurred as a progressive consequence of deletion strain cultivation. To address this directly, we sequenced the wildtype, H1.52Δ, and complemented strains grown under standard YPD conditions and found no difference in normalised read depth across the rDNA array between strains. The rDNA loss in the H1.52Δ background was observed exclusively after extended growth under host mimicking conditions (72 hours in PBS supplemented with 10% heat-inactivated foetal bovine serum, 37°C, 5% CO_2). Because the complement was generated from a cryopreserved H1.52Δ stock that had never experienced this stress, it was derived from a knockout background with an intact rDNA array, as confirmed by equivalent normalised read depth under YPD. This distinction is important as the rDNA instability we report is a condition dependent phenotype of H1.52 deletion, not a fixed genomic difference that confounds strain comparisons under standard culture, and the complemented strain's rDNA stability under stress therefore reflects genuine rescue of H1.52 dependent protective function rather than an artefact of strain history. We have clarified this in the manuscript (Line 234-239 and 244-250) by adding supplementary figure 4 (Fig.S4.pdf) demonstrating normalised read depth across the rDNA array for all three strains grown under YPD versus host mimicking conditions.

We agree that our original section header "H1.52 Deletion Impairs Ribosome Biogenesis and Translation in *C. neoformans*" overstated the scope of the data. We have revised it to more accurately reflect that our conclusions derive from transcriptional and genomic analyses rather than direct measurements of ribosome biogenesis or translational output. The section is now titled "H1.52 Deletion Alters rDNA Copy Number and Ribosome Biogenesis-Related Transcription in *C. neoformans*".

- Line 286-287: "H3K4me2 expansion" implicates spreading which is misleading. One can assume that de-repression of gene expression goes in line with increased H3K4 methylation. Could the authors correlate their RNA-seq data to the ChIP-seq data and determine how many genes are upregulated in the H1.52 deletion mutant and at the same time gain H3K4 methylation?

We have performed additional analysis to demonstrate that H1.52 loss leads to expansion of existing H3K4me2 peaks in addition to de novo deposition of new peaks. This is now presented as an additional multi-panel figure (Supplementary Figure 6). To determine whether H1.52 restricts lateral spreading of H3K4me2, we quantified normalized H3K4me2 signal within inter-peak intervals (<500 bp gaps between adjacent KN99α control H3K4me2

peaks). In H1.52Δ, these intervals exhibited an 18% increase in H3K4me2 signal relative to WT ($p = 6.5 \times 10^{-34}$; Fig. S6a), while signal at peak summits remained unchanged (<1% difference; Fig. S6b). Gap filling probability was distance-dependent, with >95% of gaps <50 bp filled compared to <5% of gaps >3 kb (Fig. S6c), consistent with lateral encroachment from existing peaks as well as de novo deposition. Genome wide, 40% of H1.52Δ peaks showed evidence of spreading, with 22% broadening beyond KN99α boundaries and 18% representing mergers of ≥2 adjacent WT peaks (Fig. S6d). Merged peaks were 2.5-fold wider than retained peaks (Fig. S6e). Complementation reduced inter-peak H3K4me2 signal gain by 33%, demonstrating partial rescue (Fig. S6a,b). Together, these data indicate that H1.52 constrains H3K4me2 to discrete peak domains by preventing lateral spreading into flanking regions. We have added this result to lines 287 – 299 in the revised manuscript.

We correlated RNA-seq expression data with ChIP-seq profiles across all chromatin categories. Only genes co-marked by both H3K4me2 and H3K9me2 in wildtype KN99α were significantly upregulated upon H1.52 deletion (median logFC +0.25, $p=3.2\times10^{-14}$). Genes marked exclusively by either modification showed no significant expression change (H3K4me2 only $p=0.65$; H3K9me2 only $p=0.14$). Complementation returned co-marked gene expression towards wildtype levels (median logFC −0.057, $p=6.9\times10^{-7}$), demonstrating that H3K4me2 gain alone is not the primary driver of transcriptional change, and that H1.52 selectively maintains repression at promoters co-enriched for both marks. This is consistent with research that implicates H3K4me2 as a marker of transcriptional poising rather than active transcription. We acknowledge that bulk ChIP-seq cannot formally distinguish true bivalency from population level heterogeneity, however, the mark-exclusive transcriptional response and its rescue by complementation suggest that co-marked loci constitute a genuine H1.52-dependent regulatory class, irrespective of whether the underlying chromatin architecture reflects bivalency within individual cells or heterogeneity across the population.

- Line 292: Thank you to responding to my previous comment on sub-telomeric H3K4 methylation, however, I still think this is not fully resolved although the comparison to *C. gatii* was removed. The chromosome-wide view of the H3K4me2 mark on chromosome 2 reveals that substantial H3K4 methylation is present at the sub-telomeres in the wild type. Could the authors show the other chromosomes in the supplementary to see if this is the case consistently? Looking at chromosome-wide H3K4me2 in *C. deuterogatii* (doi.org/10.1371/journal.pgen.1009743), this is not really the case. Could the authors add a track which visualizes gene expression under these conditions, to see if this goes in line with gene activation at subtelomeres?

We recognize the absence of sub telomeric H3K4me2 enrichment in *C. deuterogattii*, which may reflect species specific differences or variations in culture conditions (30°C YPD with shaking for 1 day vs. 37°C PBS + 10% FBS + 5% CO_2 without shaking for 3 days). IP minus Input control tracks have been generated for all chromosomes and are presented in supplementary Fig. S5. Sub-telomeric H3K4me2 enrichment is a consistent feature across all chromosomes in *C. neoformans*. We have added RNA-seq expression tracks alongside the ChIP-seq signal to supplementary Fig. S5.

- Line 489: My apologies, I don't follow this conclusion, since I don't see where it was demonstrated that H3K4 methylation was acquired at downregulated genes in the deletion mutant (see my questions above).

We have completely rewritten the concluding paragraph (lines 550–567) to remove the unsupported claim regarding H3K4me2 acquisition at downregulated genes. The revised conclusion restricts all mechanistic interpretation to findings directly demonstrated in this study, describing lateral spreading of H3K4me2 and loss of H3K9me2-marked islands as features of the chromatin landscape, and confining transcriptional interpretation to the co-marked locus analysis, which demonstrates that chromatin state rather than H3K4me2 gain per se is the determinant of transcriptional outcome.
