# Supplementary material for: Neofunctionalization of H1 linker histones drives divergent gene expression and histone methylation in Cryptococcus neoformans
Source: Commun Biol. 2026 Jun 10;9:1232. doi: 10.1038/s42003-026-10432-4 (PMC13594160; doi:10.1038/s42003-026-10432-4)
Supplement: Supplementary file 3 [file 42003_2026_10432_MOESM2_ESM.pdf]

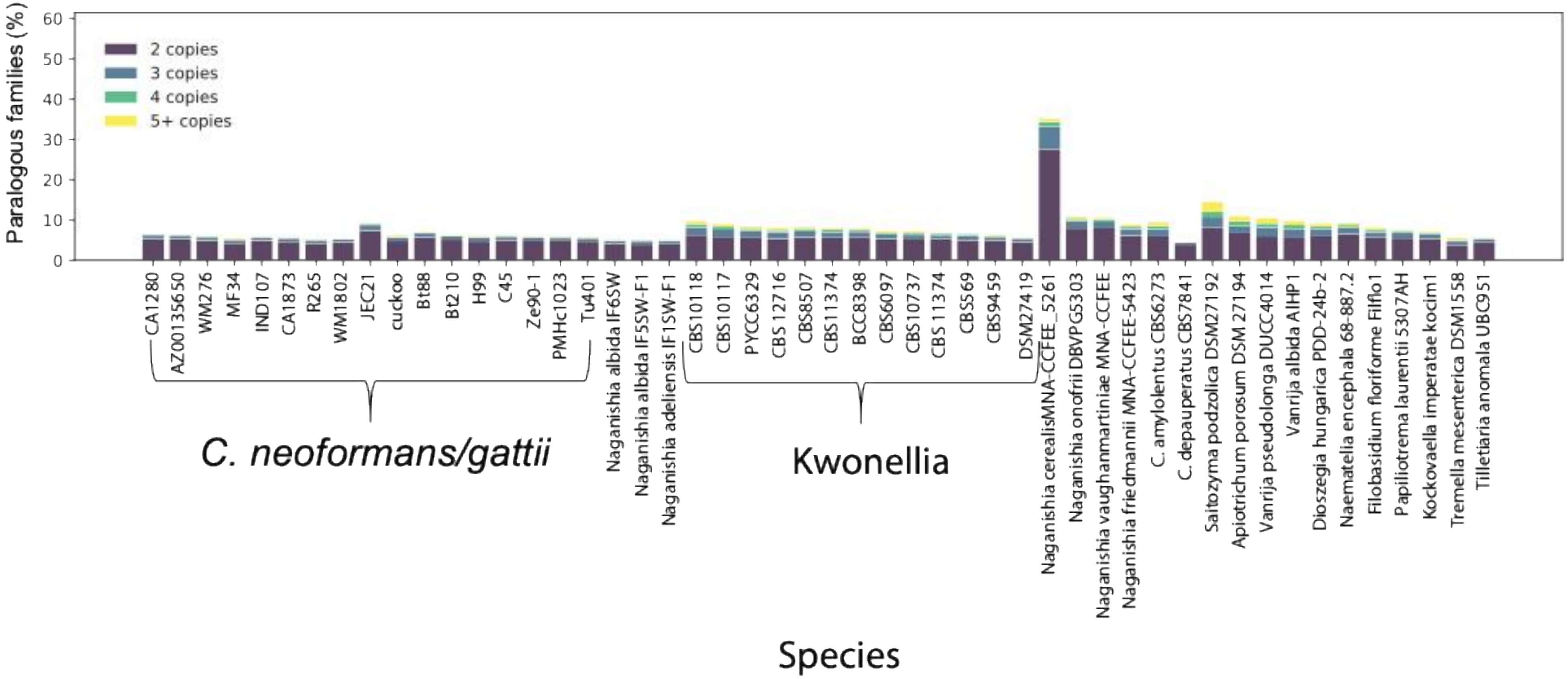

Figure S1. Genome-wide distribution of paralogous gene family sizes across basidiomycete yeasts. Proportion of genes assigned to paralogous gene families originating from gene duplication events across representative basidiomycete yeasts. Paralogous family sizes are colour-coded: purple, two copies; blue, three copies; green, four copies; and yellow, five or more copies. The *Cryptococcus* species complex exhibits a lower proportion of genes in paralogous families (12%) relative to the basidiomycete average (21%).

Analysis	Software	Model	Foreground	lnL	np	ω or K	Comparison	LRT	p-value
Branch model	CODEML (PAML v4.10)	M0 (null: single ω)	—	−1702.877	67	$\omega = 0.292$	—	—	—
		Branch (H1.51 foreground)	H1.51 (CNAG_02924)	−1700.450	68	$\omega_0 = 0.231$ (H1.52) $\omega_1 = 0.499$ (H1.51)	vs M0 null	**4.855**	**0.028**
		Branch (H1.52 foreground)	H1.52 (CNAG_04168)	−1702.567	68	$\omega_0 = 0.306$ (H1.51) $\omega_1 = 0.213$ (H1.52)	vs M0 null	0.620	0.431
Selection intensity	HyPhy RELAX	Null (K = 1)	H1.51	−3088.055	84	K = 1 (fixed)	—	—	—
		Alternative (K estimated)	H1.51	−3081.292	85	K = 7.175	vs RELAX null	**13.526**	**2.35×10^{-4}**
Gene-wide positive selection	HyPhy BUSTED	Constrained ($\omega_3 = 1$)	H1.51	−3096.909	—	$\omega_3 \leq 1$ (constrained)	—	—	—
		Unconstrained	H1.51	−3077.047	—	$\omega_1 = 0.365$ (91.7%) $\omega_2 = 0.000$ (6.8%) $\omega_3 = 2709$ (1.5%)	vs constrained	**39.725**	**1.18×10^{-9}**
Branch-specific positive selection	HyPhy aBSREL	Adaptive (per branch)	H1.51 branches	—	—	3/17 branches significant	—	—	—

Table S1. Summary of molecular evolution analyses for H1 linker histone paralogs in *Cryptococcus*. Analyses performed on a codon alignment of 34 *Cryptococcus* H1 sequences (227 codons, 145 parsimony-informative sites). Bold p-values indicate statistical significance ($p < 0.05$).

Gene ID	Species	Strain	Mol. type	Paralog	H99 ortholog	Rate classes	ω distribution	Uncorr. p	Corr. p
**CNBt88_004709-T1**	***C. neoformans***	**Bt88**	**VNB**	**H1.51**	**CNAG_02924**	**2**	**ω_1 = 0.206 (88.7%); ω_2 = 489.5 (11.3%)**	**8.44×10^{-15}**	**1.43×10^{-13}**
**CNBt210_004656-T1**	***C. neoformans***	**Bt210**	**VNB**	**H1.51**	**CNAG_02924**	**2**	**ω_1 = 0.000 (97.9%); $\omega_2 > 10^4$ (2.1%)**	**4.19×10^{-6}**	**6.71×10^{-5}**
**7000010362876734**	***C. bacillisporus***	**CA1873**	**VGIII**	**H1.51**	**CNAG_02924**	**2**	**ω_1 = 0.000 (99.4%); $\omega_2 > 10^4$ (0.6%)**	**2.62×10^{-5}**	**3.93×10^{-4}**
XXXX_004063T0	*C. gattii s.s.*	MF34	VGI	H1.51	CNAG_02924	2	ω_1 = 0.000 (95.0%); ω_2 = 21.3 (5.0%)	0.024	0.336
CNC05330.t01	*C. deneoformans*	JEC21	—	H1.51	CNAG_02924	1	ω = 0.429 (100%)	—	1.000
CNB3_000617	*C. deuterogattii*	R265	VGII	H1.51	CNAG_02924	1	ω = 0.466 (100%)	—	1.000
I312_102223	*C. bacillisporus*	CA1280	VGIII	H1.51	CNAG_02924	1	ω = 0.419 (100%)	—	1.000
7000010363454155	*C. tetragattii*	IND107	VGIV	H1.51	CNAG_02924	1	ω = 0.250 (100%)	—	1.000
7000006584194326	*C. gattii s.s.*	WM276	VGI	H1.51	CNAG_02924	1	ω = 0.239 (100%)	—	1.000
CDWM_003692T0	*C. gattii*	WM1802	VGVI	H1.51	CNAG_02924	1	ω = 0.000 (100%)	—	1.000
IAS62_000996	*C. decagattii*	7685027	—	H1.51	CNAG_02924	1	ω = 1.000 (100%)	—	1.000
CNAG_02924	*C. neoformans*	H99	VNI	H1.51	CNAG_02924	1	ω = 1.000 (100%)	—	1.000
C356_01663	*C. neoformans*	C45	VNII	H1.51	CNAG_02924	1	ω = 1.000 (100%)	—	1.000
C366_01641	*C. neoformans*	Tu401	VNI	H1.51	CNAG_02924	1	ω = 0.000 (100%)	—	1.000
C367_01665	*C. neoformans*	Ze90-1	VNIa	H1.51	CNAG_02924	1	ω = 1.000 (100%)	—	1.000
FUN_000947	*C. neoformans*	PMHc1023	—	H1.51	CNAG_02924	1	ω = 1.000 (100%)	—	1.000
LQV05_002620	*C. neoformans*	VNII	VNII	H1.51	CNAG_02924	1	ω = 1.000 (100%)	—	1.000

Table S2. Individual H1.51 branch results from aBSREL analysis. Branches tested for episodic diversifying selection with Holm–Bonferroni correction. Bold rows indicate corrected $p < 0.05$.

Codon	α (syn)	β^- (constr.)	p^- (prop.)	β^+ (positive)	p^+ (prop.)	LRT	MEME p	Branches	FEL p	Cross-valid.
2	2.067	0.000	0.817	18,734.7	0.183	13.843	4.21×10^{-4}	1	0.633	No
4	2.052	0.000	0.778	119.6	0.222	5.732	0.026	1	0.965	No
45	0.000	0.000	0.000	1,254.7	1.000	11.254	0.002	1	0.633	No
**116**	**2.297**	**0.000**	**0.000**	**90.2**	**1.000**	**7.317**	**0.012**	**0**	**0.046**	**Yes**
**152**	**0.000**	**0.000**	**0.000**	**10.3**	**1.000**	**5.022**	**0.037**	**1**	**0.025**	**Yes**
178	0.040	0.024	0.279	447.7	0.721	5.661	0.027	2	0.424	No
186	2.081	1.005	0.704	20,705.7	0.296	21.503	9.00×10^{-6}	1	0.930	No
145	—	—	—	—	—	—	n.s.	—	0.074	FEL only

Table S3. Codons under diversifying selection in H1 linker histones. MEME detects episodic diversifying selection ($p < 0.05$); FEL detects pervasive diversifying selection ($p < 0.1$). Alignment of 34 *Cryptococcus* sequences, 227 codons. Bold rows indicate sites cross-validated by both methods. α = synonymous substitution rate. β^- = non-synonymous rate for the constrained proportion. β^+ = non-synonymous rate for the positively selected proportion. p^- and p^+ = proportion of branches in constrained and positive selection classes, respectively. Branches = number of branches at which episodic selection was inferred. Cross-validated = significant in both MEME (episodic, $p < 0.05$) and FEL (pervasive, $p < 0.1$). Codon 145 was significant by FEL only ($p = 0.074$).

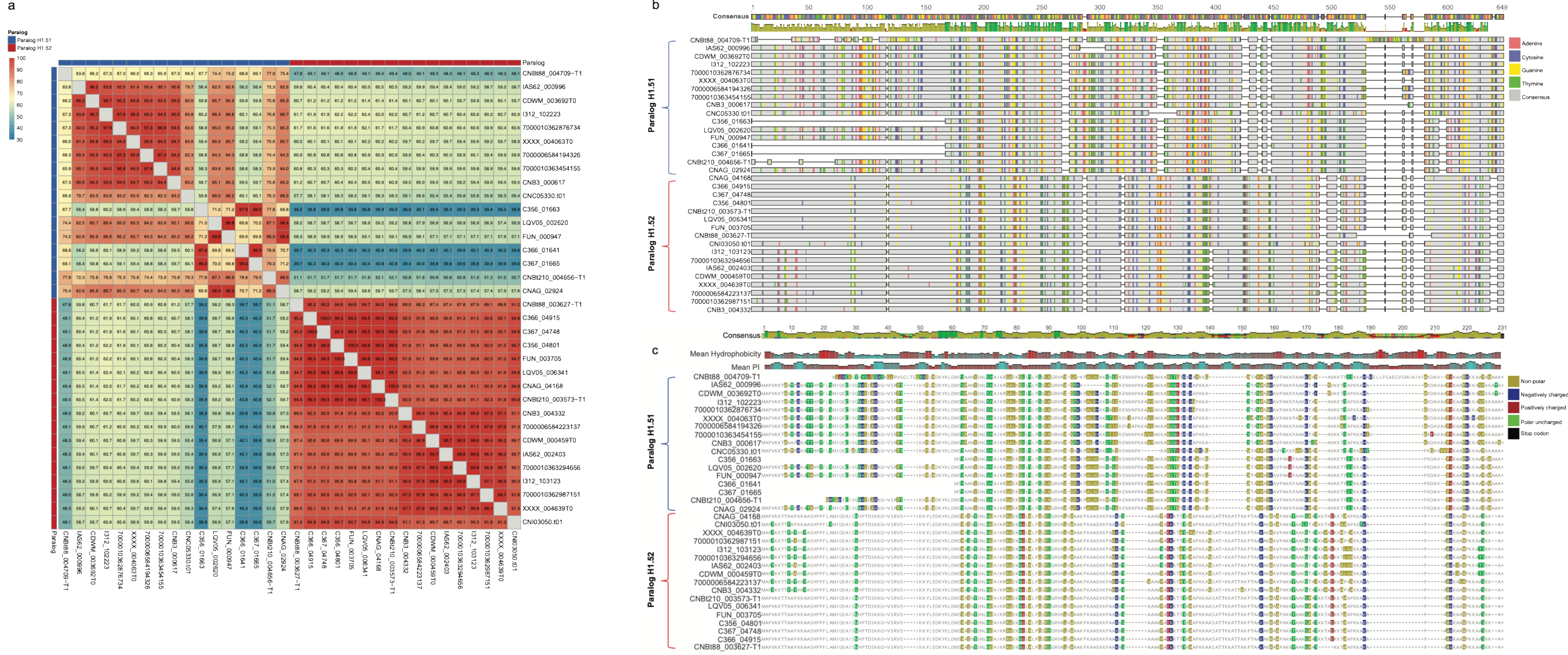

Figure S2. Sequence similarity and alignment analyses of *Cryptococcus* H1 paralog groups. (a) Pairwise percentage similarity matrix of H1 homologs across paralog groups (H1.51 and H1.52). Heat map colors indicate sequence similarity (yellow to red, 35–100%). (b) Nucleotide sequence alignment of 34 H1 genes from 17 Cryptococcus strains. (c) Amino acid sequence alignment. Alignments visualized in Geneious Prime with consensus sequences shown; deviations from consensus are highlighted. Nucleotides are color-coded: adenine (red), cytosine (purple), guanine (yellow), thymine (green), consensus (grey). Amino acids are colored by biochemical property: non-polar/hydrophobic (yellow; A, F, G, I, L, M, P, V, W), polar uncharged (green; C, N, Q, S, T, Y), negatively charged (blue; D, E), positively charged (red; H, K, R).

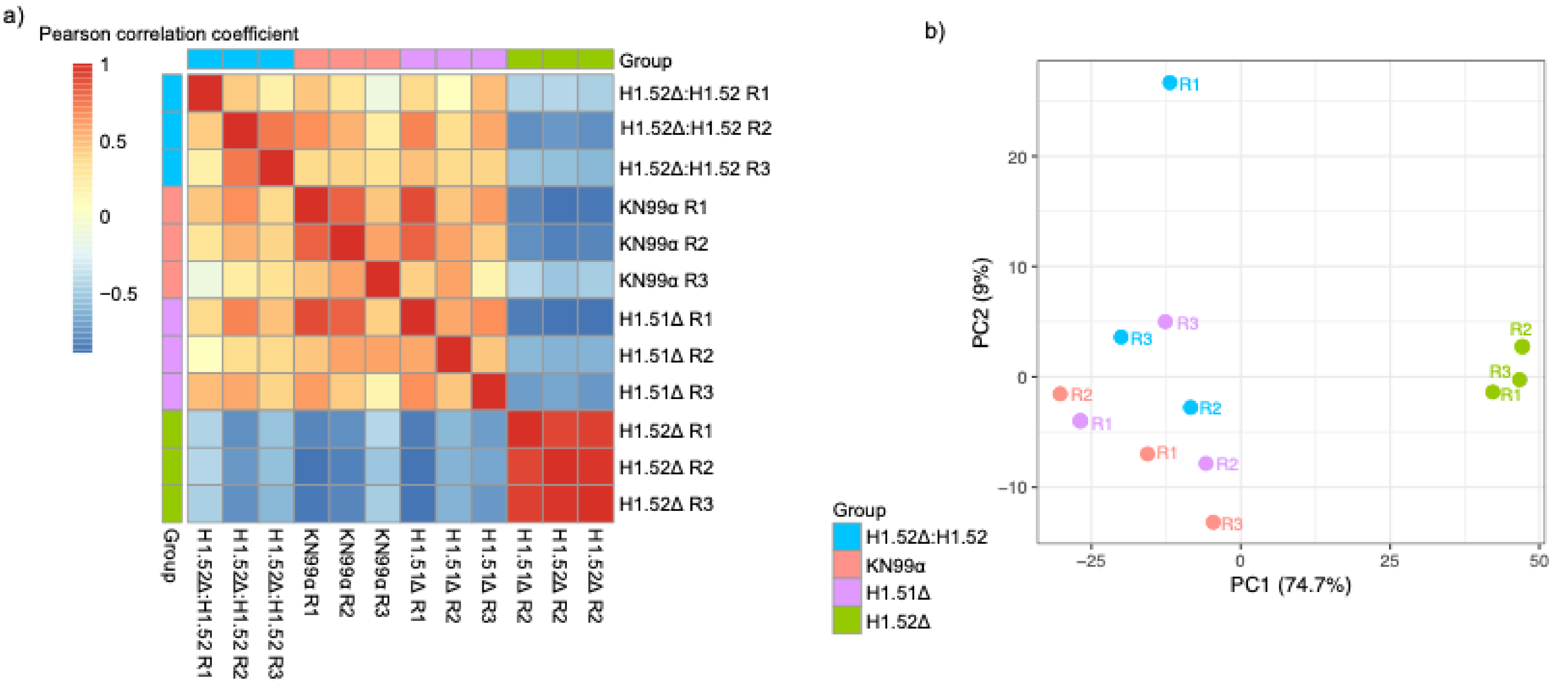

Figure S3. RNA-seq sample replicate correlations and Principal Component Analysis. (a) Pearson correlation heatmap showing pairwise correlations between all samples. Colour scale indicates correlation strength from -0.5 (blue) to 1 (red). Samples cluster by experimental group, with high correlation between biological replicates within each group. (b) Principal component analysis (PCA) of experimental groups and replicates. Samples are plotted along the first two principal components, with PC1 explaining 74.7% and PC2 explaining 5% of the variance. Each experimental group (H1.52:H1.52Δ, KN99α Control, KN99αH1.51Δ, and H1.52Δ) is represented by three biological replicates (R1-R3).

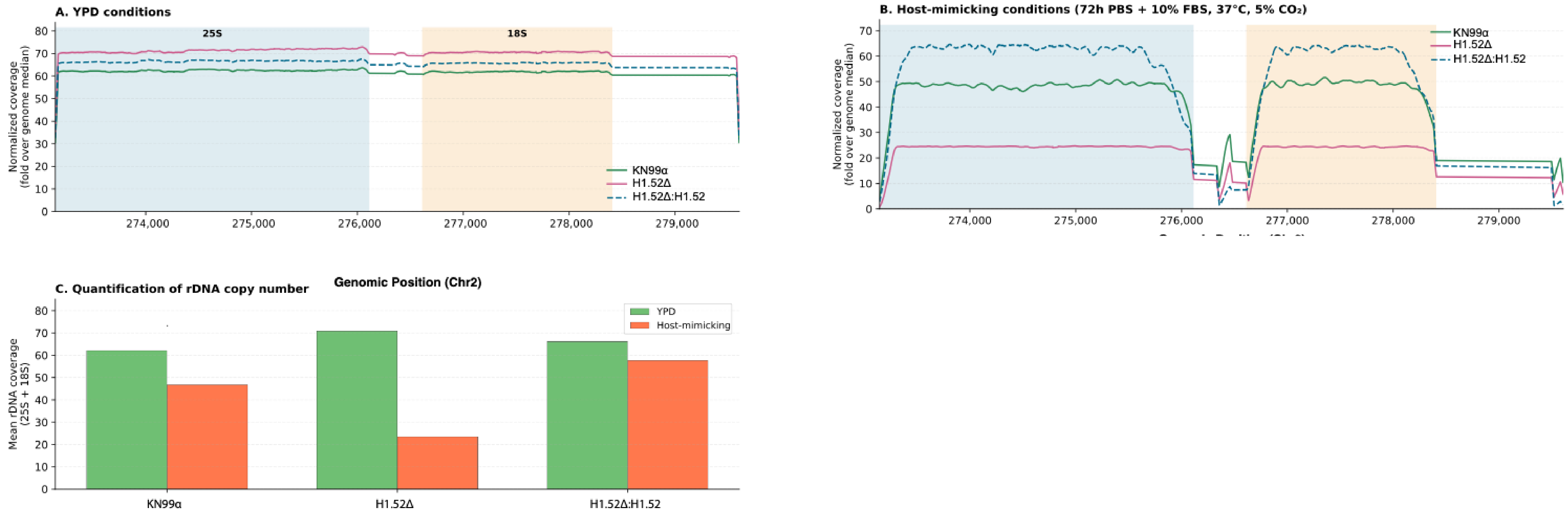

Figure S4. H1.52 is required for rDNA array stability under host-mimicking conditions: (a) Normalized read depth across the 25S and 18S rRNA genes for wildtype (KN99α), H1.52Δ, and complemented strains grown under standard YPD conditions (30°C). Coverage was normalized to genome-wide median depth. No significant difference in rDNA copy number was observed between strains. Data from ONT long-read sequencing aligned to the CNA2 reference genome. (b) Normalized read depth after 72 hours under host-mimicking conditions (PBS supplemented with 10% heat inactivated foetal bovine serum, 37°C, 5% CO_2). H1.52Δ shows marked reduction in rDNA coverage compared to wildtype and complemented strains. Data from Illumina short read sequencing aligned to rDNA gene sequences and normalized to genome wide median depth. (c) Quantification of mean rDNA coverage (25S + 18S genes). Percentages indicate rDNA retention under stress relative to standard culture. H1.52Δ retains only 36% of its rDNA complement under stress compared to 82% in wildtype, that rDNA instability is a condition-dependent phenotype of H1.52 deletion. The complemented strain shows rescued rDNA stability (95% retention), confirming that this phenotype is due to loss of H1.52 function.

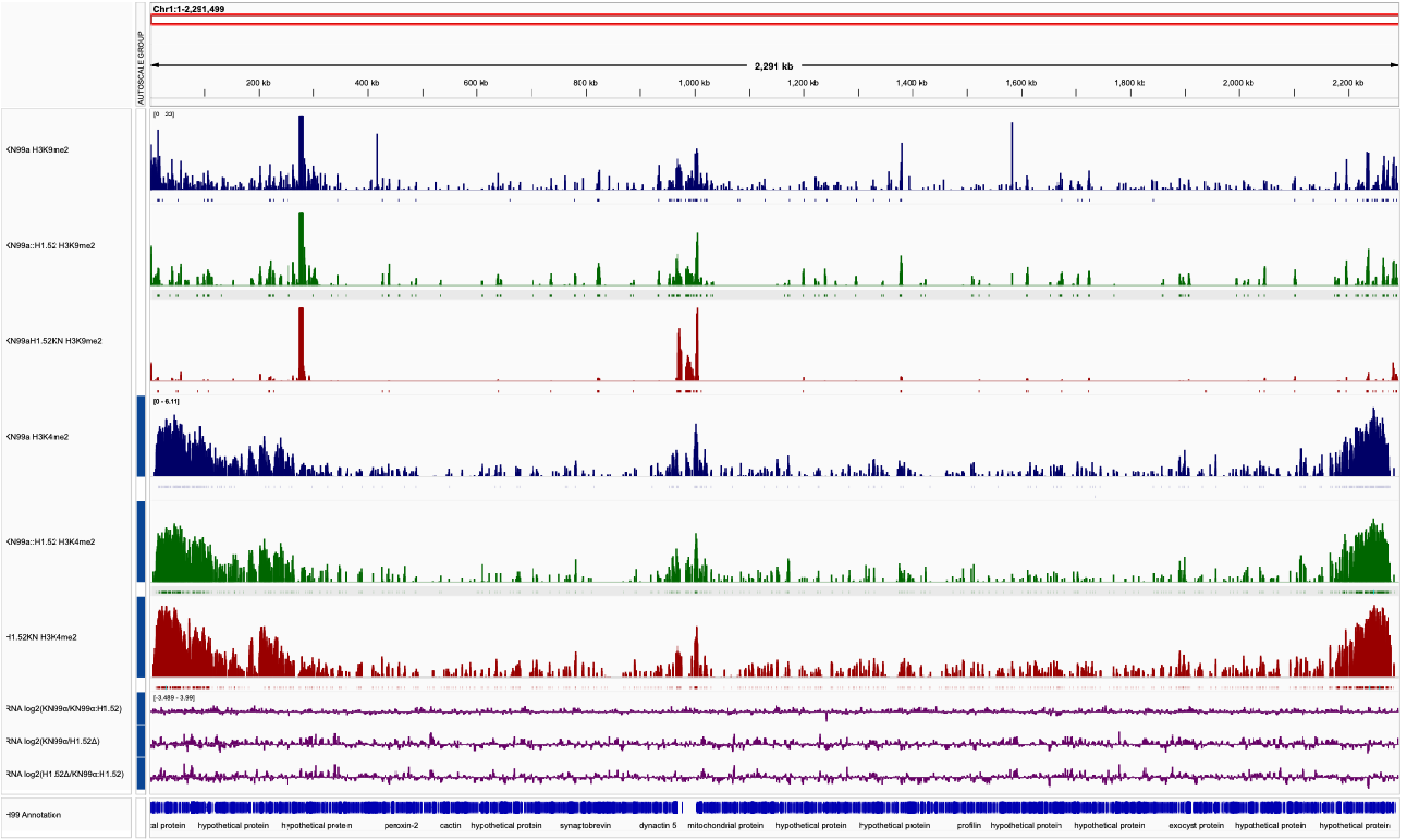

Chr1:1-2,291,499
AUTOSCALE GROUP
2,291 kb
200 kb
400 kb
600 kb
800 kb
1,000 kb
1,200 kb
1,400 kb
1,600 kb
1,800 kb
2,000 kb
2,200 kb
[0 - 22]
KN99a H3K9me2
KN99a::H1.52 H3K9me2
KN99aH1.52KN H3K9me2
[0 - 6.11]
KN99a H3K4me2
KN99a::H1.52 H3K4me2
H1.52KN H3K4me2
[-3.489 - 3.99]
RNA log2(KN99α/KN99α:H1.52)
RNA log2(KN99α/H1.52Δ)
RNA log2(H1.52Δ/KN99α:H1.52)
H99 Annotation
al protein
hypothetical protein
hypothetical protein
peroxin-2
cactin
hypothetical protein
synaptobrevin
dynactin 5
mitochondrial protein
hypothetical protein
hypothetical protein
profilin
hypothetical protein
hypothetical protein
exocyst protein
hypothetical protein
hypothetical protein

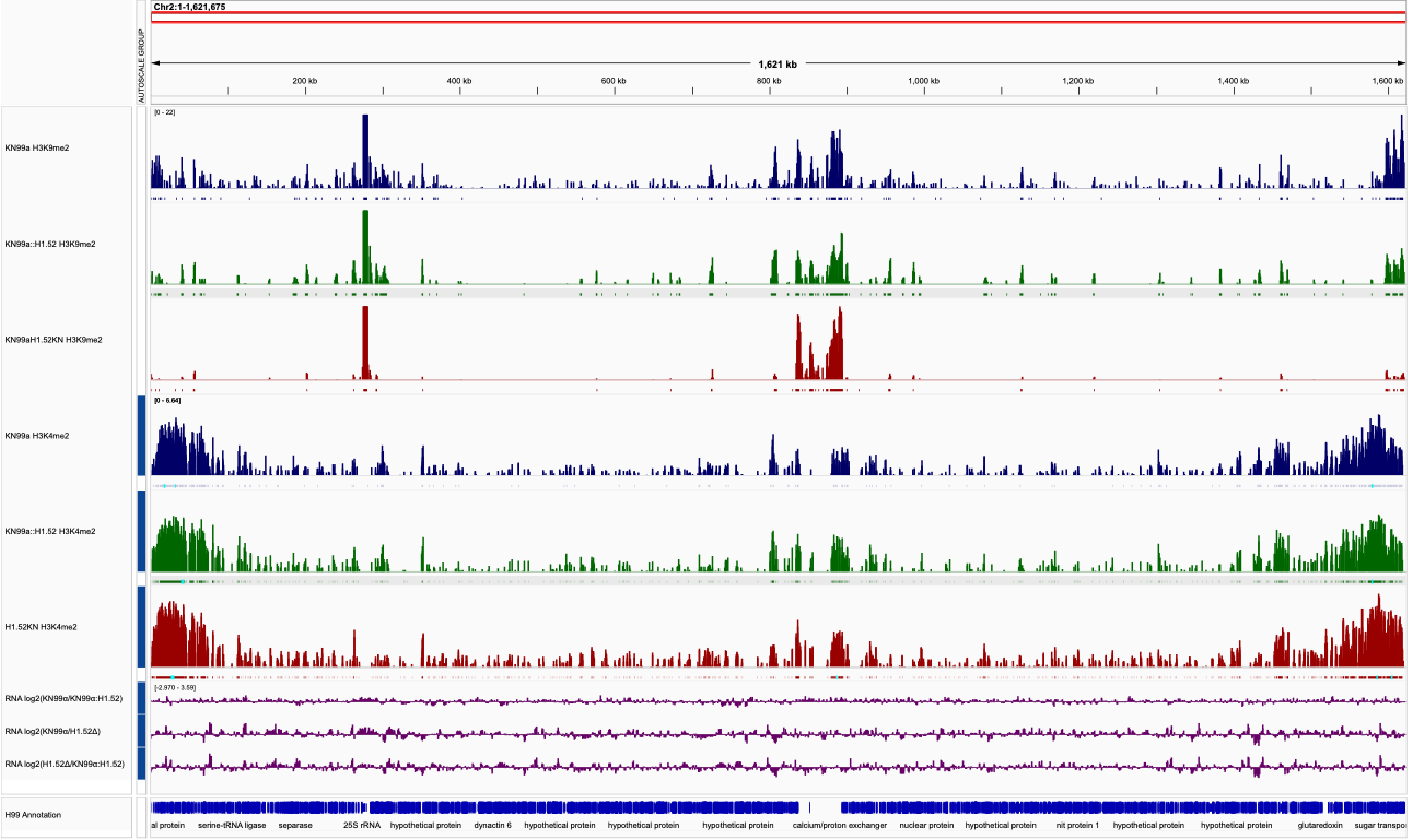

Chr2:1-1,621,675
AUTOSCALE GROUP
1,621 kb
200 kb
400 kb
600 kb
800 kb
1,000 kb
1,200 kb
1,400 kb
1,600 kb
KN99a H3K9me2
[0 - 22]
KN99a::H1.52 H3K9me2
KN99aH1.52KN H3K9me2
KN99a H3K4me2
[0 - 6.64]
KN99a::H1.52 H3K4me2
H1.52KN H3K4me2
RNA log2(KN99α/KN99α:H1.52)
[-2.970 - 3.59]
RNA log2(KN99α/H1.52Δ)
RNA log2(H1.52Δ/KN99α:H1.52)
H99 Annotation
al protein
serine-tRNA ligase
separase
25S rRNA
hypothetical protein
dynactin 6
hypothetical protein
hypothetical protein
hypothetical protein
calcium/proton exchanger
nuclear protein
hypothetical protein
nit protein 1
hypothetical protein
hypothetical protein
glutaredoxin
sugar transpo

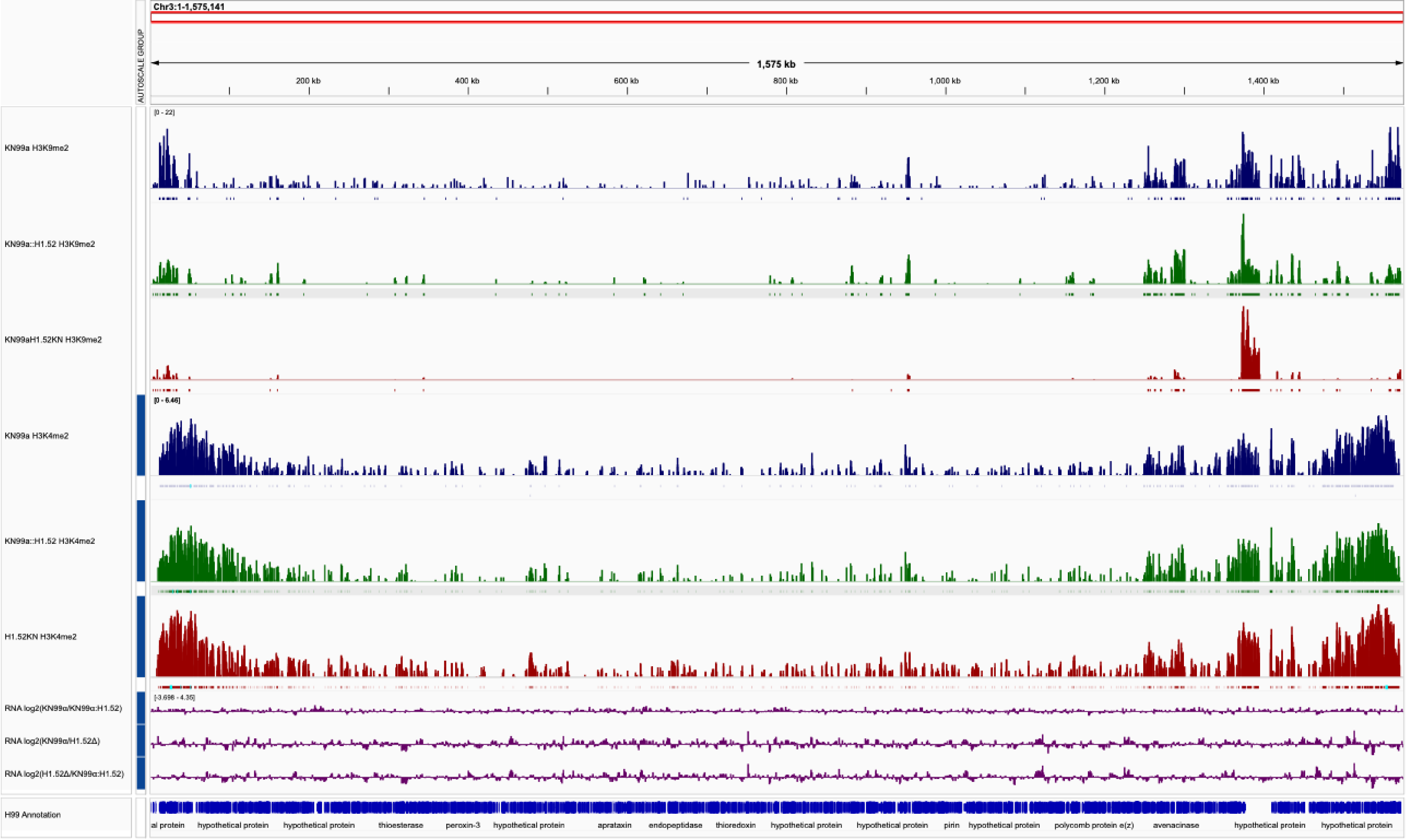

Chr3:1-1,575,141
AUTOSCALE GROUP
1,575 kb
200 kb
400 kb
600 kb
800 kb
1,000 kb
1,200 kb
1,400 kb
[0 - 22]
KN99a H3K9me2
KN99a::H1.52 H3K9me2
KN99aH1.52KN H3K9me2
[0 - 6.46]
KN99a H3K4me2
KN99a::H1.52 H3K4me2
H1.52KN H3K4me2
[-3.696 - 4.35]
RNA log2(KN99α/KN99α:H1.52)
RNA log2(KN99α/H1.52Δ)
RNA log2(H1.52Δ/KN99α:H1.52)
H99 Annotation
al protein
hypothetical protein
hypothetical protein
thioesterase
peroxin-3
hypothetical protein
aprataxin
endopeptidase
thioredoxin
hypothetical protein
hypothetical protein
pirin
hypothetical protein
polycomb protein e(z)
avenacinase
hypothetical protein
hypothetical protein

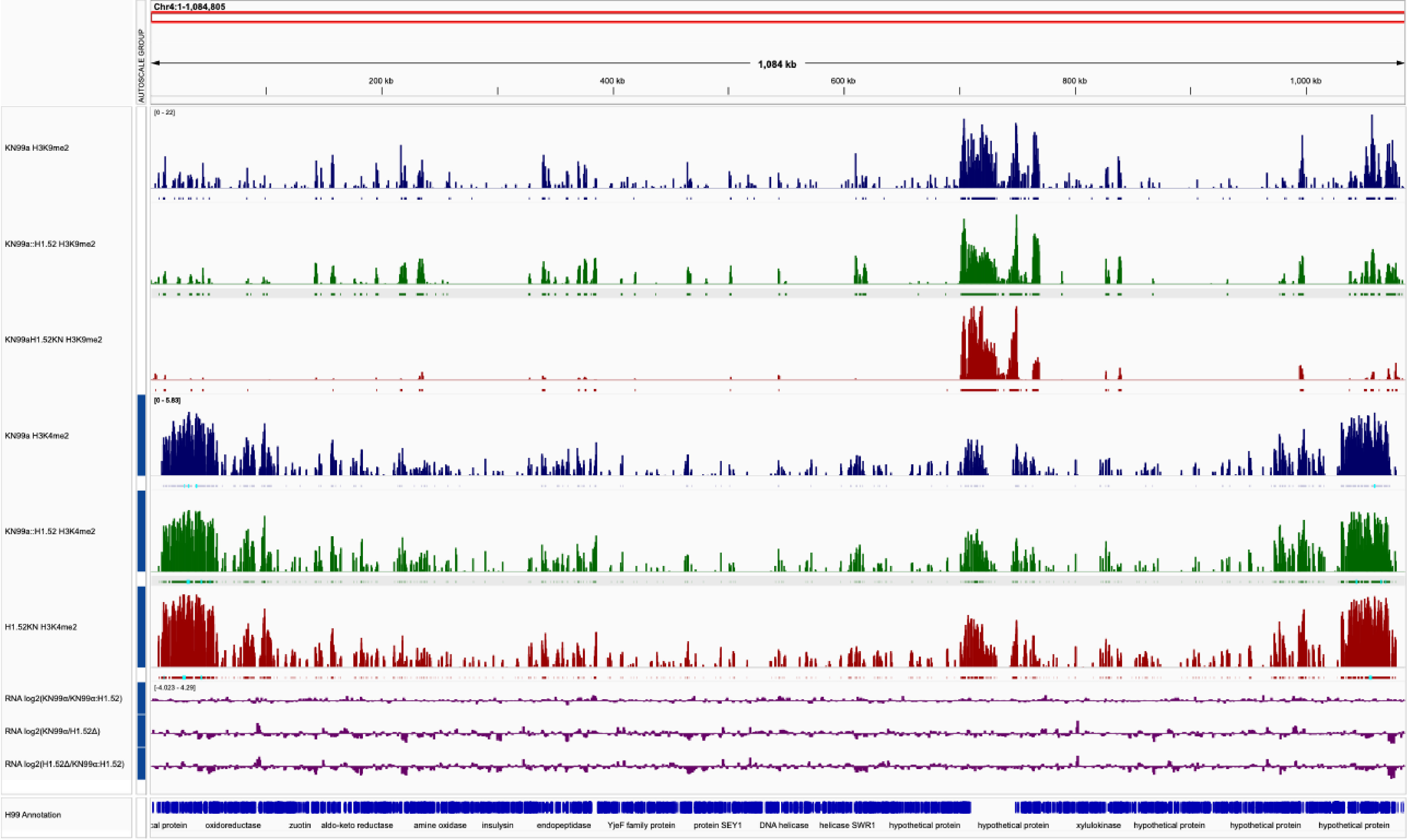

Chr4:1-1,084,805
AUTOSCALE GROUP
1,084 kb
200 kb
400 kb
600 kb
800 kb
1,000 kb
[0 - 22]
KN99a H3K9me2
KN99a::H1.52 H3K9me2
KN99aH1.52KN H3K9me2
[0 - 5.83]
KN99a H3K4me2
KN99a::H1.52 H3K4me2
H1.52KN H3K4me2
[-4.023 - 4.29]
RNA log2(KN99α/KN99α:H1.52)
RNA log2(KN99α/H1.52Δ)
RNA log2(H1.52Δ/KN99α:H1.52)
H99 Annotation
cal protein
oxidoreductase
zuotin
aldo-keto reductase
amine oxidase
insulysin
endopeptidase
YjeF family protein
protein SEY1
DNA helicase
helicase SWR1
hypothetical protein
hypothetical protein
xylulokinase
hypothetical protein
hypothetical protein
hypothetical protein

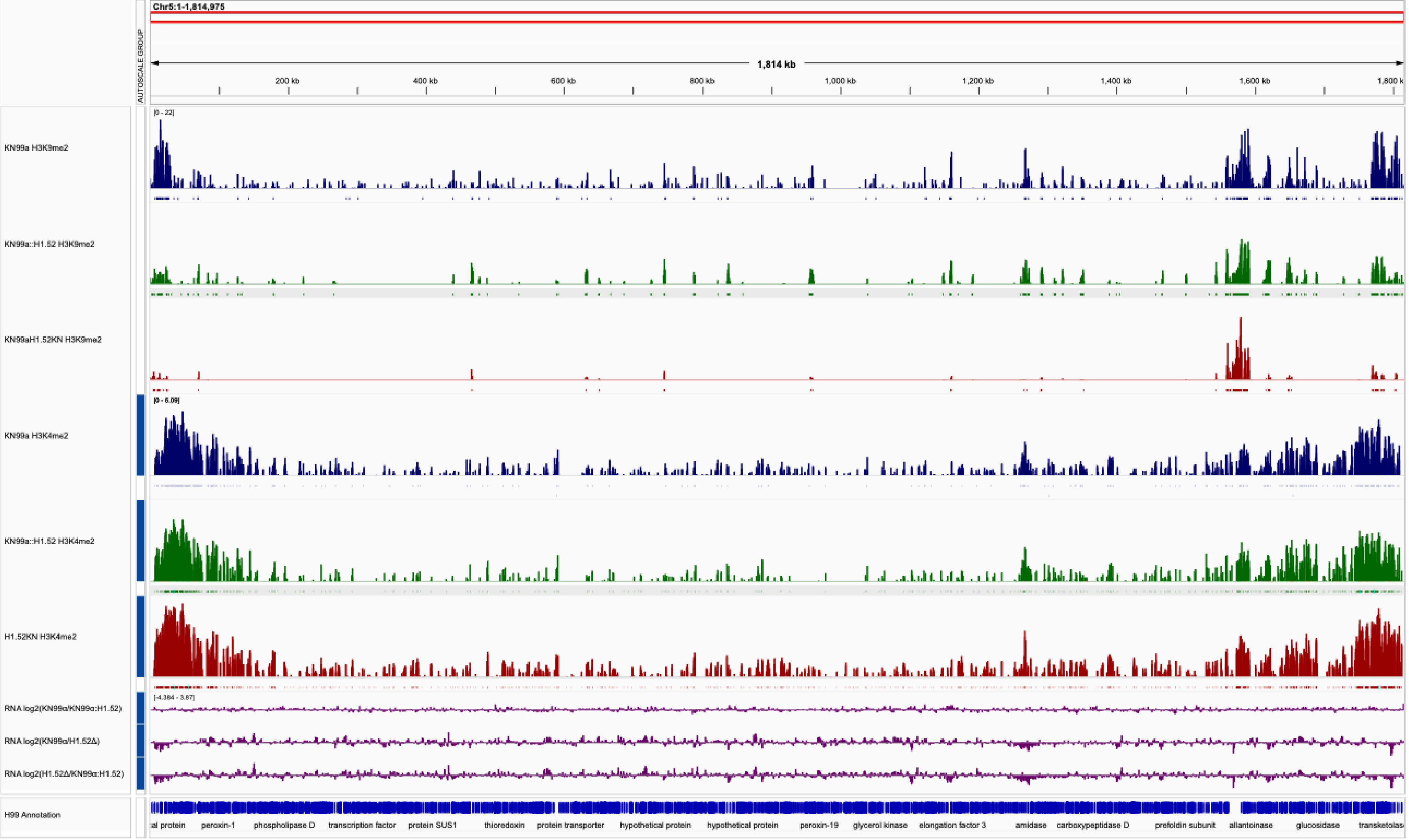

Chr5:1-1,814,975
AUTOSCALE GROUP
1,814 kb
200 kb
400 kb
600 kb
800 kb
1,000 kb
1,200 kb
1,400 kb
1,600 kb
1,800 k
[0 - 22]
KN99a H3K9me2
KN99a::H1.52 H3K9me2
KN99aH1.52KN H3K9me2
[0 - 6.09]
KN99a H3K4me2
KN99a::H1.52 H3K4me2
H1.52KN H3K4me2
[-4.384 - 3.87]
RNA log2(KN99α/KN99α:H1.52)
RNA log2(KN99α/H1.52Δ)
RNA log2(H1.52Δ/KN99α:H1.52)
H99 Annotation
al protein
peroxin-1
phospholipase D
transcription factor
protein SUS1
thioredoxin
protein transporter
hypothetical protein
hypothetical protein
peroxin-19
glycerol kinase
elongation factor 3
amidase
carboxypeptidase D
prefoldin subunit
allantoinase
glucosidase
transketolas

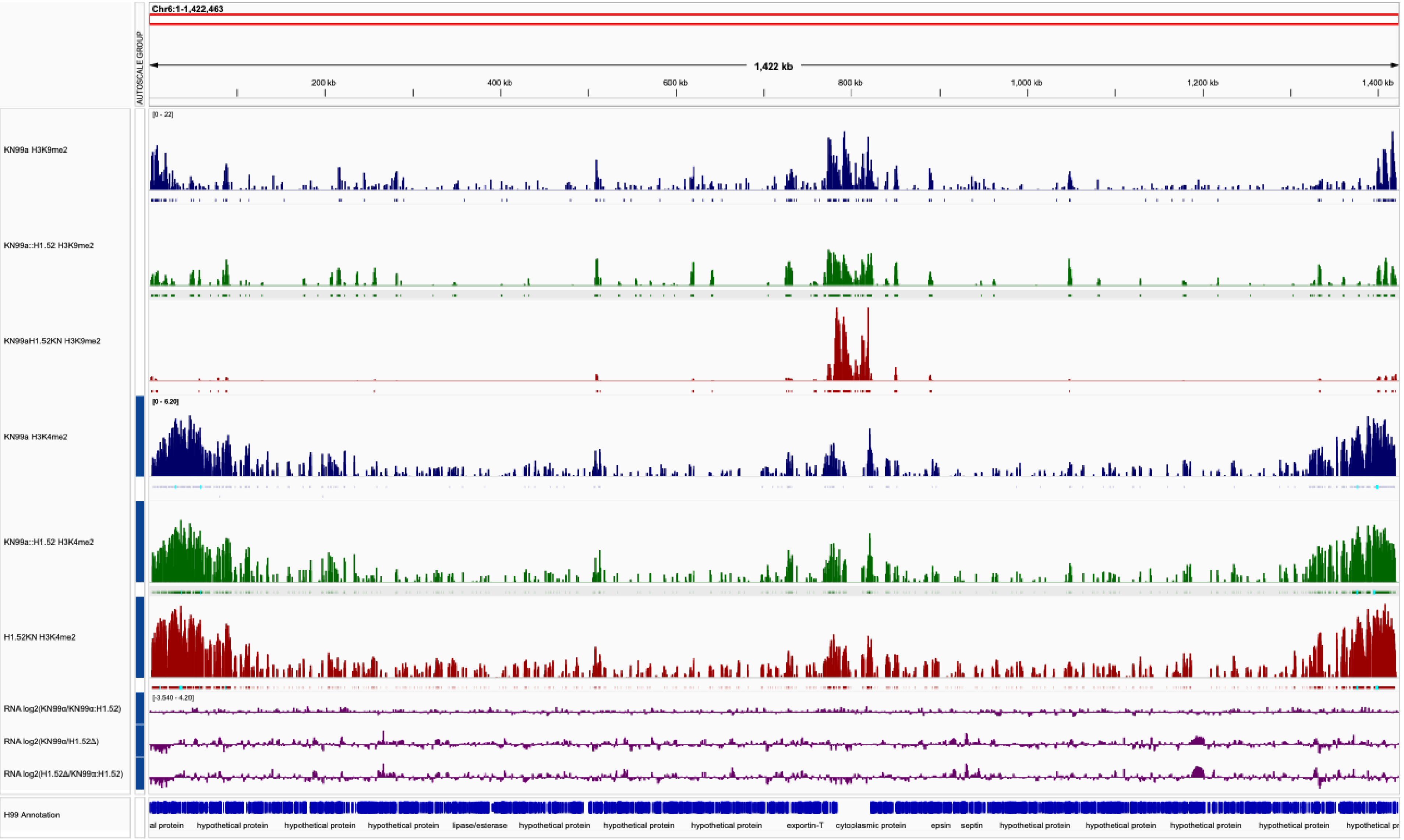

Chr6:1-1,422,463
AUTOSCALE GROUP
1,422 kb
200 kb
400 kb
600 kb
800 kb
1,000 kb
1,200 kb
1,400 kb
[0 - 22]
KN99a H3K9me2
KN99a::H1.52 H3K9me2
KN99aH1.52KN H3K9me2
[0 - 6.20]
KN99a H3K4me2
KN99a::H1.52 H3K4me2
H1.52KN H3K4me2
[-3.540 - 4.20]
RNA log2(KN99α/KN99α:H1.52)
RNA log2(KN99α/H1.52Δ)
RNA log2(H1.52Δ/KN99α:H1.52)
H99 Annotation
al protein
hypothetical protein
hypothetical protein
hypothetical protein
lipase/esterase
hypothetical protein
hypothetical protein
hypothetical protein
exportin-T
cytoplasmic protein
epsin
septin
hypothetical protein
hypothetical protein
hypothetical protein
hypothetical protein
hypothetical pr

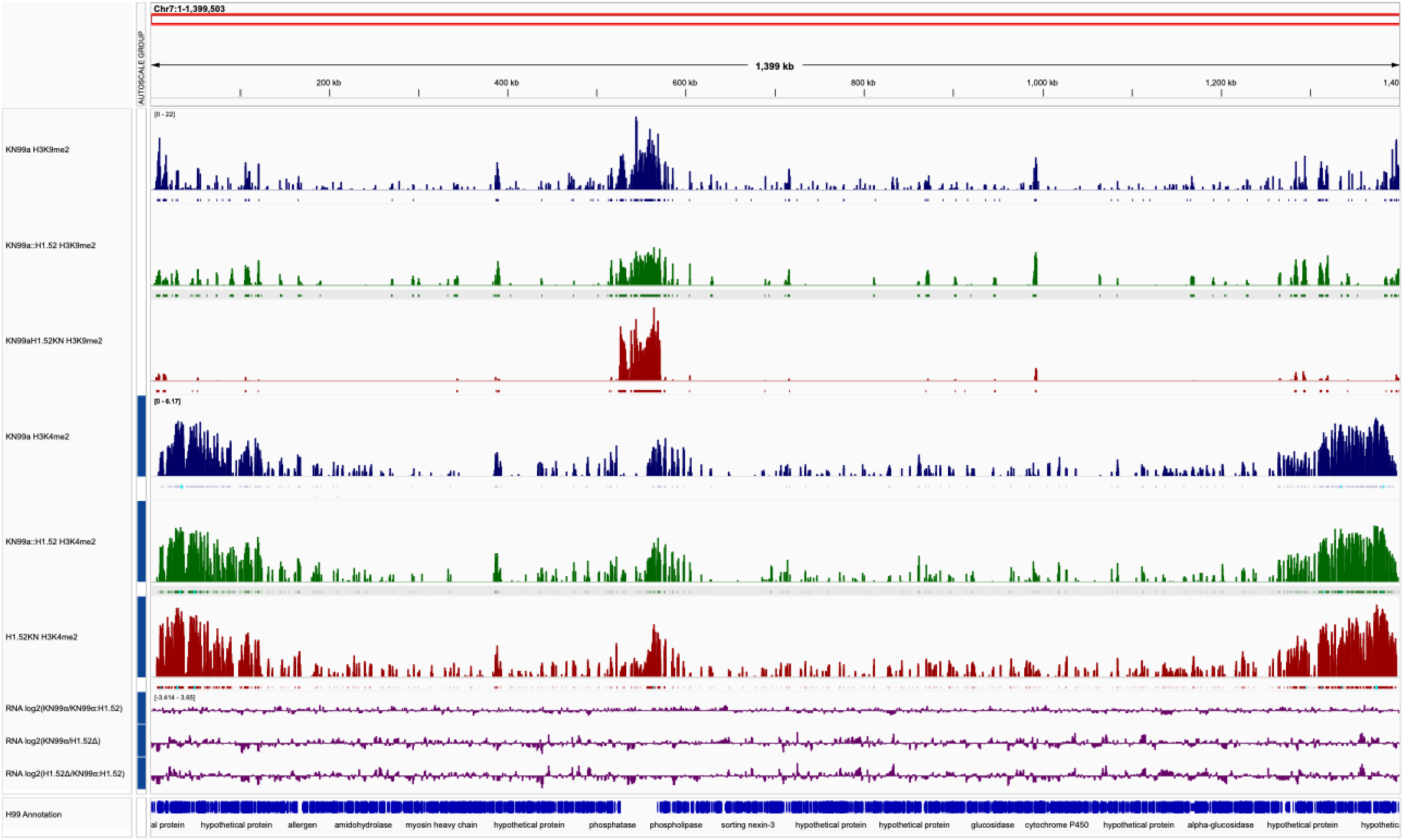

Chr7:1-1,399,503
1,399 kb
200 kb
400 kb
600 kb
800 kb
1,000 kb
1,200 kb
AUTOSCALE GROUP
KN99a H3K9me2
KN99a::H1.52 H3K9me2
KN99aH1.52KN H3K9me2
KN99a H3K4me2
KN99a::H1.52 H3K4me2
H1.52KN H3K4me2
RNA log2(KN99α/KN99α::H1.52)
RNA log2(KN99α/H1.52Δ)
RNA log2(H1.52Δ/KN99α::H1.52)
H99 Annotation
hypothetical protein
allergen
amidohydrolase
myosin heavy chain
phosphatase
phospholipase
sorting nexin-3
glucosidase
cytochrome P450
alpha-glucosidase

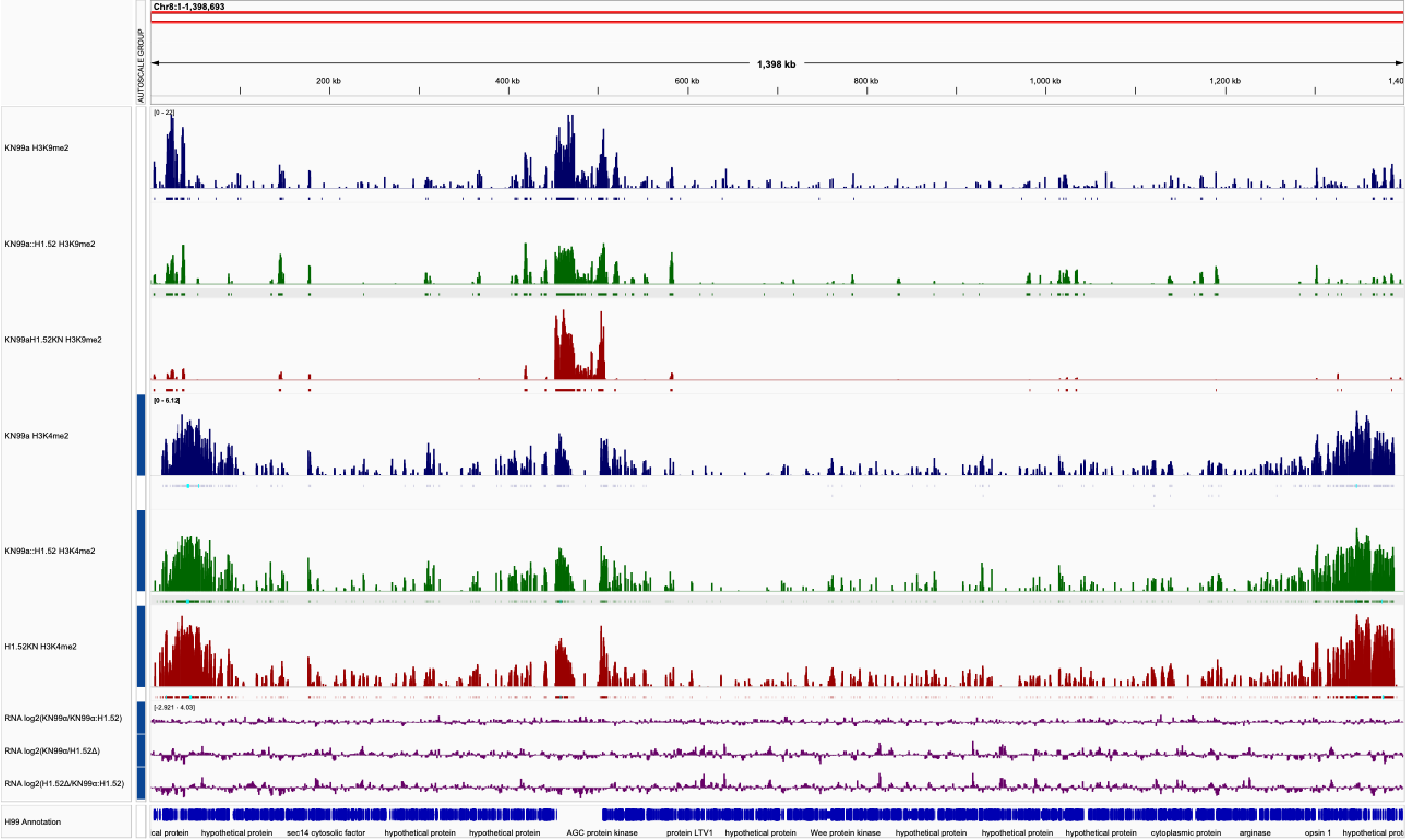
Chr8:1-1,398,693
AUTOSCALE GROUP
1,398 kb
200 kb
400 kb
600 kb
800 kb
1,000 kb
1,200 kb
1,40
[0 - 22]
KN99a H3K9me2
KN99a::H1.52 H3K9me2
KN99aH1.52KN H3K9me2
[0 - 6.12]
KN99a H3K4me2
KN99a::H1.52 H3K4me2
H1.52KN H3K4me2
[-2.921 - 4.03]
RNA log2(KN99α/KN99α:H1.52)
RNA log2(KN99α/H1.52Δ)
RNA log2(H1.52Δ/KN99α:H1.52)
H99 Annotation
cal protein
hypothetical protein
sec14 cytosolic factor
hypothetical protein
hypothetical protein
AGC protein kinase
protein LTV1
hypothetical protein
Wee protein kinase
hypothetical protein
hypothetical protein
hypothetical protein
cytoplasmic protein
arginase
opsin 1
hypothetical prot

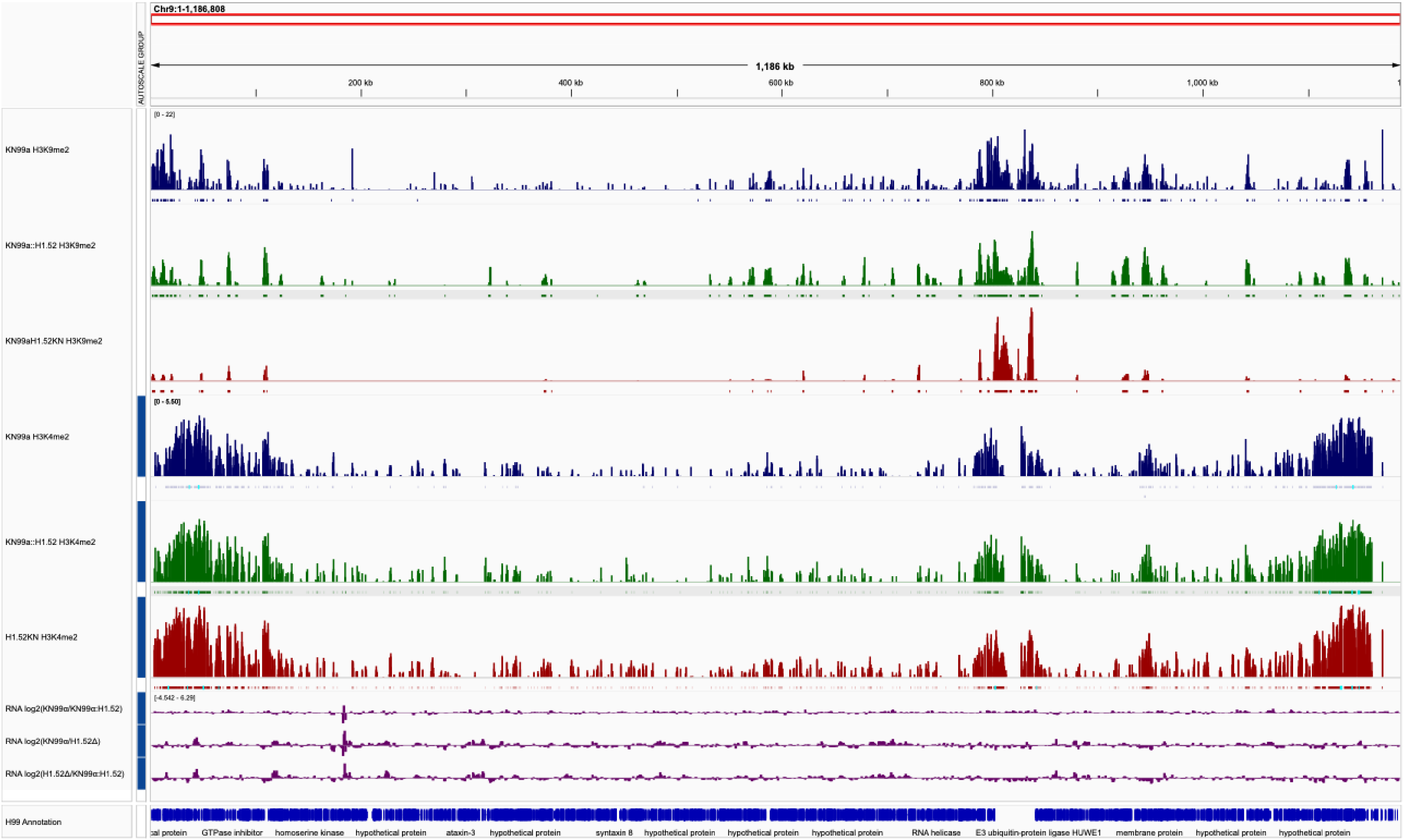

Chr9:1-1,186,808
AUTOSCALE GROUP
1,186 kb
200 kb
400 kb
600 kb
800 kb
1,000 kb
[0 - 22]
KN99a H3K9me2
KN99a::H1.52 H3K9me2
KN99aH1.52KN H3K9me2
[0 - 5.50]
KN99a H3K4me2
KN99a::H1.52 H3K4me2
H1.52KN H3K4me2
[-4.542 - 6.29]
RNA log2(KN99α/KN99α:H1.52)
RNA log2(KN99α/H1.52Δ)
RNA log2(H1.52Δ/KN99α:H1.52)
H99 Annotation
cal protein
GTPase inhibitor
homoserine kinase
hypothetical protein
ataxin-3
hypothetical protein
syntaxin 8
hypothetical protein
hypothetical protein
hypothetical protein
RNA helicase
E3 ubiquitin-protein ligase HUWE1
membrane protein
hypothetical protein
hypothetical protein

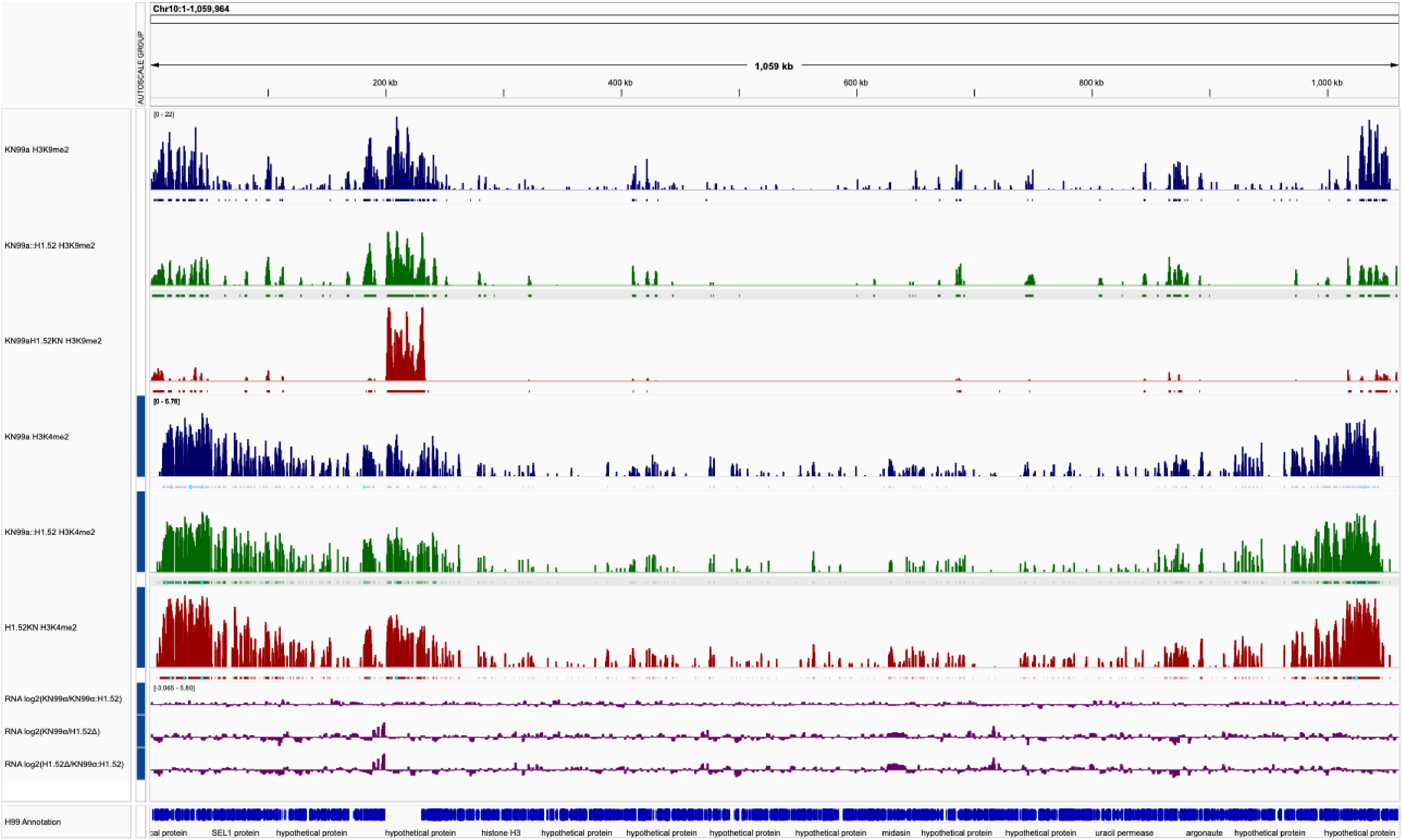

Chr10:1-1,059,964
AUTOSCALE GROUP
1,059 kb
200 kb
400 kb
600 kb
800 kb
1,000 kb
[0 - 22]
KN99a H3K9me2
KN99a::H1.52 H3K9me2
KN99aH1.52KN H3K9me2
[0 - 5.78]
KN99a H3K4me2
KN99a::H1.52 H3K4me2
H1.52KN H3K4me2
[-3.065 - 5.80]
RNA log2(KN99α/KN99α:H1.52)
RNA log2(KN99α/H1.52Δ)
RNA log2(H1.52Δ/KN99α:H1.52)
H99 Annotation
cal protein
SEL1 protein
hypothetical protein
hypothetical protein
histone H3
hypothetical protein
hypothetical protein
hypothetical protein
hypothetical protein
midasin
hypothetical protein
hypothetical protein
uracil permease
argonaute
hypothetical protein
hypothetical protein

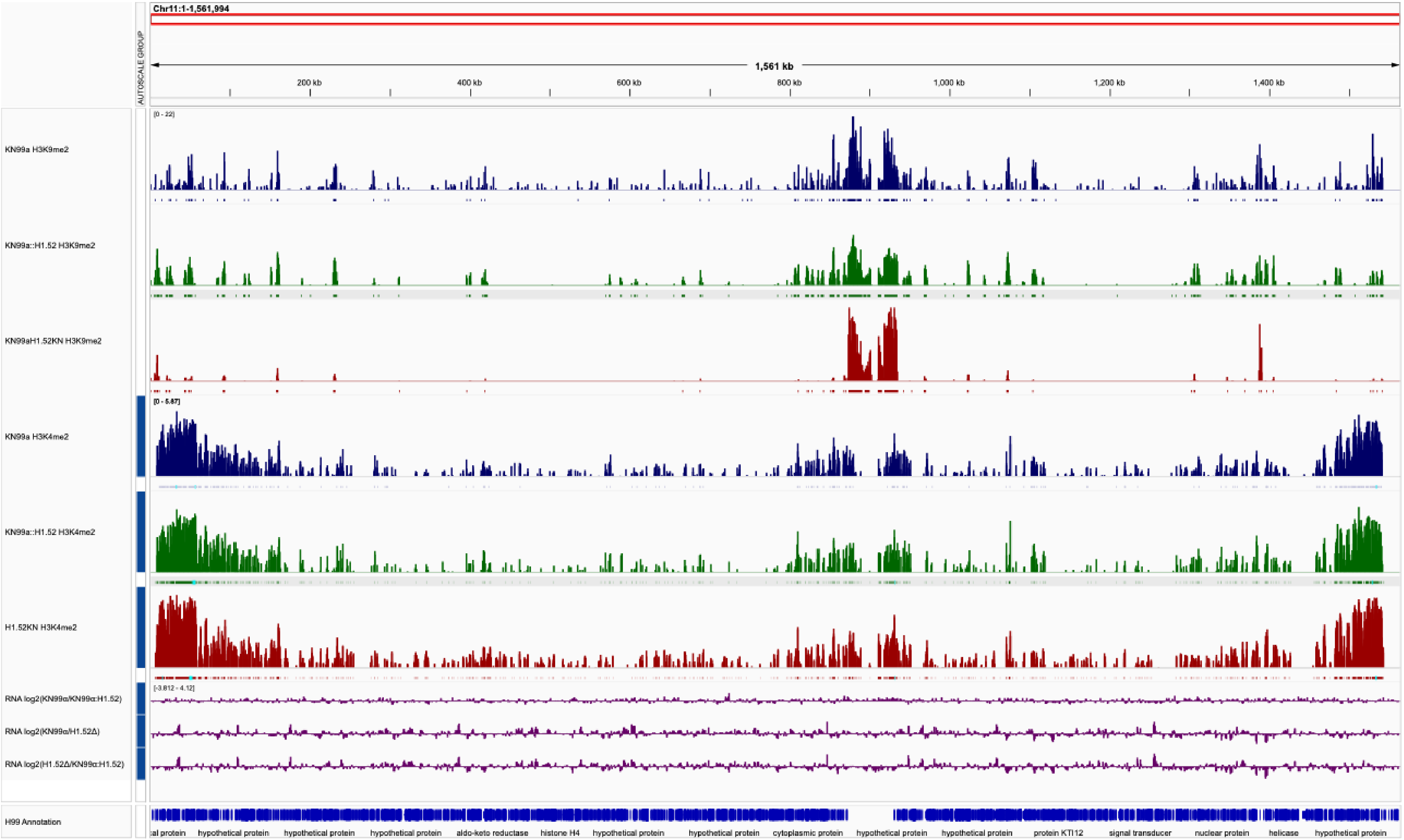

Chr11:1-1,561,994
AUTOSCALE GROUP
1,561 kb
200 kb
400 kb
600 kb
800 kb
1,000 kb
1,200 kb
1,400 kb
[0 - 22]
KN99a H3K9me2
KN99a::H1.52 H3K9me2
KN99aH1.52KN H3K9me2
[0 - 5.87]
KN99a H3K4me2
KN99a::H1.52 H3K4me2
H1.52KN H3K4me2
[-3.812 - 4.12]
RNA log2(KN99α/KN99α:H1.52)
RNA log2(KN99α/H1.52Δ)
RNA log2(H1.52Δ/KN99α:H1.52)
H99 Annotation
al protein
hypothetical protein
hypothetical protein
hypothetical protein
aldo-keto reductase
histone H4
hypothetical protein
hypothetical protein
cytoplasmic protein
hypothetical protein
hypothetical protein
protein KTI12
signal transducer
nuclear protein
helicase
hypothetical protein

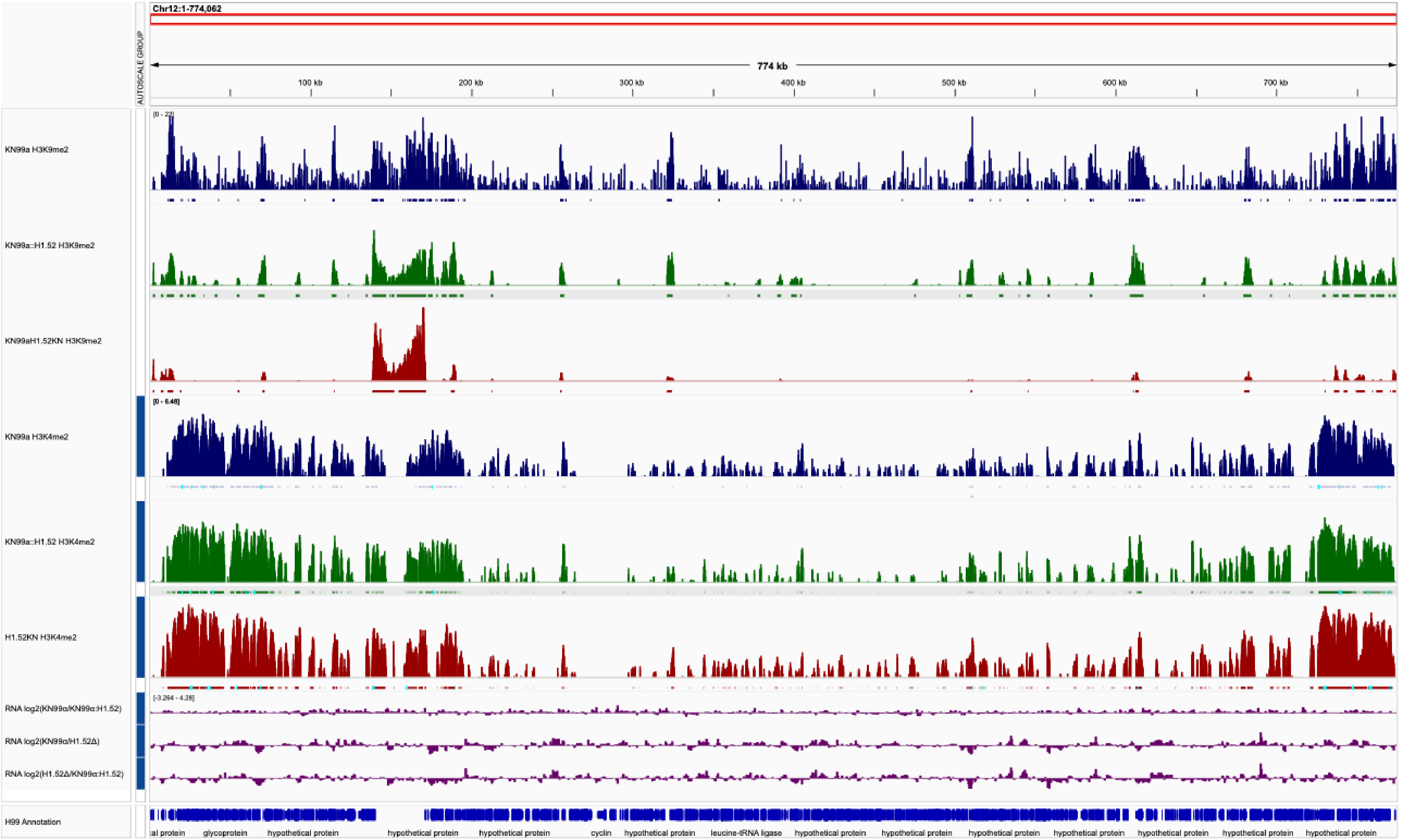
Chr12:1-774,062
AUTOSCALE GROUP
774 kb
100 kb
200 kb
300 kb
400 kb
500 kb
600 kb
700 kb
KN99a H3K9me2
KN99a::H1.52 H3K9me2
KN99aH1.52KN H3K9me2
KN99a H3K4me2
KN99a::H1.52 H3K4me2
H1.52KN H3K4me2
RNA log2(KN99α/KN99α:H1.52)
RNA log2(KN99α/H1.52Δ)
RNA log2(H1.52Δ/KN99α:H1.52)
H99 Annotation
glycoprotein
hypothetical protein
cyclin
leucine-tRNA ligase

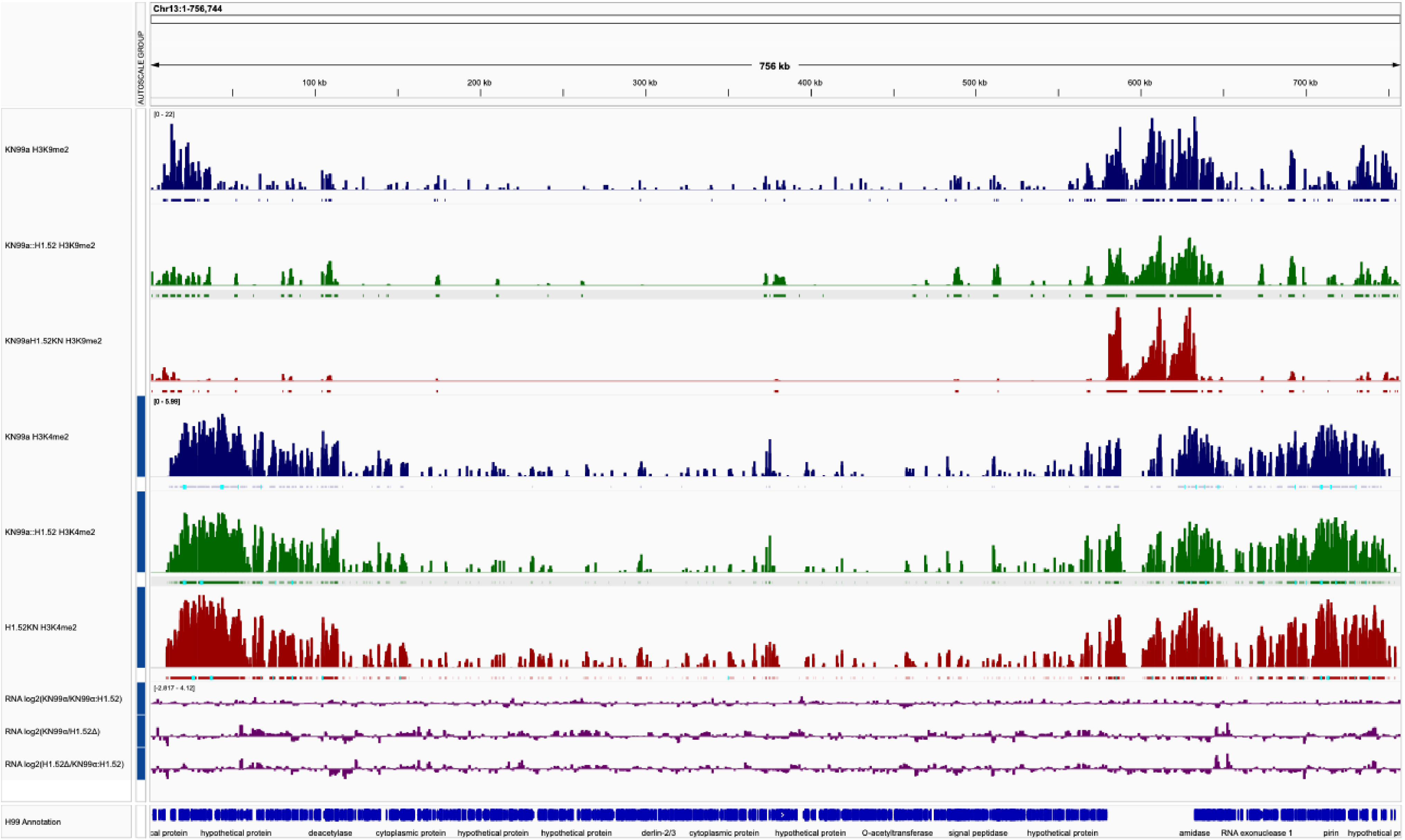

Chr13:1-756,744
756 kb
100 kb
200 kb
300 kb
400 kb
500 kb
600 kb
700 kb
AUTOSCALE GROUP
[0 - 22]
KN99a H3K9me2
KN99a::H1.52 H3K9me2
KN99aH1.52KN H3K9me2
[0 - 5.98]
KN99a H3K4me2
KN99a::H1.52 H3K4me2
H1.52KN H3K4me2
[-2.817 - 4.12]
RNA log2(KN99α/KN99α::H1.52)
RNA log2(KN99α/H1.52Δ)
RNA log2(H1.52Δ/KN99α::H1.52)
H99 Annotation
cal protein
hypothetical protein
deacetylase
cytoplasmic protein
hypothetical protein
hypothetical protein
derlin-2/3
cytoplasmic protein
hypothetical protein
O-acetyltransferase
signal peptidase
hypothetical protein
amidase
RNA exonuclease 1
pirin
hypothetical pr

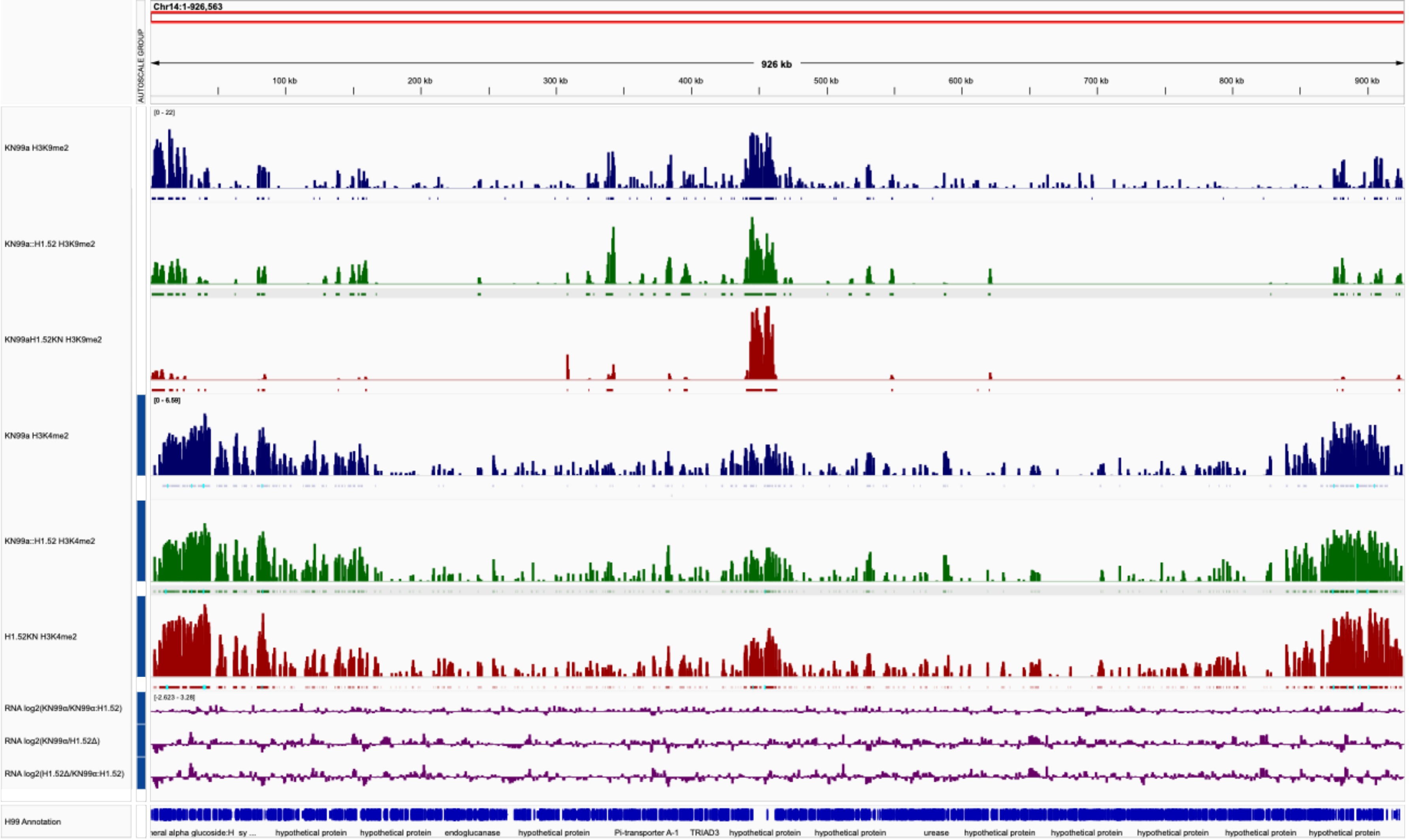

Figure S5. H3K9me2 and H3K4me2 enrichment across all nuclear chromosomes in *C. neoformans*. Fold-enrichment (FE) tracks were generated using MACS3 by normalising IP signal against matched input control and are displayed for all 14 chromosomes. ChIP-seq tracks are coloured by strain: KN99α (blue), *H1.52Δ* (red), and *H1.52Δ:H1.52* complement (green). Tracks displayed beneath each FE signal represent significant peaks called by MACS3. RNA-seq expression tracks (purple) are shown alongside ChIP-seq signal and represent $\log_2$ RPKM-normalised fold changes for three pairwise comparisons: KN99α/*H1.52Δ:H1.52*, KN99α/*H1.52Δ*, and *H1.52Δ/h1.52Δ:H1.52*. Positive values indicate genes upregulated in the numerator strain; negative values indicate downregulated genes.

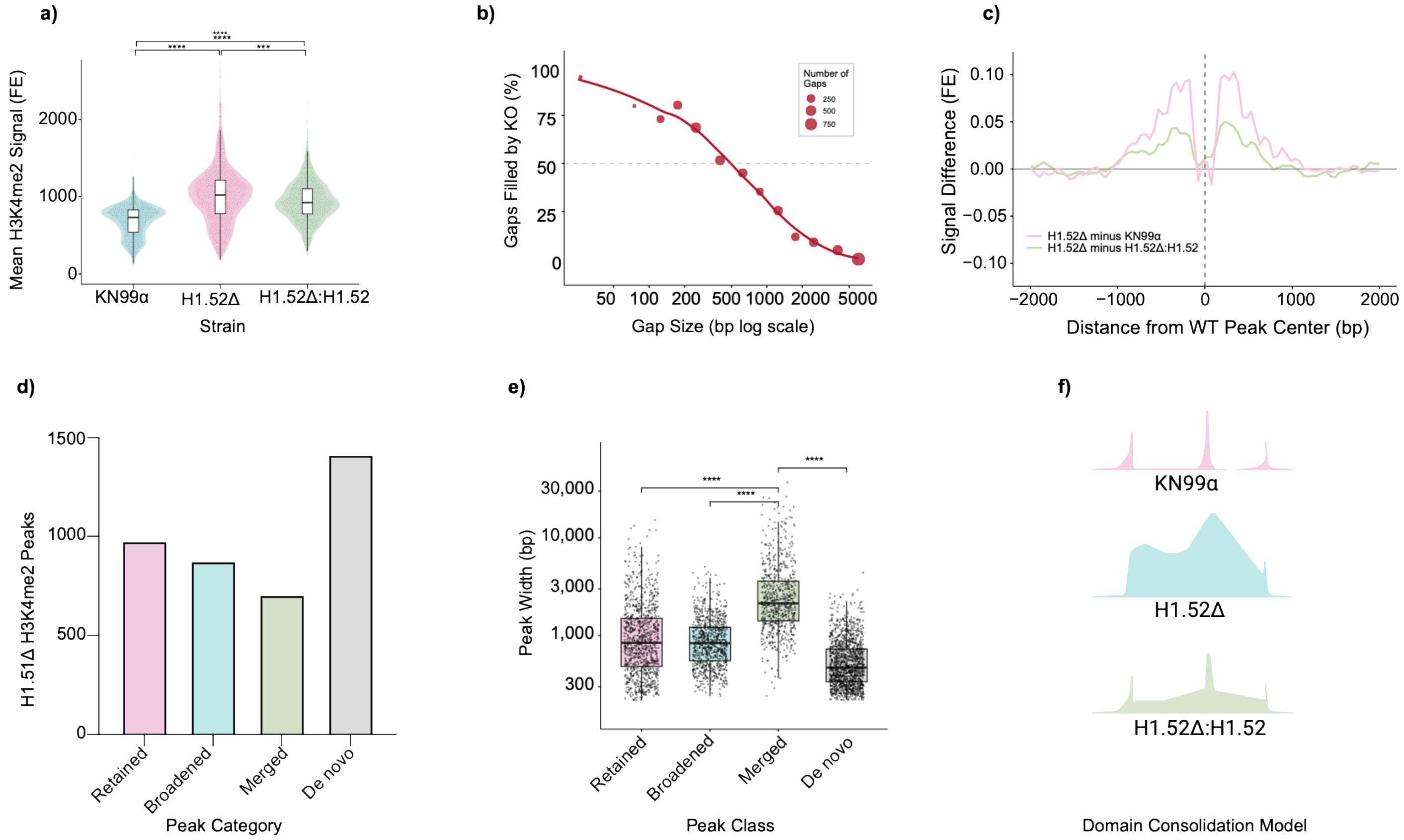

Supplementary Figure 6. H3K4me2 expansion in H1.52Δ reflects lateral spreading from existing domains. **a**, Mean H3K4me2 fold-enrichment (FE) signal in inter-peak gaps (<500 bp) across KN99α, H1.52Δ, and complement strains (n = 1,000 randomly sampled gaps). H1.52Δ shows elevated inter-peak signal versus KN99α ($p = 6.5 \times 10^{-34}$, Wilcoxon rank-sum); complementation partially rescues this ($p = 2.9 \times 10^{-24}$ vs KN99α). Box plots show median and interquartile range. **b**, Gap-filling probability as a function of inter-peak gap size. Point size reflects the number of gaps per bin. Gaps <50 bp are filled in >95% of cases versus <5% for gaps >3 kb. Dashed line indicates 50%. **c**, Meta-profile of H3K4me2 signal centred on KN99α peak midpoints (±2 kb; n = 2,000 peaks). Summit signal is unchanged across strains, confirming lateral rather than apical spreading. **d**, Classification of H1.52Δ peaks by overlap with KN99α peaks. Merged peaks (n = 705) consume 1,839 KN99α peaks (43.4% of total). **e**, Peak width distributions by class (log scale). Merged peaks are significantly wider than all other classes ($p < 10^{-100}$, Wilcoxon rank-sum). ****$p < 0.0001$. **f**, Schematic of H1.52-dependent domain consolidation: H1.52 loss drives lateral H3K4me2 spreading, merging adjacent narrow peaks into unified broad domains; complementation partially restores discrete architecture.
