# Supplementary material for: Neofunctionalization of H1 linker histones drives divergent gene expression and histone methylation in Cryptococcus neoformans
Source: Commun Biol. 2026 Jun 10;9:1232. doi: 10.1038/s42003-026-10432-4 (PMC13594160; doi:10.1038/s42003-026-10432-4)
Supplement: Supplementary file 4 [file 42003_2026_10432_MOESM3_ESM.pdf]

Description of Additional Supplementary Files

File name: Supplementary Data 1

Description: Differentially expressed genes in *Cryptococcus neoformans* KN99α and knockout strains.

File name: Supplementary Data 2

Description: Enriched GO terms and KEGG pathways in H1.52Δ transcriptome

File name: Supplementary Data 3

Description: Annotated chip seq H3K9me2 and H3K4me2 peaks called in strains.

File name: Supplementary Data 4

Description: Primers used in this study for generating complimented strains and for PCR.

File name: Supplementary Data 5

Description: Nucleic acid quality assessment. RIN/DIN values and TapeStation electropherogram profiles for RNA and ChIP-DNA samples submitted for sequencing.

File name: Supplementary Data 6

Description: RNA-Seq alignment statistics for *Cryptococcus neoformans* KN99α and knockout strains.

File name: Supplementary Data 7

Description: Fraction of Reads in Peaks (FRiP), library size, and complexity quality metrics for ChIP-seq experiments.

File name: Supplementary Data 8

Description: Source data underlying all graphs and charts presented in the main and supplementary figures.
